# Stimulants for disorders of consciousness in the intensive care unit: a randomized, placebo-controlled trial

**DOI:** 10.1093/brain/awaf228

**Published:** 2025-06-12

**Authors:** Marwan H Othman, Attila Géry Toury-Puel, Karen Irgens Tanderup Hansen, Moshgan Amiri, Pardis Zarifkar, Costanza Peinkhofer, Sarah Gharabaghi Stückler, Markus Harboe Olsen, Jens Bjerregaard, Margit Smitt, Anna Søgaard Magnussen, Axel Forsse, Jacob Møller, Marie Katrine Klose Nielsen, Cecilie Høgfeldt Jessen, Christian Hassager, Simon Hyttel-Sørensen, Anders Perner, Morten Hylander Møller, Peter Hasse Møller-Sørensen, John Hauerberg, Peter Birkeland, Sigurdur Thor Sigurdsson, Christian Aage Wamberg, Theis Skovsgaard Itenov, Christian Sylvest Meyhoff, Kirsten Møller, Tobias S Andersen, Jesper Kjaergaard, Daniel Kondziella

**Affiliations:** Department of Neurology, Copenhagen University Hospital - Rigshospitalet, Copenhagen 2100, Denmark; Department of Applied Mathematics and Computer Science, Technical University of Denmark, Kgs. Lyngby 2800, Denmark; Department of Neurology, Copenhagen University Hospital - Rigshospitalet, Copenhagen 2100, Denmark; Department of Neurology, Copenhagen University Hospital - Rigshospitalet, Copenhagen 2100, Denmark; Department of Neurology, Copenhagen University Hospital - Rigshospitalet, Copenhagen 2100, Denmark; Department of Neurology, Copenhagen University Hospital - Rigshospitalet, Copenhagen 2100, Denmark; Department of Neurology, Copenhagen University Hospital - Rigshospitalet, Copenhagen 2100, Denmark; Department of Neuroanaesthesiology, Copenhagen University Hospital - Rigshospitalet, Copenhagen 2100, Denmark; Department of Neuroanaesthesiology, Copenhagen University Hospital - Rigshospitalet, Copenhagen 2100, Denmark; Department of Neuroanaesthesiology, Copenhagen University Hospital - Rigshospitalet, Copenhagen 2100, Denmark; Department of Neurosurgery, Copenhagen University Hospital - Rigshospitalet, Copenhagen 2100, Denmark; Department of Neurosurgery, Copenhagen University Hospital - Rigshospitalet, Copenhagen 2100, Denmark; Department of Clinical Medicine, University of Copenhagen, Copenhagen 2200, Denmark; Department of Cardiology, Copenhagen University Hospital - Rigshospitalet, Copenhagen 2100, Denmark; Department of Forensic Medicine, Faculty of Health and Medical Sciences, University of Copenhagen, Copenhagen 2100, Denmark; Department of Forensic Medicine, Faculty of Health and Medical Sciences, University of Copenhagen, Copenhagen 2100, Denmark; Department of Clinical Medicine, University of Copenhagen, Copenhagen 2200, Denmark; Department of Cardiology, Copenhagen University Hospital - Rigshospitalet, Copenhagen 2100, Denmark; Department of Intensive Care, Copenhagen University Hospital - Rigshospitalet, Copenhagen 2100, Denmark; Department of Clinical Medicine, University of Copenhagen, Copenhagen 2200, Denmark; Department of Intensive Care, Copenhagen University Hospital - Rigshospitalet, Copenhagen 2100, Denmark; Department of Clinical Medicine, University of Copenhagen, Copenhagen 2200, Denmark; Department of Intensive Care, Copenhagen University Hospital - Rigshospitalet, Copenhagen 2100, Denmark; Department of Cardiothoracic Anaesthesiology, Copenhagen University Hospital - Rigshospitalet, Copenhagen 2100, Denmark; Department of Neurosurgery, Copenhagen University Hospital - Rigshospitalet, Copenhagen 2100, Denmark; Department of Neurosurgery, Copenhagen University Hospital - Rigshospitalet, Copenhagen 2100, Denmark; Department of Neuroanaesthesiology, Copenhagen University Hospital - Rigshospitalet, Copenhagen 2100, Denmark; Department of Anaesthesia and Intensive Care, Copenhagen University Hospital - Bispebjerg and Frederiksberg, Copenhagen 2400, Denmark; Department of Clinical Medicine, University of Copenhagen, Copenhagen 2200, Denmark; Department of Anaesthesia and Intensive Care, Copenhagen University Hospital - Bispebjerg and Frederiksberg, Copenhagen 2400, Denmark; Department of Clinical Medicine, University of Copenhagen, Copenhagen 2200, Denmark; Department of Anaesthesia and Intensive Care, Copenhagen University Hospital - Bispebjerg and Frederiksberg, Copenhagen 2400, Denmark; Department of Neuroanaesthesiology, Copenhagen University Hospital - Rigshospitalet, Copenhagen 2100, Denmark; Department of Clinical Medicine, University of Copenhagen, Copenhagen 2200, Denmark; Department of Applied Mathematics and Computer Science, Technical University of Denmark, Kgs. Lyngby 2800, Denmark; Department of Clinical Medicine, University of Copenhagen, Copenhagen 2200, Denmark; Department of Cardiology, Copenhagen University Hospital - Rigshospitalet, Copenhagen 2100, Denmark; Department of Neurology, Copenhagen University Hospital - Rigshospitalet, Copenhagen 2100, Denmark; Department of Clinical Medicine, University of Copenhagen, Copenhagen 2200, Denmark

**Keywords:** apomorphine, brain injury, coma, consciousness, clinical trial, methylphenidate, pupillometry

## Abstract

In the intensive care unit (ICU), management of unresponsive patients with brain injury focuses on preventing secondary brain damage. Therapeutic strategies that directly promote the recovery of consciousness are urgently needed. In an investigator-initiated, randomized, placebo-controlled, double-blind, cross-over trial, we studied the effects of apomorphine and methylphenidate in ICU patients with acute disorders of consciousness (DoC). We hypothesized that these stimulants would improve consciousness biomarkers assessed by automated pupillometry (primary outcome) and clinical signs of consciousness (secondary outcome).

We randomized 50 ICU patients with DoC (14 female; mean age 63 ± 10 years; 48 with non-traumatic brain injuries) to strata consisting of three consecutive treatment sessions during which apomorphine, methylphenidate or placebo were administered. In total, we administered 112 study medications, including 36 doses of apomorphine, 39 doses of methylphenidate and 37 doses of placebo. Missing administrations were due to death, ICU discharge or spontaneous consciousness recovery. Plasma concentrations of stimulants confirmed drug exposure. We found no adverse events related to the trial drugs.

Pupillometry recordings of sufficient quality (*n* = 590) were available from 48 (96%) patients. A pupillary response to a verbal arithmetic command (i.e. ≥3 pupillary dilations on five verbal arithmetic tasks) was identified during 70 (12%) of these recordings. Seven (15%) patients without any other observable response to spoken commands also passed a stricter threshold of ≥4 pupillary dilations, suggesting cognitive motor dissociation. Apomorphine [odds ratio (OR) 1.35, 95% confidence interval (CI): 0.93 to 1.96] and methylphenidate (OR 1.29, 95% CI: 0.89 to 1.86) did not significantly increase pupillary responses. However, after study drug administration, 10 (20%) patients showed improved clinical arousal at least once. Signs of arousal were noted after one dose of placebo, four doses of apomorphine (OR 5.04, 95% CI: 0.56 to 120.7) and seven doses of methylphenidate (OR 9.96, 95% CI: 1.36 to 235.8). Changes toward higher consciousness level categories were observed once after placebo, four times after apomorphine (OR 5.67, 95% CI 0.63 to 169.46) and three times after methylphenidate (OR 3.41, 95% CI 0.34 to 88.00). In a *post hoc* analysis, patients with greater pupillary responsiveness showed better arousal, suggesting that this condition may predict stimulant drug effects.

In conclusion, while pupillometry revealed no direct drug effects on overall pupillary responses, stimulants may have triggered clinical arousal in some patients, particularly in those with greater pupillary responsiveness. These findings require replication but should guide future pharmacological trials aimed at improving consciousness recovery after brain injury.

## Teaser

Othman *et al.* studied the impact of apomorphine and methylphenidate on consciousness biomarkers and clinical arousal in intensive care unit patients with brain injury. Treatment did not alter overall pupillary responses, but some patients had transiently improved arousal, particularly those with greater pupillary responsiveness.

## Introduction

Every year, coma affects millions of people with acute brain injury worldwide.^1^ The key question after brain injury leading to coma is: Will the patient regain consciousness, and what can be done to improve the odds? It is crucial for patients with coma and other disorders of consciousness (DoC) that signs of consciousness are detected as early as possible, as the recovery of consciousness in the intensive care unit (ICU) is the most critical prerequisite for neurorehabilitation and long-term outcome.^2,3^ Evidence suggests that 10%–25% of clinically unresponsive acute DoC patients are covertly conscious, i.e. at the bedside they only appear to be comatose or in an unresponsive wakefulness state (UWS).^4,5^ During clinical exam, these patients cannot show that they are aware because they have lost the necessary motor function to do so. However, they have volitional brain responses to motor imagery or verbal commands detectable on functional MRI,^6^ EEG^5,7^ or pupillometry.^8^ This state of cognitive motor dissociation (CMD) is crucial for the management of acute brain injury and has been identified as one of the most important contemporary issues in neurocritical care.^9,10^

Despite the unresolved need for precise diagnostic methodologies to identify residual consciousness, research in chronic (but not acute) DoC has progressed toward therapeutic trials aiming to boost consciousness recovery. Pharmacological stimulants like apomorphine and methylphenidate stimulate arousal and awareness in chronic DoC with no or low risks of severe adverse effects.^11-16^ Apomorphine is a potent dopamine agonist with direct stimulatory effects on D1 and D2 receptors. It is indicated for Parkinson’s disease patients when treatment with levodopa no longer sufficiently ameliorates motor deficits. In case reports and small studies of chronic DoC patients with traumatic brain injury, improved neurological behaviour was observed within days to weeks after daily infusion of subcutaneous apomorphine.^12,13^ Similarly, methylphenidate is a sympathomimetic stimulant that increases synaptic concentrations of dopamine and norepinephrine by inhibiting reuptake in the striatum. In a retrospective cohort study, post-cardiac arrest patients treated with either methylphenidate or amantadine demonstrated possible improvements in command-following abilities, survival to discharge and modified Rankin scale scores, but the sample size was small (*n* = 16).^17^ Additionally, traumatic brain injury patients treated with methylphenidate twice daily had shorter ICU stays.^18^ Although the clinical effect size is modest, it seems that stimulants can improve functional and cognitive indices in some DoC patients.^11,15^

Neither apomorphine nor methylphenidate have been prospectively studied in randomized trials of acute DoC in the ICU. To address this gap, we conducted a randomized trial of apomorphine and methylphenidate in acute DoC patients admitted with traumatic or non-traumatic brain injury. We hypothesized that these stimulants given as a ‘challenge test’ temporarily improve consciousness biomarkers as assessed by automated pupillometry,^8,19,20^ and lead to temporary arousal in a subset of acute DoC patients. The idea is that DoC patients may have cerebral reserves that can be activated for prognostication and rehabilitation.

## Materials and methods

### Trial design

**Figure 1:**
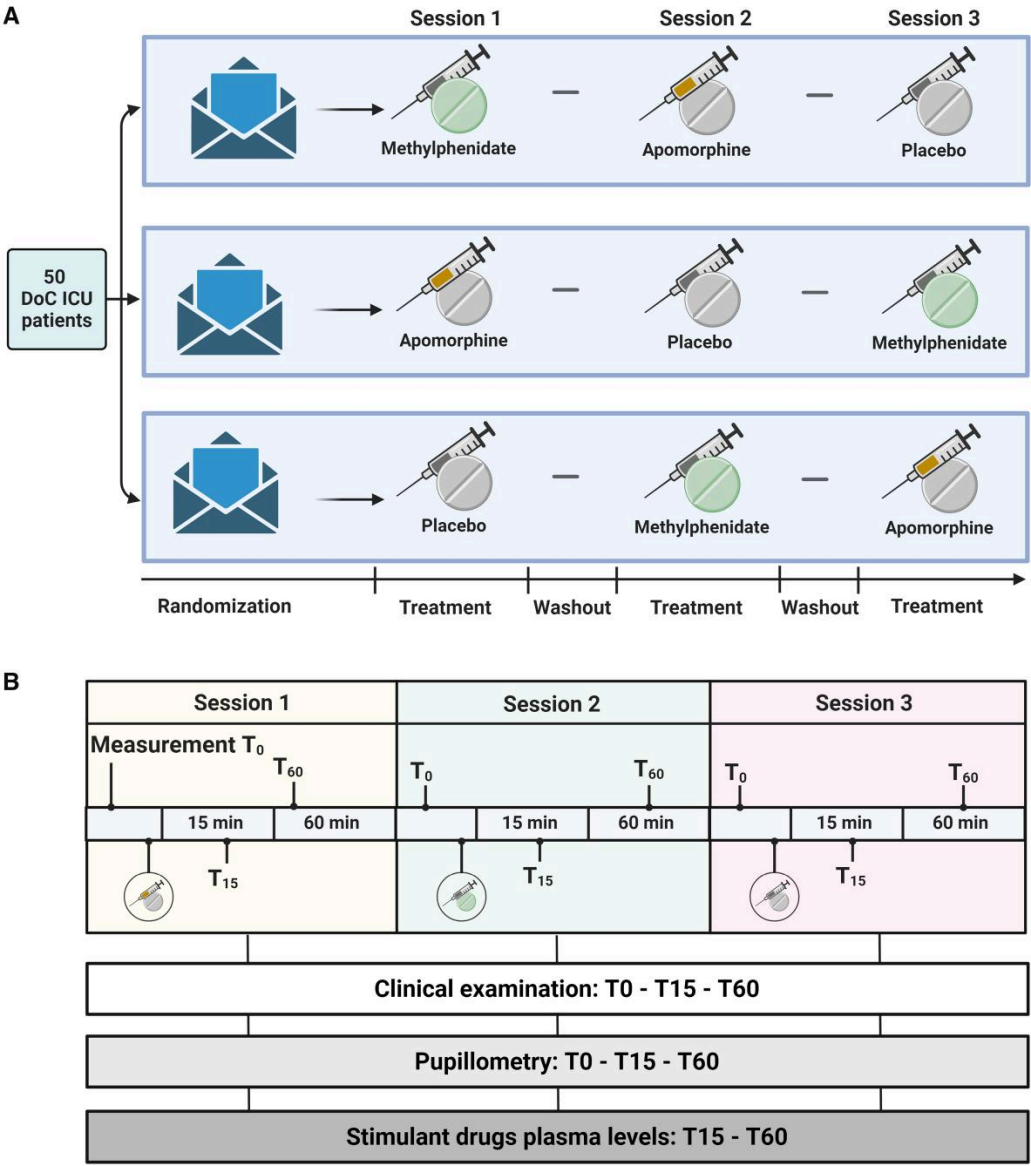
**Study overview.** In an investigator-initiated, randomized, placebo-controlled, double-blind, cross-over trial, we investigated the effects of apomorphine and methylphenidate on clinical consciousness levels and consciousness biomarker in acute DoC patients with traumatic or non-traumatic brain injury in the ICU. This figure depicts the study design (**A**) and study outcomes (**B**). DoC = disorders of consciousness; ICU = intensive care unit. Created in BioRender. Kondziella, D. (2025) https://BioRender.com/niebd75.

This was an investigator-initiated, randomized, placebo-controlled, double-blind, cross-over trial to evaluate apomorphine and methylphenidate in brain injury patients with acute DoC. The objectives were to investigate the effects of 20 mg oral methylphenidate and 2 mg subcutaneous apomorphine in acute DoC patients on pupillary responses revealed by automated pupillometry, as well as clinical consciousness levels. Primary outcome was pupillary dilation during a mental arithmetic paradigm. The secondary outcomes were the numbers of patients with (i) improved clinical arousal; and (ii) a shift toward a higher consciousness level category (Fig. 1). A detailed study protocol has been published.^21^

### Study participants

Adult patients with acute DoC due to traumatic or non-traumatic brain injury were prospectively recruited from the neurocritical, cardiological, cardiothoracic and general ICUs of a tertiary referral centre (Copenhagen University Hospital—Rigshospitalet) and the general ICU of another academic hospital (Copenhagen University Hospital—Bispebjerg Hospital), both in Copenhagen, Denmark. ICUs were screened daily for consecutive patients eligible for trial participation except for weekends, holidays and other times of leave.

Inclusion criteria were: (i) age ≥18 years; (ii) patients with severe acute traumatic or non-traumatic brain injury in an unresponsive state (coma, UWS) or a minimally conscious state (MCS, which was further graded into MCS minus and MCS plus^22^) according to the Full Outline of UnResponsiveness (FOUR^23^) and Simplified Evaluation of CONsciousness Disorders (SECONDs^24^) scales; and (iii) written informed consent for trial participation from next-of-kin and trial guardians. Exclusion criteria were: (i) recovery of the ability to follow observable commands prior to enrolment; (ii) pre-existing mental or severe physical impairments; (iii) deafness or eye disease interfering with pupillometry studies; (iv) use of dopamine agonists or antagonists within six half-lives of the drug; (v) use of psychoactive or psychotropic substances within six half-lives of the drug; and (vi) clinically unstable patients requiring immediate medical attention.

We aimed to study patients without sedation. When patients could not fully be weaned from sedation, dosages were reduced to the lowest possible level. Levels of sedation were graded as described earlier^25,26^ (Supplementary material, ‘Methods’ section 1).

### Randomization, allocation concealment and blinding

We used stratified block randomization with strata of ≤UWS and ≥MCS, each block consisting of six treatment sequences. Using the ‘randomizeR package’ in R (R Core Team, Vienna, Austria, 2023), we generated an Excel file for the REDCap (Research Electronic Data Capture) randomization module. Upon study inclusion, patients were randomly allocated to a schedule of three consecutive treatment sessions (Fig. 1). The investigators, medical staff, patients, next-of-kin and trial guardians were blinded to the allocation table. Packaging of the study drugs was coordinated by staff from the hospital pharmacy who did not otherwise participate in the trial or the patient treatment.

### Study drug administration

For each session, treatment included either: (i) 20 mg methylphenidate tablet suspended in water and administered via a nasogastric tube; (ii) 2 mg subcutaneous apomorphine; or (iii) saline as placebo, administered via a nasogastric tube or subcutaneous injections. To ensure double blinding, alongside each given study drug, we administered subcutaneous placebo injections matched to the apomorphine fluid volume or placebo tablets suspended in water, which were matched to the methylphenidate fluid volume. For example, if the primary medication was an injection, a placebo tablet was suspended in water and administered via a nasogastric tube and vice versa. We always administered 0.4 ml of fluid (either saline or apomorphine) from a 1 ml syringe as well as 8 ml of fluid with a suspended tablet (either saline or methylphenidate) from a 10 ml gavage syringe. Patients underwent a baseline examination (T0) that included a neurological examination and automated pupillometry measurements in combination with active cognitive paradigms before receiving the randomized drug. Subsequently, repeated assessments were performed 15 min (T15) and 60 min (T60) after drug administration. The time points included the presumed peak plasma concentration of apomorphine and methylphenidate, respectively. Each treatment session thus involved the administration of one of three arms (two of which contained a stimulant drug). We allowed a washout period of at least 10 h in between treatment sessions to minimize the risk of carry-over effects. By completing a baseline followed by two assessments, patients served as their own controls, reducing the risk of natural recovery as a time-modified confounding factor.^27^

### Measurement of plasma apomorphine and methylphenidate levels

To correlate the pupillary responses clinical effects with the actual drug levels in the blood, plasma samples of apomorphine and methylphenidate were analysed at T15 and T60 using ultra-high-performance liquid chromatography–tandem mass spectrometry (Supplementary material, ‘Methods’ section 2).

### Automated pupillometry and cognitive paradigms

We^8,19^ and others^28^ have shown that automated pupillometry can detect pupillary responses in clinically unresponsive subjects exposed to mental arithmetic tasks. This includes the possibility of detecting CMD in ICU patients with acute brain injuries, using a paradigm based on automated pupillometry combined with mental arithmetic.^8,19^ Briefly, acute DoC patients are asked to engage in mental arithmetic and relax, five times in succession. The mental arithmetic tasks consist of five sets of moderate (4 × 36, 8 × 32, 3 × 67, 6 × 37, 7 × 43) and five sets of high (21 × 22, 33 × 32, 55 × 54, 43 × 44, 81 × 82) complexity tasks. The prerecorded instructions were played through headphones. Task duration was set to 25 s with 20 s of rest in between. As previously described,^8^ we chose the following cut-off levels for pupillary responses and CMD, respectively: (i) Pupillary response was defined as three or more pupillary dilations on five mental arithmetic tasks in one set (moderate or hard mental arithmetic). For sub-analysis, we also looked at a stricter threshold of at least four pupillary dilations on five mental arithmetic tasks in one set.(ii) Evidence of CMD was defined as at least four pupillary dilations on five mental arithmetic tasks in patients without any other observable response to spoken commands (i.e. patients who were clinically comatose, UWS or MCS minus^9^).

We recorded pupillary responses using the NeurOptics PLR-3000 pupillometer (NeurOptics).^8,19^ The PLR-3000 pupillometer records pupil diameter over time and initially displays the information as a graph on the device. Pupillary dilation was defined as a significantly larger pupil diameter during mental arithmetic compared to rest before and after a stimulus/task. See Supplementary material, ‘Methods’ section 3 and previous publications for details.^8,19^

### Clinical examination of consciousness levels

All patients were examined at inclusion and during each study session using the FOUR and SECONDs to determine the correct DoC category: coma, UWS, MCS minus/plus or emerged from MCS. See Supplementary Table 1 for definitions and references regarding DoC classifications, including CMD. After application of the study drug, the patient was continuously observed for 60 min for clinical improvement (yes—no). If (and only if) the blinded investigator had the clinical impression of improved arousal, the patient was re-examined immediately, including using the FOUR and SECONDs, to confirm clinical improvement and to identify any objective shifts in consciousness classification (e.g. from UWS before the study drug to MCS minus after the drug). Observations were also documented using free text descriptions. Beneficial effects on consciousness were defined as (i) any clinically observable arousal verified by an increased FOUR and/or SECONDs score; and (ii) improved arousal with a change toward a higher consciousness level category (e.g. from UWS to MCS minus).

### Monitoring for safety

All patients were closely monitored for adverse events, serious adverse events and reactions and suspected unexpected serious adverse reactions until six half-lives of the active substance with the longest plasma lifetime (i.e. methylphenidate with 3 h) had passed. This process was externally controlled by staff from the Good Clinical Practice (GCP) unit in Copenhagen, Denmark. GCP staff also monitored all other regulatorily relevant trial aspects before, during and after the trial.

### Additional investigations

Per protocol,^21^ we performed near-infrared spectroscopy (NIRS) along with EEG to investigate neurovascular coupling; these data will be published separately.

### Ethics

The study was approved by the Capital Region of Denmark ethics committee (EudraCT Number: 2021-001453-31) and followed the principles of the Declaration of Helsinki. Additional approval was obtained from the Danish Board on Medicines and Drugs and the Danish Data Protection Agency. Written informed consent was obtained from next-of-kin and trial guardians.

### Sample size estimation

The sample size estimation was based on separate sample size calculations for each outcome and selection of the largest size of necessary subjects (*n* = 41 patients with *n* = 123 study drug administrations). We adjusted the sample size by 20% to compensate for potential study drop-outs (total *n* = 50 patients with *n* = 150 administrations). Details are available from the protocol paper.^21^

### Statistical analysis: general aspects

Categorical data are presented as *n* (percentages), while quantitative data are presented as either median (interquartile range, IQR) or mean ± standard deviation. Data were checked for normal distribution using histograms and the Shapiro-Wilk test. Parametric and non-parametric statistical tests were used as appropriate. Tests were two-sided, and *P* < 0.05 was considered statistically significant. We did not adjust for multiplicity testing given the exploratory nature of the study. All analyses were conducted using R statistical software v. 4.3.2.

### Statistical analysis of pupillometry data

We usedgeneralized linear mixed models (GLMM)^29^ with adaptive Gauss-Hermite quadrature (nAGQ = 100) using the lme4 package in R to evaluate the impact of drug interventions on pupillary responses at different time points (T15 and T60) and across active paradigms. The fixed effects considered were drug interventions (apomorphine, methylphenidate, or placebo) and the assessment times (T0–T60). This allowed for a precise analysis of how interventions influenced the probability of achieving higher pupillary dilation scores compared to baseline. Model parameters were estimated using maximum likelihood techniques appropriate for the binomial distribution, modelling the probability of success in mental arithmetic tasks, defined as the number of pupillary dilations (e.g. 4 of 10 combined tasks). To account for within-subject variability across repeated measures, a random intercept for each subject was included in the model. The model converged successfully, and assumptions of the GLMM were evaluated through various diagnostic checks. The uniformity of residuals was confirmed, and the residuals showed no significant deviations from expected patterns. Given only a mild degree of overdispersion (1.28), we proceeded with the model. Sensitivity analyses were performed to account for potential confounders such as sex and sedation levels. However, the inclusion of age in the model led to convergence issues, and thus this was excluded from the final analysis. Additionally, testing a simpler GLM model without random effects was used for further analysis. Data are reported as odds ratios (OR) with 95% confidence intervals (CI). The R code and outputs are available in Supplementary material, ‘Methods’ section 4.

### Statistical analysis of drug effects on arousal and consciousness levels

To probe for improvements in arousal and changes toward a higher consciousness level category with stimulant drug exposure, we employed GLMM with nAGQ = 100. Two separate GLMMs were developed: one to assess improved arousal and another to evaluate changes toward a higher consciousness level category. Both models included drug intervention as a fixed effect and participants as a random effect to account for repeated measures within subjects. The GLMMs successfully converged and met all assumption criteria as validated through diagnostic checks: no overdispersion was detected; residuals were uniformly distributed; and no outliers were identified, confirming the validity of the models. Adjustments taking the level of age, sex and sedation into account were made for sensitivity analyses. In addition, a simpler GLM was used for further sensitivity analyses. In the following, we report GLMMs because of their ability to account for variability between subjects. Data are reported as OR with 95% CI. See Supplementary material, ‘Methods’ section 5 for R code and outputs.

## Results

**Figure 2:**
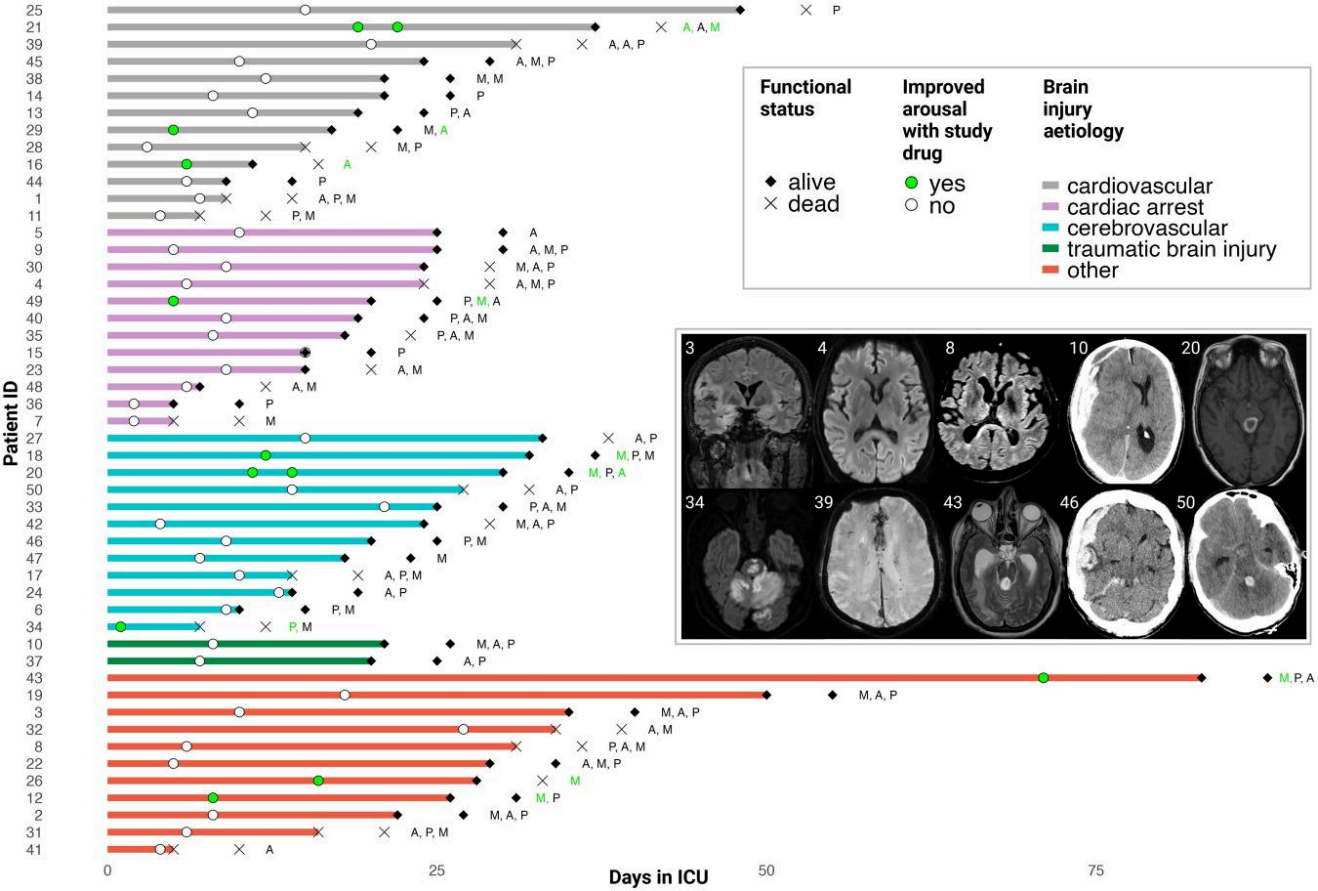
**Swimmer plot of time in the ICU for 50 DoC patients**. Patients are ordered according to the type of brain injury and length of ICU stay. The plot shows key events during the time course of the study, including day of study enrolment, study drug administrations and functional status at ICU discharge and at 3 months (alive versus dead). Improved arousal during study drug application is highlighted (green circles). Of 50 patients enrolled in the study, 10 showed clinically improved arousal at least once after administration of a study drug (Patients 20 and 21 showed improvement twice). A = apomorphine; M = methylphenidate; P = placebo. *Inset*: Selected neuroimaging pathologies, including cortical and/or subcortical injury related to herpes encephalitis (coronal FLAIR, Patient 3), post-cardiac arrest encephalopathy (axial DWI, Patient 4), carbon monoxide poisoning (axial DWI, Patient 8), traumatic brain injury (axial CT, Patient 10), intracranial mesencephalic haemorrhage (axial T1, Patient 20), ischaemic stroke from a basilar artery occlusion (axial DWI, Patient 34), cerebral microbleeds associated with extracorporeal membrane oxygenation after major thoracic surgery (axial SWI, Patient 39), obstructive hydrocephalus with cerebral metastasies (axial T2, Patient 43), aneurismal subarachnoid haemorrhage with subdural extension (axial CT, Patient 46) and intracerebral haemorrhage from an arteriovenous malformation with obstructive hydrocephalus (axial CT, Patient 50). DWI = diffusion weighted imaging; FLAIR = fluid attenuated inversion recovery; ICU = intensive care unit; SWI = susceptibility weighted imaging.

**Figure 3:**
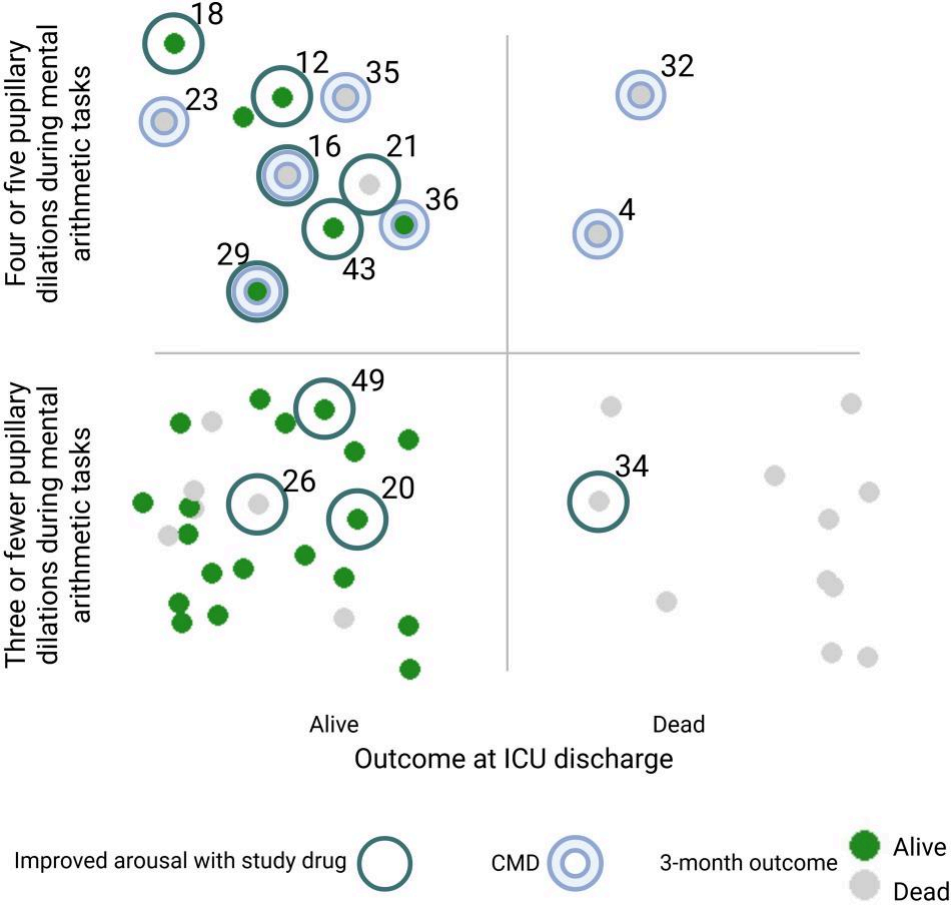
**Drug responses, pupillary dilations and clinical outcomes.** Improved arousal with a study drug was mostly seen in patients alive at ICU discharge and in those with four or five pupillary dilations during mental arithmetic tasks, including patients fulfilling CMD criteria. Numbers indicate Patient IDs. CMD = cognitive motor dissociation; ICU = intensive care unit.

Between 13 August 2022 and 17 November 2023, we enrolled 50 acute DoC patients (14 female, 28.0%; mean age 63.2 ± 9.6 years). Forty-eight (96%) patients had non-traumatic brain injuries. Forty-two (84%) patients had a pre-admission modified Rankin scale ≤2. Nine (18%) patients received low to moderate sedation during investigations, comprising 19 (17%) study drug administrations. Twenty-six (52%) patients were alive at 3-month follow-up. Table 1 and Fig. 2 provide demographic and clinical characteristics. Outcomes at ICU discharge and at 3 months are shown in Fig. 3. Individual patient characteristics are available from Supplementary Table 2.

**Table 1:** Demographic and clinical characteristics

	Total (*n* = 50)
Demographics	
Age, years, mean (SD)	63.2 (9.6)
Sex, male, *n* (%)	36 (72)
Admissions, *n* (%)	
Rigshospitalet	
Neurointensive care unit	14 (28)
General ICU	13 (26)
Cardiothoracic ICU	10 (20)
Cardiac ICU	7 (14)
Bispebjerg Hospital	
General ICU	6 (12)
mRS baseline, *n* (%)	
0–2	42 (84.0)
>2	4 (8.0)
Undisclosed	4 (8.0)
Cause of ICU admission, *n* (%)	
Cardiac arrest	12 (24.0)
Cardiovascular—Other^a^	12 (24.0)
Traumatic brain injury/SDH	2 (4.0)
Subarachnoid haemorrhage	2 (4.0)
Intracerebral haemorrhage	5 (10.0)
Acute ischaemic stroke	5 (10.0)
Other^b^	12 (24.0)
Initial level of consciousness, *n* (%)	
Coma	7 (14)
UWS	21 (42)
MCS minus	17 (34)
MCS plus	5 (10)
Pupillometry examinations, *n* (%)^c^	
Mental arithmetic (moderate)	299 (89.0)
Mental arithmetic (hard)	291 (86.6)
Sedation, *n* (%)	
None to minimal	41 (82)
Low to moderate	9 (18)
High or very high	0

ICU = intensive care unit; MCS = minimally conscious state; mRS = modified Rankin scale; SD = standard deviation; SDH = subdural haemorrhage; UWS = unresponsive wakefulness syndrome.

^a^Other causes, cardiovascular: perioperative hypoperfusion, including during transplant surgeries (*n* = 6), aorta dissection (4), aorta aneurysm rupture (1) and heart failure (1).

^b^Other causes: herpes encephalitis (*n* = 4), brain tumour (2), anoxic ischaemic brain damage due to carbon monoxide poisoning (1), epilepsy (1), respiratory failure, including COVID-19 (2), metabolic disorder (1) and sepsis (1).

^c^Data with sufficient quality from a total of 336 possible recordings (112 study drug administrations, recordings at T0, T15 and T60).

### Study drug administrations

A total of 112 study drug doses were administered to the 50 participants, including 36 doses of apomorphine, 39 of methylphenidate and 37 of placebo (Fig. 2). Twenty-seven patients did not receive all three study drugs, resulting in 38 missing administrations. Reasons were ICU discharge to another facility (nine patients, 12 missing administrations), transition to palliative care/death (three patients, five missing administrations), recovery of consciousness (nine patients, 13 missing administrations), medical or surgical deterioration with or without death (four patients, five missing administrations) and other logistical challenges (two patients, three missing administrations).

### Plasma concentrations of stimulant drugs and adverse events

**Figure 4:**
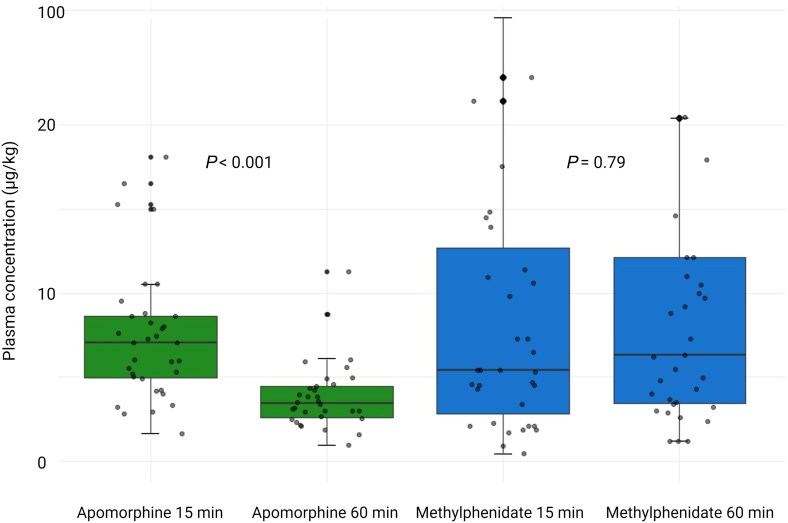
**Plasma concentration of stimulant drugs.** This figure shows box plots of drug levels and a superimposed strip chart of individual data-points. Plasma concentrations of methylphenidate remained stable for a longer period than levels of apomorphine.

Mean plasma concentration of apomorphine was 7.42 ± 3.96 μg/kg at T15 and 3.88 ± 2.04 μg/kg at T60 (*P* < 0.001, paired Student’s *t*-test); mean plasma concentration of methylphenidate was 11.07 ± 17.03 μg/kg at T15 and 11.06 ± 11.65 μg/kg at T60 (*P* = 0.79; Fig. 4). Plasma concentration measurements identified three protocol deviations: Patients 21 and 39 received apomorphine in two of three treatment sessions, and Patient 38 received methylphenidate in two of two treatment sessions (Supplementary Table 3). The data of these patients were kept for further analysis. In 11 (22%) of 50 patients, we identified low residual plasma concentrations of a previous drug in addition to the subsequent drug, indicating incomplete washout of the former (Supplementary Table 4). We observed no adverse events, serious adverse events or suspected unexpected serious adverse reactions related to the study drugs during treatment sessions.

### Primary outcome: effects of stimulants on pupillary responses during pupillometry

**Figure 5:**
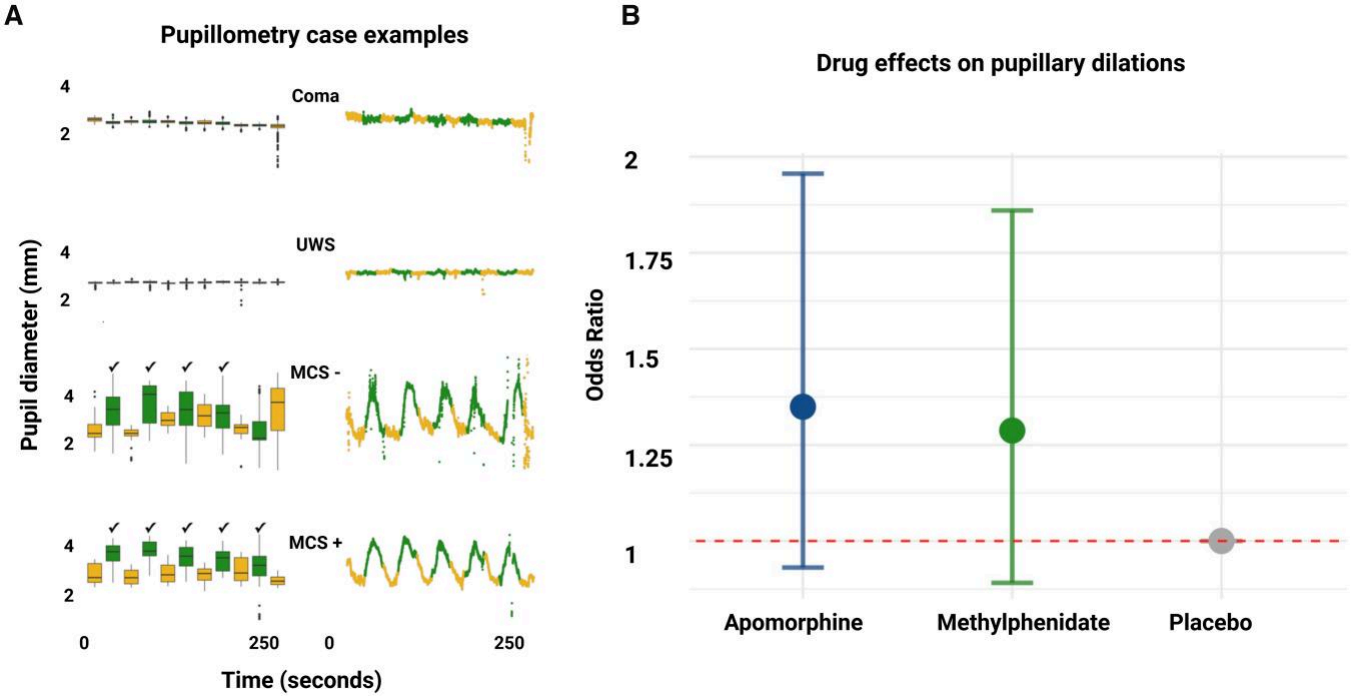
**Automated pupillometry and the effects of stimulant drugs during mental arithmetic.** Representative pupillometry data of four DoC patients at baseline (T0), revealing the count of statistically significant pupillary dilations during mental arithmetic tasks (**A**). The data are graphically represented in box plots (*left*) and scatter plots (*right*). Mental arithmetic = green; rest = yellow; tick symbol (✓) = significant pupillary dilation (*P* < 0.0001). No pupillary dilations were observed in the coma patient and the UWS patient. In contrast, the MCS minus patient and the MCS plus patient responded with pupillary dilations during ≥4 of 5 mental arithmetic tasks. This meant the MCS minus patient (Patient 16 in Fig. 3 and Supplementary Table 5) fulfilled CMD criteria. Stimulants had no statistically significant effects on the number of pupil dilations during mental arithmetic (**B**). CMD = cognitive motor dissociation; DoC = disorders of consciousness; MCS = minimally conscious state.

In total, we obtained 590 pupillometry recordings of sufficient quality during mental arithmetic tasks in 48 patients (i.e. a mean of 12.3 pupillometry recordings per patient; Supplementary Table 5). Recordings from two patients were discarded owing to technical issues. Figure 5A shows representative case examples illustrating the presence or absence of pupillary responses. Setting the threshold for a pupillary response at three dilations per arithmetic set, this was noted during 70 (11.9%) pupillometry recordings. Twenty-five (52.1%) of patients passed this threshold at least once. Four or five pupillary dilations per set were noted during 17 (2.9%) pupillometry recordings. Twelve (25%) patients passed this stricter threshold at least once. Ten (83.3%) of these patients were alive at ICU discharge (Fig. 3), compared to 25 of 36 (69.4%) patients who did not pass the stricter threshold (*P* = 0.47). Seven (14.6%) patients fulfilled criteria for CMD.

In a GLMM analysis of the effects of apomorphine and methylphenidate across different treatment sessions (moderate and hard mental arithmetic tasks combined), there were no differences in pupillary responses compared to placebo for either apomorphine (OR 1.35, 95% CI: 0.93 to 1.96) or methylphenidate (OR 1.29, 95% CI: 0.89 to 1.86) (Fig. 5B). No changes were observed at T15 for either drug (apomorphine: OR 1.21, 95% CI: 0.73 to 2.02; methylphenidate: OR 0.76, 95% CI: 0.45 to 1.28) or at T60 (apomorphine: OR 0.75, 95% CI: 0.45 to 1.24; methylphenidate: OR 0.76, 95% CI: 0.46 to 1.26). Sensitivity analyses based on the GLMM for pupillometry effects of stimulants with covariates sex, sedation and strata (Supplementary material, ‘Results’ section 1) and the GLM (data not shown) did not change these results.

### Secondary outcomes: clinical arousal effects of stimulants

**Figure 6:**
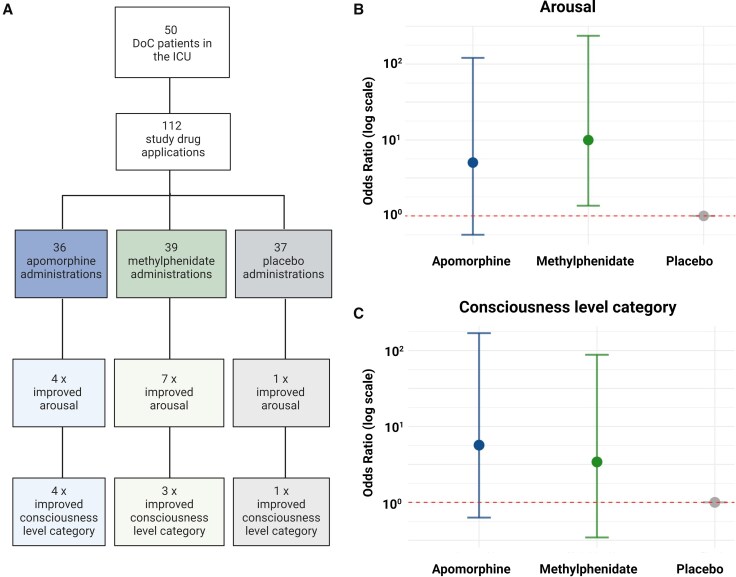
**Clinical effects of stimulant drugs on arousal and consciousness level categories.** (**A**) Fifty DoC patients received 112 administrations of a stimulant drug or placebo. Numerically increased odds ratios for improved arousal (**B**) and for a shift towards a higher consciousness level category (**C**) were seen after administration of methylphenidate and apomorphine. DoC = disorders of consciousness; ICU = intensive care unit.

We assessed the efficacy of apomorphine, methylphenidate and placebo in inducing temporary clinical responses among acute DoC patients. These responses were defined as either temporarily improved arousal without a shift to a higher consciousness category or temporarily improved arousal accompanied by a shift to a higher consciousness category. Clinical data are summarized in Table 2 and Supplementary Table 6, as well as Figs 2, 3 and 6.

**Table 2:** Patients with improved clinical arousal after drug administration

Patient ID	FOUR/SECONDs before drug	DoC before drug	FOUR/SECONDs after drug	DoC after drug	Trial drug	Description of improved arousal response after drug administration
12	8/1	UWS	13/8	eMCS^a^	Methylphenidate	Spontaneous and sustained eye opening. Visual fixation of people at the bedside. Positive mirror test (fixation of one’s own reflection). Functional communication (correctly answering yes or no to 5/5 questions by nodding or shaking the head).
16	11/2	MCS minus	12/6	MCS plus^a^	Apomorphine	Consistent command-following to squeeze right hand (3/3 times), dorsiflex right foot (3/3 times) and to (make attempts to) smile. More lively right extremity movements. Orientation behaviour by reaching for objects with right hand.
18	13/6	MCS plus	14/6	MCS plus	Methylphenidate	Improved visual fixation of people at the bedside. Consistent command-following to squeeze both hands (3/3 times) and dorsiflex left foot (3/3 times). Reproducible tongue protrusion on command (2/3 times).
20^b^	10/4	MCS minus	14/6	MCS plus^a^	Methylphenidate	Consistent and reproducible command-following to squeeze right hand (3/3 times) and dorsiflex right foot (2/3 times).
20	9/1	UWS	14/6	MCS plus^a^	Apomorphine	Spontaneous eye opening. Visual pursuit in a horizontal line. Consistent command-following to smile (3/3 times), to nod or shake head (3/3 times) and bend all four extremities (3/3 times).
21^b^	11/1	UWS	13/6	MCS plus^a^	Apomorphine	Consistent command-following to dorsiflex right foot (3/3 times). Reproducible tongue protrusion (2/3 times).
21	13/6	MCS plus	14/6	MCS plus	Methylphenidate	Spontaneous and sustained eye opening. More lively tongue and right extremity movements.
26	11/5	MCS minus	12/5	MCS minus	Methylphenidate	Sustained visual fixation. Livelier right upper extremity movements.
29	15/4	MCS minus	16/6	MCS plus^a^	Apomorphine	Livelier overall movements and sounds. Consistent command-following to dorsiflex left foot (3/3 times).
34	7/1	UWS	8/7	MCS plus^a^	Placebo	Sustained visual pursuit in a horizontal line. Consistent command-following to open and close eyes (3/3 times) and to blink. (3/3 times). Intentional communication (incorrectly answering yes or no to autobiographical and situational questions by blinking).
43	15/6	MCS plus	16/6	MCS plus	Methylphenidate	Consistent command-following to open and close eyes (3/3 times).
49	10/1	UWS	10/5	MCS minus^a^	Methylphenidate	Contextual crying and laughing during interaction with family members.

DoC = disorders of consciousness; eMCS = emerged from MCS; FOUR = Full Outline of UnResponsiveness; MCS = minimally conscious state; SECONDs = Simplified Evaluation of Consciousness Disorders; UWS = unresponsive wakefulness syndrome.

^a^Improvement within consciousness category after study drug administration.

^b^Patients 20 and 21 improved twice.

### Arousal effects

Of the 50 patients, 10 (20%) showed clinically improved arousal at least once after administration of a study drug (Patients 20 and 21 showed improvement twice; Fig. 2). Improved arousal was seen following seven doses of methylphenidate, four doses of apomorphine and one dose of placebo (Fig. 6). In the GLMM, methylphenidate increased the probability of improved arousal (OR 9.96, 95% CI 1.36 to 235.8). Apomorphine demonstrated a numerically positive effect, but this was not statistically significant (OR 5.04, 95% CI 0.56 to 120.7). Sensitivity analyses based on the GLMM with the covariates age, sex, sedation and strata (Supplementary material, ‘Results’ section 2) and the GLM (data not shown) did not change these results. Nine of the 10 (90%) patients with improved arousal were alive at ICU discharge, compared to 28 of 40 (70%) non-responders (*P* = 0.26).

### Change toward higher consciousness level categories

A change toward a higher consciousness level category occurred after four doses of apomorphine, three doses of methylphenidate and one dose of placebo (Fig. 6). In the GLMM, methylphenidate led to a numerical increase toward a higher consciousness level category compared to placebo, but this effect did not reach statistical significance (OR 3.41, 95% CI 0.34 to 88.00). Similarly, apomorphine was associated with a numerically increased probability of advancing to higher consciousness level categories, but this result lacked statistical significance (OR 5.67, 95% CI 0.63 to 169.46). Sensitivity analyses based on the GLMM after adjustment for the covariates sex, age, sedation and strata (Supplementary material, ‘Results’ section 3) and the GLM (data not shown) confirmed these results.

### *Post hoc* sensitivity analyses of greater pupillary responsiveness, baseline arousal, survival and brain injury types

Of the 12 patients who passed the stricter threshold for pupillary responsiveness (i.e. four or five pupillary dilations during mental arithmetic) at least once, six (50%) had improved arousal after study drug administration (Fig. 3). In comparison, of the 36 patients unable to pass this threshold, only four (11.1%) had improved arousal (*P* = 0.009, Fisher’s exact test).

Furthermore, CMD patients, patients with baseline FOUR scores ≥9, patients with baseline consciousness categories MCS minus or better, patients who survived to ICU discharge, patients with non-anoxic brain injuries and patients with infratentorial lesions all had numerically increased arousal responses, but these results were statistically non-significant (Supplementary material, ‘Results’ section 4). Supplementary Table 7 summarizes the neuroimaging findings of brain injuries.

## Discussion

We performed a randomized trial assessing the effects of two stimulant drugs on consciousness levels and consciousness biomarkers in the ICU. To our knowledge, this is the largest trial of stimulant drugs in the ICU and the first to investigate two different stimulants simultaneously. Contrary to our main hypothesis, we did not find an effect on consciousness biomarkers assessed by pupillometry. We found small, temporary clinical effects of stimulants on arousal, but this finding requires replication. Patients passing the stricter threshold during our mental arithmetic pupillometry paradigm more often showed improved arousal after study drug administration. There were no obvious trial-related adverse events.

### Stimulants trials in the ICU are challenging but feasible

Treatment trials in acute DoC are exceedingly rare and have been restricted to small case reports and case series in subacute DoC and outside the ICU,^11,15,30^ owing to the difficulties of performing systematic pharmacological interventions in critically ill patients with heterogenous brain injuries. To circumvent some of these challenges, we used a 3-fold approach.

First, we tailored the study to the challenges of collecting large numbers of acute DoC patients in the ICU. To decrease the required sample size and investigate multiple drugs simultaneously, we employed a cross-over study design that allowed us to investigate multiple treatments with the participants serving as their own controls, and for data to be collected within a relatively short period. Despite the simplicity of the approach, this had not yet been applied in DoC treatment trials in the ICU.^30^ However, even though we increased our sample size to adjust for 20% study drop-outs, the surplus in participants was too small to compensate for the difficulties encountered during study enrollment, resulting in 38 missing administrations (or 11 missing administrations, when considering the sample size without adjustment for drop-outs). These were mostly caused by ICU transfer to other institutions, spontaneous recovery of consciousness or death. Given the challenging study enrollment, an event-based sample size calculation (i.e. completed treatment schedules), as opposed to a subject-based sample size, might have been preferable.

Second, we confirmed drug exposure by measurement of stimulant drug plasma concentrations at both time points T15 and T60. Mass spectrometry revealed a decline in apomorphine concentrations over time (which was expected), while methylphenidate concentrations remained overall unchanged over 60 min. It is important (as we did) to account for the washout period between drug administrations to avoid interactions between the treatments that might influence the results. We identified incomplete washout of a previous drug in a subset of DoC patients, but the remaining plasma levels were low, and a clinically important carry-over effect seems unlikely. Furthermore, we showed that levels of sedation were unlikely to influence our results.

Third, we usedbedside technology to probe for consciousness biomarkers. Even though pupillary responses were not influenced by the two stimulants, pupillometry can easily be timed with clinical management routines and does not require the patient to be moved outside the ICU environment, as is the case with functional MRI. Contrary to our expectations, clinical outcomes may be at least as sensitive to stimulant effects as consciousness biomarkers, if not more so. Although the study sample was too small to rule out adverse events of apomorphine or methylphenidate, we did not observe any harmful effects. The absence of identified safety signals was not unexpected given the published literature^11,30,31^ and the rapid clearance of both drugs, when administered as a single bolus (approximately 18 h).^32,33^ All this is important information for the design of future pharmacological DoC studies in the ICU.

### Stimulants may have brief arousal effects in acute DoC

There is an urgent need to identify treatment approaches for acute brain injury that go beyond preventing secondary brain damage and help DoC patients regain consciousness directly. We found that 20% of the study participants showed temporary improved arousal after administration of a study drug. A transient shift toward better consciousness level categories, mostly from UWS or MCS minus to MCS plus, occurred after four apomorphine and three methylphenidate administrations and once after placebo. Four more patients improved after receiving methylphenidate without progressing within the hierarchy of consciousness categories. Except for the effects of methylphenidate on arousal, these numbers did not reach statistical significance. However, as stated earlier, patients passing the stricter threshold during pupillometry had more often improved arousal after study drug administration than those without this capability. Furthermore, in exploratory sensitivity analyses, CMD, higher clinical consciousness levels, ICU survival and non-anoxic brain injuries were all associated with numerically (but not statistically) higher numbers of arousal responses. Although these preliminary findings appear promising and biologically plausible, they must be interpreted cautiously until confirmed by adequately powered studies.

Dopaminergic agents have been explored as a potential therapy for chronic DoC, but exactly how these drugs may influence behavioural responses is unclear. According to the mesocircuit model,^34^ damage to the anterior forebrain results in decreased dopaminergic synaptic activity in cerebral areas critical for consciousness. More precisely, medium spiny neurons require continuous background synaptic activity and dopaminergic neuromodulation to inhibit the internal globus pallidus. The lack of inhibition of the internal globus pallidus leads to tonic inhibition of the central thalamus, which in turn may cause prolonged inhibition of the anterior forebrain. The hypothesis is that dopaminergic agonists restore connectivity between these brain regions and increase synaptic activity, ultimately leading to improved arousal and DoC outcomes.^11,30,31^ Hence, apomorphine has been tested off-label in patients with chronic DoC due to its dopaminergic properties and established safety profile. Positive results have been observed in case reports and a small sample study involving patients with subacute to chronic DoC.^12,13^ In one study, eight traumatic brain injury patients with subacute to chronic DoC were treated with subcutaneous infusions of apomorphine for 12–16 h per day, with doses ranging from 2 to 8 mg, for a total of 84 days. All patients showed improvement on the Coma Near-Coma Scale and Disability Rating Scale, with awakening occurring between 24 h and 4 weeks after treatment.^12^ In the same vein, methylphenidate has been evaluated for clinical safety and is known to have a low risk of adverse events.^35,36^ A clinical trial evaluated methylphenidate in patients with severe [Glasgow Coma Scale (GCS) 5–8] and moderate (GCS 9–12) traumatic brain injury.^18^ The study involved 40 patients in each group, randomly assigned to receive either methylphenidate treatment or a placebo. Patients were administered the medication twice daily from the second day of admission until discharge. Methylphenidate decreased the length of ICU stay by 3 days and hospital stay by 4.25 days. Those with moderate injury had a 1.5-day reduction in ICU stay with methylphenidate but no significant difference in hospital stays. However, there was no difference in GCS scores at ICU discharge for those treated with methylphenidate.^18^

In sum, although all these studies are subject to small sample sizes and should be interpreted with caution, the possible clinical effects of the two stimulants we observed in the present trial appear biologically plausible. Even a temporary improvement in arousal with a stimulant drug could be important as it may indicate a higher probability of recovery at an early stage after brain injury. Future studies should also investigate the hypothesis that prolonged administration of these drugs (e.g. twice daily for the entire ICU stay) may lead to sustained, clinically meaningful arousal effects in a subset of acute DoC patients.

### Stimulants and consciousness biomarkers assessed by pupillometry

We recently showed that automated pupillometry combined with mental arithmetic can identify CMD in DoC patients with brain injury in the ICU.^8^ Compared to motor imagery paradigms based on functional MRI^6,37-40^ or EEG,^5,7^ the pupillometry paradigm has advantages, including the possibility for serial bedside measurements, low costs and a computationally less complex analysis algorithm.

Using pupillometry we identified seven (14.6%) DoC patients who fulfilled the criteria for CMD. This is comparable to the rate of CMD reported in functional MRI- and EEG-based studies.^4,5,41^ While we could not identify direct effects of apomorphine or methylphenidate on overall pupillary responsiveness, patients who passed the stricter threshold for pupillary responsiveness more often had improved arousal after a study drug than those who could not, suggesting that greater pupillary responsiveness may predict a positive effect of stimulant drugs.

Compared to our previous study,^8^ pupillary responsiveness was somewhat less frequent in the present trial. Differences in the study design and patient cohorts may explain this discrepancy. First, in the earlier patient cohort, pupillary responsiveness was more often seen in DoC patients with anoxic brain injuries who were underrepresented in the present cohort (24% versus 49%). Future research must establish the impact of brain injury aetiology on CMD in general and on CMD identified with pupillometry in particular. Second, the 3-month mortality rate was higher in the present cohort compared to our previous cohort^8^ (46% versus 32%), suggesting that patients in the present study overall had more severe brain injuries. Finally, there was a small but important change in the methodology between the present and our previous studies.^8,19^ In the latter, we directly spoke to patients when instructing them about mental arithmetic tasks, whereas in the present study, we prerecorded instructions and played them through headphones. There is evidence that instructions given through direct speech (face-to-face) evoke more arousal compared to instructions through prerecorded speech.^42,43^ With prerecorded instructions, the absence of real-time interaction and nonverbal cues may affect compliance and task performance.^42-45^ Pupillometry to detect CMD may therefore work best when instructing patients individually rather than using prerecorded speech.

### Limitations

Several limitations must be kept in mind. Per protocol,^21^ we performed NIRS-EEG to investigate neurovascular coupling as another consciousness biomarker and co-primary outcome in addition to pupillometry. Owing to unforeseen challenges regarding the analysis of neurovascular coupling (i.e. EEG artefacts introduced by the NIRS-optodes), a decision was made to proceed with reporting the trial without these data. Furthermore, we enrolled an acute DoC cohort consisting of patients with very heterogeneous brain injuries. While this reflects real-world ICU populations, the heterogeneity also contributes to a decreased signal-to-noise ratio in terms of the potential effects of pharmacological stimulation in certain types of brain injury compared to others. The present cohort included substantially fewer patients with traumatic brain injuries than our previous ICU DoC cohorts (2% versus 29%)^25,26^ because we used EEG caps to enable NIRS-EEG data collection. In contrast to needle electrodes, which we used previously, caps are incompatible with external ventricular drains frequently used in traumatic brain injury. Moreover, we did not confirm residual consciousness using functional MRI- and/or EEG-based consciousness paradigms, given the substantial challenges in the ICU of employing these technologies.^25,26^ There is no gold standard to measure consciousness,^10^ but these methods have documented CMD in 15%–25% of patients with acute DoC.^5,7^ This is in line with what we found using automated pupillometry here and in our initial paper,^8^ even though the sensitivity of pupillometry to detect CMD remains to be established. Head-to-head studies comparing automated pupillometry with functional MRI- and EEG-based active paradigms should therefore attempt to accurately assess the diagnostic performances of all three methodologies to detect CMD. Also, it should be noted that it is important not to confuse the terms arousal, awareness and consciousness. The latter is typically thought of as being a product of the former two, but we have used these terms somewhat interchangeably here, acknowledging the difficulties of precisely determining whether a beneficial response to a stimulant drug was merely an arousal effect or in addition to a sign of improved awareness. Finally, given the double-blind study design and the logistical challenges in the ICU, we opted for a simple observation for a clinical response by a blinded investigator with experience in evaluating DoC. The concern was that subtle clinical signs of arousal may fluctuate and that they would not necessarily be captured in the noisy ICU environment by a rating scale used at a single, prespecified time point. We therefore performed the SECONDs and the FOUR prior to studying drug administration and repeated the scores at the time point when the investigator observed a clinical response. This approach was deliberately pragmatic. We acknowledge that it was biased insofar as we only searched for a positive response, and we could have missed possible detrimental clinical responses, i.e. a paradoxical worsening after stimulant administration, as well as possible additional positive effects.

## Conclusions and future directions

We conducted a randomized, placebo-controlled, double-blind trial of apomorphine and methylphenidate in acute DoC. We identified no safety issues, and we confirmed that CMD is frequent in the ICU. Stimulants had no statistically significant effects on consciousness biomarkers assessed by pupillometry, but we observed temporary increased arousal in some DoC patients, particularly in those with greater pupillary responsiveness. This requires replication. Given the logistical challenges related to study enrollment, an event-based sample size calculation in terms of completed treatment schedules might have resulted in greater power. The present study provides important feasibility data to guide future pharmacological trials in the ICU aimed at improving consciousness recovery after brain injury. For example, future studies could investigate whether repeated administrations of stimulant drugs may lead to prolonged arousal and improved recovery of consciousness. This would have important impacts on ICU management, prognostication and rehabilitation of DoC patients with brain injury. We suggest that these patients may have cerebral reserves that can be activated in the ICU for prognostic and rehabilitation purposes.

## Supplementary Material

awaf228_Supplementary_Data

## Data Availability

Anonymized raw data are available on request. The code used for the pupillometry paradigm is available at https://github.com/lilleoel/clintools.
