# Supplementary material for: Stimulants for disorders of consciousness in the intensive care unit: a randomized, placebo-controlled trial
Source: Brain. 2025 Jun 12;148(10):3523–36. doi: 10.1093/brain/awaf228 (PMC12493042; doi:10.1093/brain/awaf228)
Supplement: awaf228_Supplementary_Data [file awaf228_supplementary_data.pdf]

SUPPLEMENTAL METHODS & RESULTS

CONTENT:

A. SUPPLEMENTAL METHODS

- Methods S1-S5
- Tables S1

B. SUPPLEMENTAL RESULTS

- Results S1-S4
- Tables S2-S7

SUPPLEMENTAL METHODS

METHODS S1

Sedation levels

Levels of sedation were graded as described earlier (references 26 and 27 in main text): ‘None or minimal’, indicating no intravenous fentanyl, remifentanil, propofol, midazolam, sodium thiopental or sevoflurane; ‘low to moderate’, indicating fentanyl < 500 µg/h or < 200 µg/h combined with propofol, remifentanil < 1000 µg/h or < 250 µg/h combined with propofol, propofol < 100 mg/h, midazolam < 10 mg/h, sevoflurane < 3%; and ‘high or very high’, indicating propofol ≥ 100 mg/h, fentanyl ≥ 500 µg/h or ≥ 200 µg/h combined with propofol, remifentanil ≥ 1000 µg/h or ≥ 250 µg/h combined with propofol, midazolam ≥ 10 mg/h, sevoflurane ≥ 3% or any dosage of sodium thiopental.

METHODS S2

Determinations of apomorphine and methylphenidate in human plasma

To correlate the clinical effects, pupillary responses, and EEG measures with the actual drug levels in the blood, samples of 8 mL blood were drawn at T15 and T60. Due to the rapid autooxidation of apomorphine, we collected blood samples in prechilled vacutainers containing K2EDTA and sodium fluoride as anticoagulants. Immediately after the blood was drawn, we added a 0.15 mL solution of 6% ascorbic acid. The samples were then centrifuged and stored at −70°C in a research biobank before analysis using ultra-high-performance liquid chromatography–tandem mass spectrometry as described in the following.

Apomorphine and methylphenidate were purchased from Chiron AS (Trondheim, Norway) and the internal standards 6-acethylmorphine-d6 and methylphenidate-d9 were obtained from Cerilliant (RoundRock, Texas) and QMX Laboratories (Thaxted, UK), respectively. A stock solution of 1.0 mg/mL methylphenidate was prepared in methanol and a stock solution of 0.1 mg/mL apomorphine was prepared in 0.1% ascorbic acid in methanol. A standard solution series with both methylphenidate and apomorphine was freshly prepared in 50% methanol in water with a final content of 0.18% ascorbic acid. Calibration curves were prepared in drug-free plasma ranging from 0.05 to 100 µg/kg for apomorphine and 0.5 to 500 µg/kg for methylphenidate. The method accuracy was 83% with a precision of 8% for apomorphine and 94% with a precision of 4% for methylphenidate (precision was estimated as coefficient of variation).

Plasma samples (100 mg) and fortified calibrators were spiked with 20 µL internal standards and protein precipitated with acetonitrile (700 µL) using a fully automated robotic system. After vigorous shaking for 1 min and 10 min of centrifugation at 1000 g, 750 µL of the supernatant was transferred to a new deep-well plate and acidified with 50 µL 10% formic acid in acetonitrile. The supernatant was evaporated to dryness under a gentle flow of nitrogen heated to 40°C, and the dried eluate was then reconstituted in 100 µL 12.5% methanol:12.5% acetonitrile in 0.05% formic acid.

The analysis was performed by ultra-high-performance liquid chromatography–tandem mass spectrometry using an ACQUITY I-Class UPLC coupled to a XEVO TQS instrument from Waters. The separation of analytes was performed on a Kinetex biphenyl column (2.6 µm, 2.1 x 50 mm) using gradient elution with mobile phase A: 1 mM ammonium formate in 0.1% formic acid and B: 1:1 methanol:acetonitrile with 0.1% formic acid. The gradient program wasas follows using a flow rate of 0.50 mL/min and a column temperature of 50°C: The initial composition of 95% A and 5% B was held for 0.3 min before the mobile phase B was linearly increased to 80% within 2.2 min. Then, the mobile phase B was increased to 100% within 0.2 min and held for 0.80 min before returning to the initial composition. The total run time was 3.9 min, and the injection volume was 0.4 µL.

The mass spectrometer was operated in positive electrospray ionization mode (ESI +) using the following source conditions: capillary voltage at 3.0 kV, source temperature at 150°C, cone gas flow at 150 L/h, desolvation temperature at 600°C and desolvation gas flow at 1000 L/h. The gain was set to 0.5. Argon was used as collision gas, and the pressure in the collision cell was maintained at approximately $4.3 \times 10{-}3$ mbar, corresponding to a flow of 0.15 mL/minute. The quantification was performed by multiple reaction monitoring (MRM) with two transitions for each analyte and one transition for each internal standard. For apomorphine, the MRM two transitions were *m/z* 268.1 -> 237.1 .1 and *m/z* 268.1 -> 219.1 using collision energies (CE) of 18 eV and 20 eV, respectively. The MRM transition used for the internal standard, 6-acethylmorphine-d6, was *m/z* 334.2 -> 165.1 using a CE of 36 eV. For methylphenidate the MRM two transitions were *m/z* 234.1 -> 56.1 and *m/z* 234.1 -> 84.1 using CEs of 19 eV and 36 eV, respectively. The MRM transition used for the internal standard, methylphenidate-d9, was *m/z* 243.2 -> 93.1 using a CE of 19 eV. The cone was held at 20 V for all analytes and internal standards.

METHODS S3

Automated pupillometry, cognitive paradigms, and valuation of pupil dilations

Automated pupillometry is a low-cost, point-of-care bedside technology that can detect cognitive load by demonstrating pupillary dilations in subjects exposed to arousal or mental activity. We have recently demonstrated the possibility of detecting covert consciousness, including cognitive motor dissociation, in ICU patients with acute brain injuries using a convenient paradigm based on automated pupillometry combined with mental arithmetic. For example, in an awake volunteer who understands instructions, pupils will dilate each time the participant engages in mental arithmetic (regardless of whether the calculation is mathematically correct or not), followed by pupillary constriction when told to relax. If this cycle is successfully repeated, say, five times, the individual has demonstrated that he or she can follow commands (and hence is awake and aware) without the need for any verbal or skeletal muscle motor output. Importantly, it is the mental effort and cognitive load that induces pupillary dilation, regardless of whether the result of the calculation is mathematically correct or not. In the present trial, we also assessed pupillary responses to active cognitive paradigms based on mental arithmetic tasks.

To evaluate pupil dilation, we uploaded the data as an Excel file via the device's Bluetooth functionality. We analyzed pupil dilations using an in-house-developed R package which we have made available free of charge online (see “Data sharing and code availability”). Pupillary dilation was defined as a significantly larger pupil during mental arithmetic compared to rest before and aftera stimulus/task (i.e., we calculated the average pupil size during a stimulus and used an unpaired Student's t-test to compare it to the average sizes during the rest periods before and after). Trigger markers of the applied stimuli were inserted manually during recordings. All recordings were visually inspected for artifacts. Data points with abrupt deviations exceeding 1.5 mm from the preceding data point (i.e., eye blinks) were labeled as physiologically implausible and removed. Although these were autocorrelated measurements, we used the unpaired t-test because it is more conservative and does not require the same number of measurements in the groups.

METHODS S4

R code and plots for pupillometry effects of stimulants

CLMM for pupillometry effects of stimulants

#Good with a clear memory before starting

```
rm(list = ls())
```

Load packages

```
library(vctrs)
library(tidyr)
library(dplyr)

##
## Attaching package: 'dplyr'

## The following object is masked from 'package:vctrs':
##
##     data_frame

## The following objects are masked from 'package:stats':
##
##     filter, lag

## The following objects are masked from 'package:base':
##
##     intersect, setdiff, setequal, union

library(readxl)
library(ordinal)

##
## Attaching package: 'ordinal'

## The following object is masked from 'package:dplyr':
##
##     slice

library(ggplot2)
```

#Load Data

```
data <- read_excel("~/Desktop/Ph.D./Ph.D./PhD Projekter/CONMED3/Manuscript Wo
rk/Pupillometri CONMED/Dilations CLMM.xlsx")
```

Reshape the data to long format

```
data_long <- pivot_longer(
  data,
  cols = -c(Subject, Drug, Attempt, Consciousness, age, sex, sedation, strata
```

```
), # Excluding identifier columns
  names_to = c("Test", "Session"),
  names_pattern = "(Test \\d) (\\w+)",
  values_to = "Score"
)
```

Convert Session, Test, and Consciousness to factor with correct levels

```
data_long$Session <- factor(data_long$Session, levels = c("Baseline", "T15",
"T60"))
data_long$Test <- factor(data_long$Test, levels = c("Test 1", "Test 2", "Test
3"))
data_long$Consciousness <- factor(data_long$Consciousness, levels = c("Coma",
"UWS", "MCS minus", "MCS plus"))
data_long$Drug <- factor(data_long$Drug)
data_long$Score <- factor(data_long$Score)
```

Subset data by test and check

```
data_test2 <- filter(data_long, Test == "Test 2")
data_test3 <- filter(data_long, Test == "Test 3")
```

Ensure that Score is numeric for addition

```
data_test2$Score <- as.numeric(as.character(data_test2$Score))
data_test3$Score <- as.numeric(as.character(data_test3$Score))
```

Combine and sum the scores from Test 2 and Test 3

```
combined_test_sum <- bind_rows(data_test2, data_test3) %>%
  group_by(Subject, Drug, Session) %>%
  summarize(
    Score = sum(Score, na.rm = TRUE),
    .groups = 'drop'
  )
```

Ensure Score is treated as a factor for ordinal regression

```
combined_test_sum$Score <- factor(combined_test_sum$Score)
```

Relevel Drug to make Placebo the reference category

```
combined_test_sum$Drug <- relevel(combined_test_sum$Drug, ref = "placebo")
```

Function to check convergence for a clmm model

```
check_convergence <- function(model) {
  if (is.null(model$convergence)) {
    print("Model converged successfully.")
  } else {
    print("Model did not converge. Check for issues with the model fitting.")
  }

  max_gradient <- model$details$maxGrad
  n_iter <- model$niter

  if (!is.null(n_iter) && length(n_iter) > 0) {
    cat("Number of iterations:", n_iter[1], "\n")
  } else {
    print("Number of iterations not available.")
  }

  if (!is.null(max_gradient) && length(max_gradient) > 0) {
    cat("Max gradient:", max_gradient, "\n")
    if (max_gradient > 1e-4) {
      print("Warning: Model might not have fully converged. High max gradient 
detected.")
    }
  } else {
    print("Max gradient not available.")
  }
}
```

#FITTING CLMM FOR COMBINED TESTS#

Fitting the CLMM for combined Test 2 and Test 3

```
clmm_model_combined <- clmm(Score ~ Drug * Session + (1 | Subject),
                            data = combined_test_sum,
                            link = "logit")
```

Displaying the summary of the model

```
summary_clmm_combined <- summary(clmm_model_combined)
print(summary_clmm_combined)

## Cumulative Link Mixed Model fitted with the Laplace approximation
##
## formula: Score ~ Drug * Session + (1 | Subject)
## data:    combined_test_sum
##
##  link  threshold nobs logLik  AIC     niter      max.grad cond.H
##  logit flexible  300  -548.03 1130.06 1618(3257) 5.42e-04 1.0e+03
##
```

```
## Random effects:
##  Groups  Name        Variance Std.Dev.
##  Subject (Intercept) 0.03051  0.1747  
## Number of groups:  Subject 49 
## 
## Coefficients:
##                                  Estimate Std. Error z value Pr(>|z|)  
## Drugapomorphine                    0.7476     0.4363   1.713   0.0866 .
## Drugmethylphenidate                0.6243     0.4238   1.473   0.1408  
## SessionT15                         0.1004     0.4214   0.238   0.8118  
## SessionT60                         0.6704     0.4258   1.574   0.1154  
## Drugapomorphine:SessionT15         0.5249     0.6256   0.839   0.4014  
## Drugmethylphenidate:SessionT15   -0.5672     0.5959  -0.952   0.3411  
## Drugapomorphine:SessionT60       -0.8502     0.6224  -1.366   0.1719  
## Drugmethylphenidate:SessionT60   -0.6707     0.5993  -1.119   0.2631  
## ---
## Signif. codes:  0 '***' 0.001 '**' 0.01 '*' 0.05 '.' 0.1 ' ' 1
## 
## Threshold coefficients:
##      Estimate Std. Error z value
## 0|1   -1.4200     0.3269  -4.343
## 1|2   -0.2752     0.3074  -0.895
## 2|3    0.8447     0.3103   2.722
## 3|4    1.5802     0.3184   4.963
## 4|5    2.6131     0.3453   7.568
## 5|6    3.8822     0.4298   9.033
## 6|8    5.6283     0.7717   7.293
## 8|12   6.3250     1.0467   6.043
```

```
check_convergence(clmm_model_combined)
```

```
## [1] "Model converged successfully."
## [1] "Number of iterations not available."
## [1] "Max gradient not available."
```

Extract coefficients

```
coefficients <- coef(clmm_model_combined)
```

Calculate Odds Ratios

```
odds_ratios <- exp(coefficients)
```

Calculate standard errors and confidence intervals

```
conf_int <- confint(clmm_model_combined)  # Default is 95% CI
odds_ratio_ci <- exp(conf_int)
```

Print the results

```
odds_ratios

##                          0|1                          1|2 
##                    0.2417039                    0.7594432 
##                          2|3                          3|4 
##                    2.3272690                    4.8561038 
##                          4|5                          5|6 
##                   13.6407575                   48.5291599 
##                          6|8                         8|12 
##                  278.1981469                  558.3579829 
##               Drugapomorphine          Drugmethylphenidate 
##                    2.1118360                    1.8668923 
##                    SessionT15                   SessionT60 
##                    1.1055716                    1.9550523 
##     Drugapomorphine:SessionT15 Drugmethylphenidate:SessionT15 
##                    1.6903707                    0.5670925 
##     Drugapomorphine:SessionT60 Drugmethylphenidate:SessionT60 
##                    0.4273121                    0.5113367

odds_ratio_ci

##                                      2.5 %       97.5 %
## 0|1                              0.1273481    0.4587486
## 1|2                              0.4157495    1.3872634
## 2|3                              1.2669040    4.2751316
## 3|4                              2.6016241    9.0642393
## 4|5                              6.9331324   26.8378356
## 5|6                             20.9019293  112.6728223
## 6|8                             61.3020800 1262.5054313
## 8|12                            71.7673045 4344.0901022
## Drugapomorphine                  0.8980411    4.9661994
## Drugmethylphenidate              0.8134848    4.2843909
## SessionT15                       0.4840340    2.5252125
## SessionT60                       0.8486008    4.5041552
## Drugapomorphine:SessionT15       0.4959390    5.7615011
## Drugmethylphenidate:SessionT15   0.1763724    1.8233798
## Drugapomorphine:SessionT60       0.1261716    1.4472014
## Drugmethylphenidate:SessionT60   0.1579576    1.6552877
```

Combine odds ratios and their confidence intervals into a data frame

```
results <- data.frame(
  Odds_Ratios = odds_ratios,
  CI_Lower = odds_ratio_ci[, 1],
  CI_Upper = odds_ratio_ci[, 2]
)
print(results)
```

```
##                                  Odds_Ratios   CI_Lower     CI_Upper
## 0|1                                0.2417039  0.1273481    0.4587486
## 1|2                                0.7594432  0.4157495    1.3872634
## 2|3                                2.3272690  1.2669040    4.2751316
## 3|4                                4.8561038  2.6016241    9.0642393
## 4|5                               13.6407575  6.9331324   26.8378356
## 5|6                               48.5291599 20.9019293  112.6728223
## 6|8                              278.1981469 61.3020800 1262.5054313
## 8|12                             558.3579829 71.7673045 4344.0901022
## Drugapomorphine                    2.1118360  0.8980411    4.9661994
## Drugmethylphenidate                1.8668923  0.8134848    4.2843909
## SessionT15                         1.1055716  0.4840340    2.5252125
## SessionT60                         1.9550523  0.8486008    4.5041552
## Drugapomorphine:SessionT15         1.6903707  0.4959390    5.7615011
## Drugmethylphenidate:SessionT15     0.5670925  0.1763724    1.8233798
## Drugapomorphine:SessionT60         0.4273121  0.1261716    1.4472014
## Drugmethylphenidate:SessionT60     0.5113367  0.1579576    1.6552877
```

Simpler model

Fit the CLM without random effects for sensitivity analysis

```
clm_model <- clm(Score ~ Drug * Session,
                 data = combined_test_sum,
                 link = "logit")  # or "probit", depending on your previous m
odel's link function
```

Summary of the CLM

```
summary(clm_model)
```

```
## formula: Score ~ Drug * Session
## data:    combined_test_sum
## 
##  link  threshold nobs logLik  AIC     niter max.grad cond.H
## logit flexible  300  -548.06 1128.13 6(0)  2.39e-08 5.3e+02
## 
## Coefficients:
##                                  Estimate Std. Error z value Pr(>|z|)
## Drugapomorphine                    0.7485     0.4351   1.720   0.0854 .
## Drugmethylphenidate                0.6319     0.4214   1.500   0.1337
## SessionT15                         0.1012     0.4207   0.241   0.8098
## SessionT60                         0.6640     0.4245   1.564   0.1178
## Drugapomorphine:SessionT15         0.5169     0.6246   0.828   0.4079
## Drugmethylphenidate:SessionT15    -0.5623     0.5944  -0.946   0.3442
## Drugapomorphine:SessionT60        -0.8428     0.6215  -1.356   0.1751
## Drugmethylphenidate:SessionT60    -0.6590     0.5966  -1.105   0.2693
## ---
```

```
## Signif. codes:  0 '***' 0.001 '**' 0.01 '*' 0.05 '.' 0.1 ' ' 1
##
## Threshold coefficients:
##      Estimate Std. Error z value
## 0|1   -1.4040     0.3190  -4.401
## 1|2   -0.2645     0.3026  -0.874
## 2|3    0.8483     0.3080   2.755
## 3|4    1.5794     0.3165   4.991
## 4|5    2.6062     0.3424   7.611
## 5|6    3.8703     0.4257   9.091
## 6|8    5.6142     0.7688   7.302
## 8|12   6.3107     1.0446   6.041
```

Check proportional odds assumption with nominal_test() for the CLM

```
nom_test_clm <- nominal_test(clm_model)
print(nom_test_clm)
```

```
## Tests of nominal effects
##
## formula: Score ~ Drug * Session
##              Df  logLik    AIC LRT Pr(>Chi)
## <none>          -548.06 1128.1
##  Drug
## Session
## Drug:Session
```

Check Multicollinearity with VIF

```
lm_model <- lm(as.numeric(Score) ~ Drug + Session, data = combined_test_sum)
vif_results <- car::vif(lm_model)
print(vif_results)
```

```
##         GVIF Df GVIF^(1/(2*Df))
##  Drug      1  2               1
## Session    1  2               1
```

Check Normality of Random Effects

Extract random effects and plot Q-Q plot

```
ranef_combined <- ranef(clmm_model_combined)$Subject[[1]] # Extract the rand
om effects for subjects
qqnorm(ranef_combined, main = "Q-Q Plot of Random Effects")
qqline(ranef_combined)
```

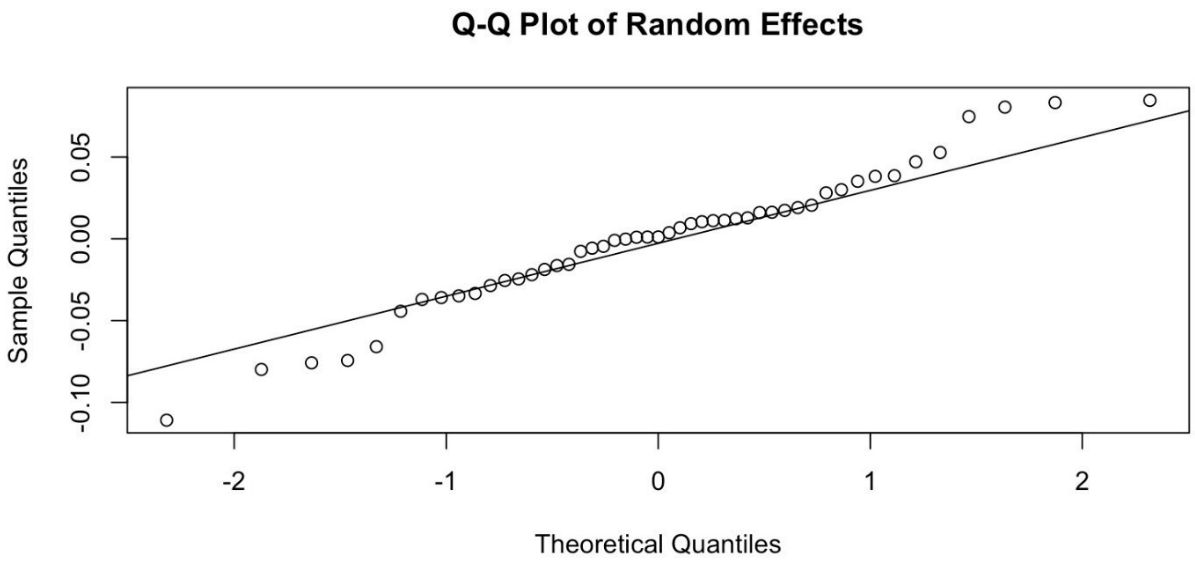

Creating a data frame for plotting based on calculated results

```
drug_effects <- data.frame(
  Drug = c("Placebo", "Apomorphine", "Methylphenidate"),
  Odds_Ratios = c(1, 2.1118360, 1.8668923),
  CI_Lower = c(1, 0.8980411, 0.8134848),
  CI_Upper = c(1, 4.9661994, 4.2843909)
)
```

Define the colors for each drug, aligned with the new order

```
drug_colors <- c("Placebo" = "darkgrey", "Apomorphine" = "dodgerblue4",
                 "Methylphenidate" = "forestgreen")
```

Plot the overall effects compared to placebo

```
odds_ratio_plot <- ggplot(drug_effects, aes(x = Drug, y = Odds_Ratios, color
= Drug)) +
  geom_point(size = 5, alpha = 1) +  # Plot points
  geom_errorbar(aes(ymin = CI_Lower, ymax = CI_Upper), width = 0.2, size = 1,
alpha = 0.8) +  # Error bars with horizontal caps
  geom_hline(yintercept = 1, linetype = "dashed", color = "red") +
  labs(title = "Pupillary Dilations",
       x = NULL,
       y = "Odds Ratio") +
  scale_color_manual(values = drug_colors) +  # Set manual colors for drugs
  theme_minimal() +
  theme(
    plot.title = element_text(hjust = 0.5),
```

```
    legend.title = element_blank(),
    axis.text.x = element_text(angle = 45, hjust = 1) # Tilt the x-axis labe
ls for readability
  )
```

```
## Warning: Using `size` aesthetic for lines was deprecated in ggplot2 3.4.0.
## ℹ Please use `linewidth` instead.
## This warning is displayed once every 8 hours.
## Call `lifecycle::last_lifecycle_warnings()` to see where this warning was
## generated.
```

```
# Show the plot
print(odds_ratio_plot)
```

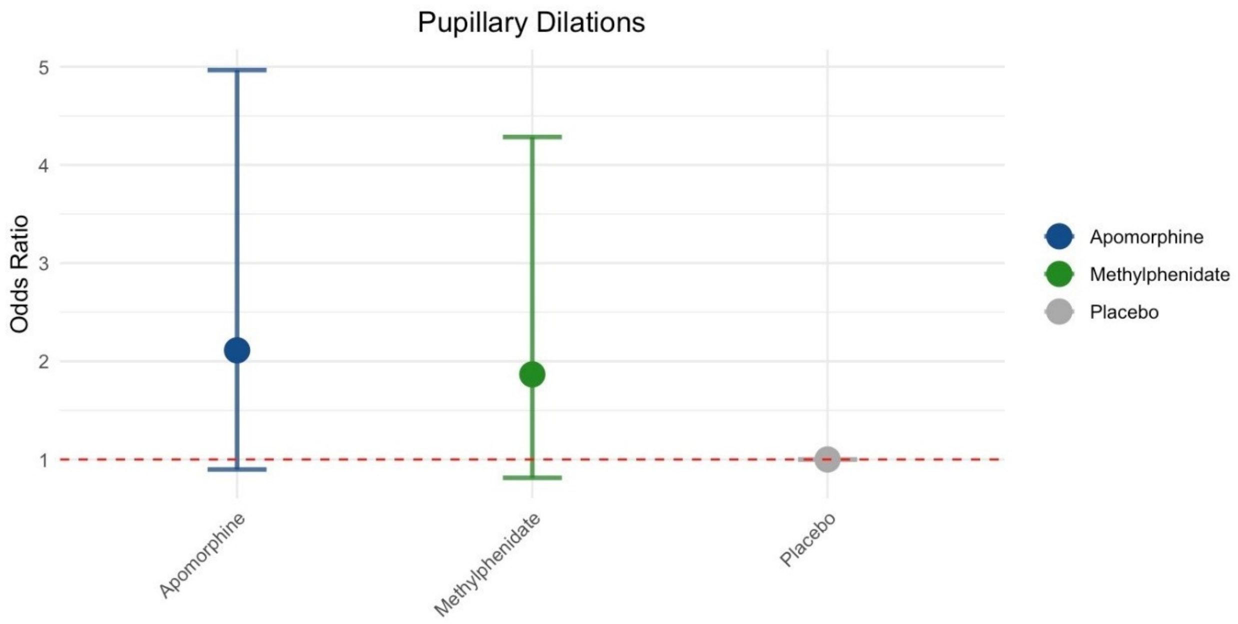

ANOVA-like comparisons to check for interaction effects

```
model_wo_interaction <- clmm(Score ~ Drug + Session + (1 | Subject),
                             data = combined_test_sum,
                             link = "logit")
anova_result <- anova(clmm_model_combined, model_wo_interaction)
print(anova_result) # Check for interaction significance
```

```
## Likelihood ratio tests of cumulative link models:
##
##                      formula:                                link: threshol
d:
## model_wo_interaction Score ~ Drug + Session + (1 | Subject) logit flexible
## clmm_model_combined  Score ~ Drug * Session + (1 | Subject) logit flexible
##
##                      no.par    AIC  logLik LR.stat df Pr(>Chisq)
```

```
## model_wo_interaction      13 1129.1 -551.55
## clmm_model_combined       17 1130.1 -548.03  7.0435  4      0.1336
```

Test main effects

```
model_wo_Drug <- clmm(Score ~ Drug + (1 | Subject), data = combined_test_sum,
link = "logit")
anova_result <- anova(clmm_model_combined, model_wo_Drug)
print(anova_result) # Main effect of Drug - not significant

## Likelihood ratio tests of cumulative link models:
##
##                     formula:                              link: threshold
:
## model_wo_Drug       Score ~ Drug + (1 | Subject)           logit flexible
## clmm_model_combined Score ~ Drug * Session + (1 | Subject) logit flexible
##
##                     no.par  AIC  logLik LR.stat df Pr(>Chisq)##
model_wo_Drug               11 1125.6 -551.82
## clmm_model_combined     17 1130.1 -548.03  7.5835  6     0.2702

model_wo_Session <- clmm(Score ~ Session + (1 | Subject), data = combined_tes
t_sum, link = "logit")
anova_result <- anova(clmm_model_combined, model_wo_Session)
print(anova_result) # Main effect of Session - not significant

## Likelihood ratio tests of cumulative link models:
##
##                     formula:                              link: threshold
:
## model_wo_Session    Score ~ Session + (1 | Subject)      logit flexible##
clmm_model_combined Score ~ Drug * Session + (1 | Subject) logit flexible##
##                     no.par    AIC logLik LR.stat df Pr(>Chisq)
## model_wo_Session        11 1131.1 -554.56
## clmm_model_combined     17 1130.1 -548.03  13.054  6    0.04219 *
## ---
## Signif. codes:  0 '***' 0.001 '**' 0.01 '*' 0.05 '.' 0.1 ' ' 1
```

CLMM for pupillometry effects of stimulants (with covariates)

Good with a clear memory before starting

```
rm(list = ls())
```

Load packages

```
library(vctrs)
library(tidyr)
library(dplyr)

##
## Attaching package: 'dplyr'

## The following object is masked from 'package:vctrs':
##
##     data_frame

## The following objects are masked from 'package:stats':
##
##     filter, lag

## The following objects are masked from 'package:base':
##
##     intersect, setdiff, setequal, union

library(readxl)
library(ordinal)

##
## Attaching package: 'ordinal'

## The following object is masked from 'package:dplyr':
##
##     slice

library(ggplot2)
```

#Load Data

```
data <- read_excel("~/Desktop/Ph.D./Ph.D./PhD Projekter/CONMED3/Manuscript Wo
rk/Pupillometri CONMED/Dilations CLMM.xlsx")
```

Reshape the data to long format

```
data_long <- pivot_longer(
  data,
  cols = -c(Subject, Drug, Attempt, Consciousness, age, sex, sedation, strata
```

```
), # Excluding identifier columns
  names_to = c("Test", "Session"),
  names_pattern = "(Test \\d) (\\w+)",
  values_to = "Score"
)
```

Convert Session, Test, and Consciousness to factor with correct levels

```
data_long$Session <- factor(data_long$Session, levels = c("Baseline", "T15",
"T60"))
data_long$Test <- factor(data_long$Test, levels = c("Test 1", "Test 2", "Test
3"))
data_long$Consciousness <- factor(data_long$Consciousness, levels = c("Coma",
"UWS", "MCS minus", "MCS plus"))
data_long$Drug <- factor(data_long$Drug)
data_long$Score <- factor(data_long$Score)
```

Subset data by test and check

```
data_test2 <- filter(data_long, Test == "Test 2")
data_test3 <- filter(data_long, Test == "Test 3")
```

Ensure that Score is numeric for addition

```
data_test2$Score <- as.numeric(as.character(data_test2$Score))
data_test3$Score <- as.numeric(as.character(data_test3$Score))
```

Combine and sum the scores from Test 2 and Test 3

```
combined_test_sum <- bind_rows(data_test2, data_test3) %>%
  group_by(Subject, Drug, Session, age, sex, sedation, strata) %>%
  summarize(
    Score = sum(Score, na.rm = TRUE),
    .groups = 'drop'
  )
```

Ensure Score is treated as a factor for ordinal regression

```
combined_test_sum$Score <- factor(combined_test_sum$Score)
```

Relevel Drug to make Placebo the reference category

```
combined_test_sum$Drug <- relevel(combined_test_sum$Drug, ref = "placebo")
```

Function to check convergence for a clmm model

```
check_convergence <- function(model) {
  if (is.null(model$convergence)) {
    print("Model converged successfully.")
  } else {
    print("Model did not converge. Check for issues with the model fitting.")
  }

  max_gradient <- model$details$maxGrad
  n_iter <- model$niter

  if (!is.null(n_iter) && length(n_iter) > 0) {
    cat("Number of iterations:", n_iter[1], "\n")
  } else {
    print("Number of iterations not available.")
  }

  if (!is.null(max_gradient) && length(max_gradient) > 0) {
    cat("Max gradient:", max_gradient, "\n")
    if (max_gradient > 1e-4) {
      print("Warning: Model might not have fully converged. High max gradient
detected.")
    }
  } else {
    print("Max gradient not available.")
  }
}
```

#FITTING CLMM FOR COMBINED TESTS#

Fitting the CLMM with covariates

```
clmm_model_combined <- clmm(Score ~ Drug * Session + age + sex + sedation + s
trata + (1 | Subject),
                            data = combined_test_sum,
                            link = "logit")
```

Fitting the CLMM without covariates

```
clmm_model_simple <- clmm(Score ~ Drug * Session + (1 | Subject),
                          data = combined_test_sum,
                          link = "logit")
```

Compare the models using ANOVA

```
anova(clmm_model_simple, clmm_model_combined, test = "Chisq")

## 'test' argument ignored in anova.clm
```

```
## Likelihood ratio tests of cumulative link models:
##
##                     formula:
## clmm_model_simple   Score ~ Drug * Session + (1 | Subject)
## clmm_model_combined Score ~ Drug * Session + age + sex + sedation + strata
+ (1 | Subject)
##                     link: threshold:
##  clmm_model_simple  logit  flexible
## clmm_model_combined logit flexible
##
##                     no.par    AIC  logLik LR.stat df Pr(>Chisq)
## clmm_model_simple       17 1143.0 -554.50
## clmm_model_combined     21 1147.7 -552.85  3.3052  4     0.5081
```

Displaying the summary of the model

```
summary_clmm_combined <- summary(clmm_model_combined)
print(summary_clmm_combined)
```

```
## Cumulative Link Mixed Model fitted with the Laplace approximation
##
## formula: Score ~ Drug * Session + age + sex + sedation + strata + (1 |
##     Subject)
## data:    combined_test_sum
##
##  link  threshold nobs logLik  AIC     niter      max.grad cond.H
##  logit flexible  303  -552.85 1147.70 2645(3438) 1.77e-03 5.9e+09
##
## Random effects:
##  Groups  Name        Variance  Std.Dev.
##  Subject (Intercept) 1.348e-08 0.0001161
## Number of groups:  Subject 49
##
## Coefficients:
##                                Estimate Std. Error z value Pr(>|z|)
## Drugapomorphine                0.625270   0.428347   1.460    0.144
## Drugmethylphenidate            0.557556   0.422840   1.319    0.187
## SessionT15                     0.109938   0.421398   0.261    0.794
## SessionT60                     0.647543   0.423482   1.529    0.126
## age                            0.006436   0.011452   0.562    0.574
## sexmale                        0.343407   0.239129   1.436    0.151
## sedationyes                   -0.247483   0.297996  -0.830    0.406
## strata≥MCS                    -0.139228   0.215175  -0.647    0.518
## Drugapomorphine:SessionT15     0.538394   0.621586   0.866    0.386
## Drugmethylphenidate:SessionT15 -0.571372   0.594420  -0.961    0.336
## Drugapomorphine:SessionT60     -0.658901   0.613553  -1.074    0.283
## Drugmethylphenidate:SessionT60 -0.654304   0.596384  -1.097    0.273
##
## Threshold coefficients:
##     Estimate Std. Error z value
```

```
## 0|1  -0.8688     0.8246  -1.054
## 1|2   0.2713     0.8202   0.331
## 2|3   1.3781     0.8242   1.672
## 3|4   2.1044     0.8281   2.541
## 4|5   3.1504     0.8394   3.753
## 5|6   4.4183     0.8779   5.033
## 6|7   6.1630     1.0878   5.665
## 7|8   6.8597     1.2977   5.286
```

```
check_convergence(clmm_model_combined)
```

```
## [1] "Model converged successfully."
## [1] "Number of iterations not available."
## [1] "Max gradient not available."
```

Extract coefficients

```
coefficients <- coef(clmm_model_combined)
```

Calculate Odds Ratios

```
odds_ratios <- exp(coefficients)
```

Calculate standard errors and confidence intervals

```
conf_int <- confint(clmm_model_combined)  # Default is 95% CI
odds_ratio_ci <- exp(conf_int)
```

Print the results

```
odds_ratios
```

```
##                          0|1                         1|2
##                    0.4194345                   1.3116830
##                          2|3                         3|4
##                    3.9675118                   8.2023831
##                          4|5                         5|6
##                   23.3454450                  82.9524649
##                          6|7                         7|8
##                  474.8301158                 953.0806792
##               Drugapomorphine          Drugmethylphenidate
##                    1.8687511                   1.7463996
##                   SessionT15                  SessionT60
##                    1.1162092                   1.9108399
##                          age                     sexmale
##                    1.0064563                   1.4097429
##                  sedationyes                 strata≥MCS
##                    0.7807632                   0.8700301
##       Drugapomorphine:SessionT15 Drugmethylphenidate:SessionT15
```

```
##                      1.7132525                        0.5647498
##     Drugapomorphine:SessionT60 Drugmethylphenidate:SessionT60
##                      0.5174197                        0.5198038
```

```r
odds_ratio_ci
```

```
##                                     2.5 %       97.5 %
## 0|1                            0.08331459     2.111579
## 1|2                            0.26284965     6.545613
## 2|3                            0.78884812    19.954601
## 3|4                            1.61845569    41.569930
## 4|5                            4.50537119   120.968901
## 5|6                           14.84456019   463.544312
## 6|7                           56.30846681  4004.080587
## 7|8                           74.90975265 12126.095053
## Drugapomorphine                0.80712704     4.326742
## Drugmethylphenidate            0.76246735     4.000055
## SessionT15                     0.48870933     2.549415
## SessionT60                     0.83321245     4.382207
## age                            0.98411755     1.029302
## sexmale                        0.88225234     2.252615
## sedationyes                    0.43537591     1.400149
## strata≥MCS                     0.57065905     1.326453
## Drugapomorphine:SessionT15     0.50667127     5.793173
## Drugmethylphenidate:SessionT15 0.17615075     1.810622
## Drugapomorphine:SessionT60     0.15544812     1.722267
## Drugmethylphenidate:SessionT60 0.16150881     1.672949
```

Combine odds ratios and their confidence intervals into a data frame

```r
results <- data.frame(
  Odds_Ratios = odds_ratios,
  CI_Lower = odds_ratio_ci[, 1],
  CI_Upper = odds_ratio_ci[, 2]
)
print(results)
```

```
##                                Odds_Ratios    CI_Lower     CI_Upper
## 0|1                              0.4194345  0.08331459     2.111579
## 1|2                              1.3116830  0.26284965     6.545613
## 2|3                              3.9675118  0.78884812    19.954601
## 3|4                              8.2023831  1.61845569    41.569930
## 4|5                             23.3454450  4.50537119   120.968901
## 5|6                             82.9524649 14.84456019   463.544312
## 6|7                            474.8301158 56.30846681  4004.080587
## 7|8                            953.0806792 74.90975265 12126.095053
## Drugapomorphine                  1.8687511  0.80712704     4.326742
## Drugmethylphenidate              1.7463996  0.76246735     4.000055
## SessionT15                       1.1162092  0.48870933     2.549415
## SessionT60                       1.9108399  0.83321245     4.382207
```

```
## age                              1.0064563  0.98411755     1.029302
## sexmale                          1.4097429  0.88225234     2.252615
## sedationyes                      0.7807632  0.43537591     1.400149
## strata≥MCS                       0.8700301  0.57065905     1.326453
## Drugapomorphine:SessionT15       1.7132525  0.50667127     5.793173
## Drugmethylphenidate:SessionT15   0.5647498  0.17615075     1.810622
## Drugapomorphine:SessionT60       0.5174197  0.15544812     1.722267
## Drugmethylphenidate:SessionT60   0.5198038  0.16150881     1.672949
```

Check Multicollinearity with VIF

```
lm_model <- lm(as.numeric(Score) ~ Drug + Session, data = combined_test_sum)
vif_results <- car::vif(lm_model)
print(vif_results)

##         GVIF Df GVIF^(1/(2*Df))
##  Drug      1  2               1
## Session    1  2               1
```

Check Normality of Random Effects

Extract random effects and plot Q-Q plot

```
ranef_combined <- ranef(clmm_model_combined)$Subject[[1]] # Extract the rand
om effects for subjects
qqnorm(ranef_combined, main = "Q-Q Plot of Random Effects")
qqline(ranef_combined)
```

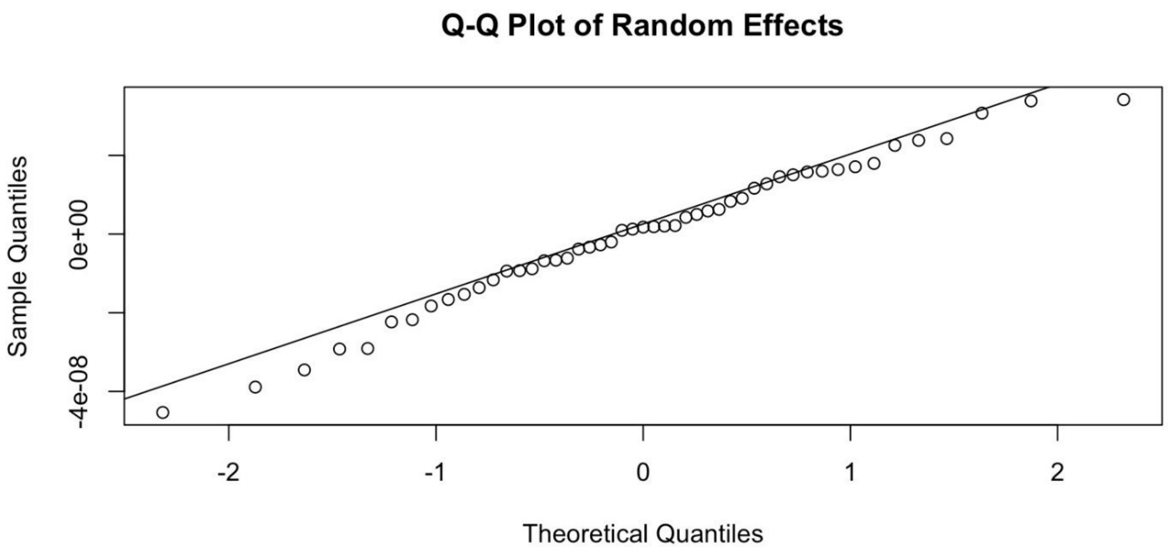

Creating a data frame for plotting based on calculated results

```
drug_effects <- data.frame(
  Drug = c("Placebo", "Apomorphine", "Methylphenidate"),
  Odds_Ratios = c(1, 1.8687511, 1.7463996),
  CI_Lower = c(1, 0.80712704, 0.76246735),
  CI_Upper = c(1, 4.326742, 4.000055)
)
```

Reorder the levels of the Drug factor to place “Placebo” first

```
drug_effects$Drug <- factor(drug_effects$Drug,
                            levels = c("Placebo", "Apomorphine",
                                       "Methylphenidate"))
```

Define the colors for each drug, aligned with the new order

```
drug_colors <- c("Placebo" = "darkgrey", "Apomorphine" = "dodgerblue4",
                 "Methylphenidate" = "forestgreen")
```

Plot the overall effects compared to placebo

```
odds_ratio_plot <- ggplot(drug_effects, aes(x = Drug, y = Odds_Ratios, color
= Drug)) +
  geom_point(size = 5, alpha = 1) +  # Plot points
  geom_errorbar(aes(ymin = CI_Lower, ymax = CI_Upper), width = 0.2, size = 1,
alpha = 0.8) +  # Error bars with horizontal caps
  geom_hline(yintercept = 1, linetype = "dashed", color = "red") +
  labs(title = "Pupillary Dilations",
       x = NULL,
       y = "Odds Ratio (log scale)") +
  scale_color_manual(values = drug_colors) +  # Set manual colors for drugs
  theme_minimal() +
  theme(
    plot.title = element_text(hjust = 0.5),
    legend.title = element_blank(),
    axis.text.x = element_text(angle = 45, hjust = 1) # Tilt the x-axis labe
ls for readability
  )

## Warning: Using `size` aesthetic for lines was deprecated in ggplot2 3.4.0.
## ℹ Please use `linewidth` instead.
## This warning is displayed once every 8 hours.
## Call `lifecycle::last_lifecycle_warnings()` to see where this warning was
## generated.
```

Show the plot

```
print(odds_ratio_plot)
```

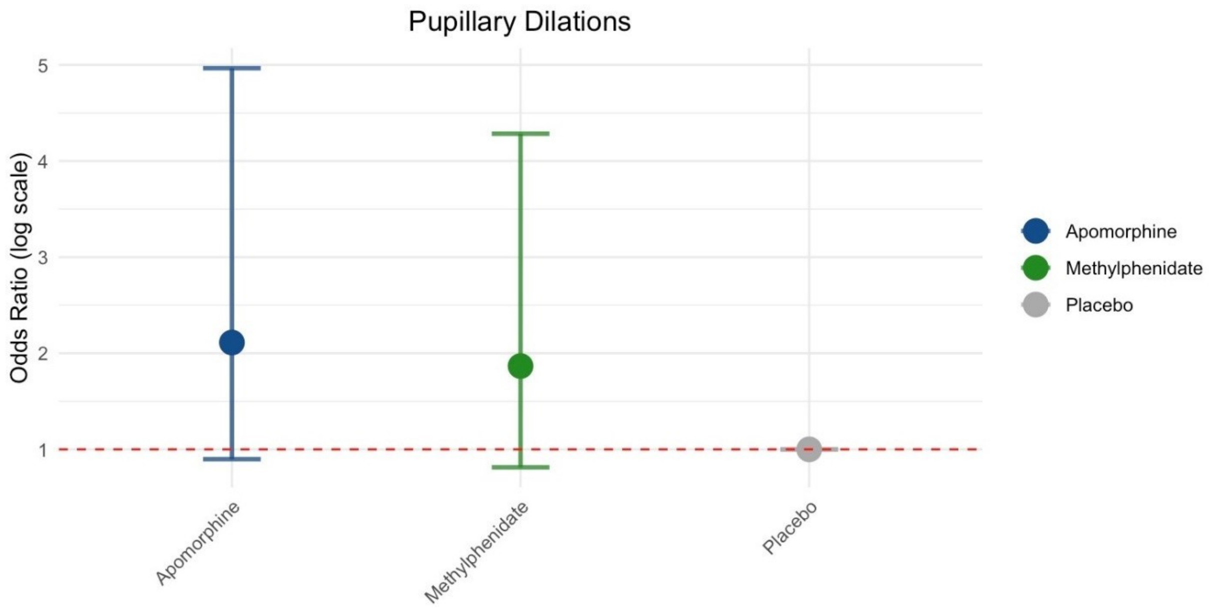
Pupillary Dilations
Odds Ratio (log scale)
5
4
3
2
1
Apomorphine
Methylphenidate
Placebo
Apomorphine
Methylphenidate
Placebo

METHODS S5

R code and plots for clinical effects of stimulants

GLMM for improved arousal with co-variates

Start with a clean environment

```
rm(list = ls())
```

Load necessary libraries

```
library(lme4)     # For GLMMs

## Loading required package: Matrix

library(dplyr)    # For data manipulation

##
## Attaching package: 'dplyr'

## The following objects are masked from 'package:stats':
##
##     filter, lag

## The following objects are masked from 'package:base':
##
##     intersect, setdiff, setequal, union

library(ggplot2)  # For plotting
library(readxl)   # For reading Excel files
library(scales)   # For formatting scales in ggplot2
library(DHARMa)   # For residual diagnostics

## This is DHARMa 0.4.6. For overview type '?DHARMa'. For recent changes, typ
e news(package = 'DHARMa')
```

Load and prepare data

```
data3 <- read_excel("~/Desktop/Clinical Data GLMM.xlsx")
data3$drug <- factor(data3$drug, levels = c("Placebo", "Methylphenidate", "Ap
omorphine"))
data3$behavior_change <- factor(data3$behavior_change, levels = c("no", "yes"
))
data3$record_id <- as.factor(data3$record_id)
```

Fit the GLMM without covariates

```
model_behavior_simple <- glmer(behavior_change ~ drug + (1 | record_id),
                               data = data3,
                               family = binomial(link = "logit"), nAGQ = 100)
```

Fit the GLMM with covariates

```
model_behavior <- glmer(behavior_change ~ drug + sex + age + sedation + strat
a + (1 | record_id),
                        data = data3,
                        family = binomial(link = "logit"), nAGQ = 100)
```

Compare the models using ANOVA

```
anova(model_behavior_simple, model_behavior, test = "Chisq")

## Data: data3
## Models:
## model_behavior_simple: behavior_change ~ drug + (1 | record_id)
## model_behavior: behavior_change ~ drug + sex + age + sedation + strata + (
1 | record_id)
##                       npar    AIC     BIC  logLik deviance  Chisq Df Pr(>C
hisq)
## model_behavior_simple    4 77.538  88.412 -34.769   69.538
## model_behavior           8 84.409 106.157 -34.204   68.409 1.1292  4     0
.8896
```

Summary of the model to check convergence and output

```
summary_model <- summary(model_behavior)
print(summary_model)

## Generalized linear mixed model fit by maximum likelihood (Adaptive
##   Gauss-Hermite Quadrature, nAGQ = 100) [glmerMod]
##  Family: binomial  ( logit )
## Formula: behavior_change ~ drug + sex + age + sedation + strata + (1  |
##     record_id)
##    Data: data3
##
##      AIC      BIC   logLik deviance df.resid
##     84.4    106.2    -34.2     68.4      104
##
## Scaled residuals:
##     Min      1Q  Median      3Q     Max
## -0.7199 -0.2937 -0.2148 -0.1049  4.5346
##
## Random effects:
##  Groups    Name        Variance Std.Dev.
##  record_id (Intercept) 1.608    1.268
```

```
## Number of obs: 112, groups:  record_id, 50
##
## Fixed effects:
##                      Estimate Std. Error z value Pr(>|z|)
## (Intercept)          -1.63795    3.24920  -0.504   0.6142
## drugMethylphenidate   2.17601    1.21269   1.794   0.0728 .
## drugApomorphine       1.56871    1.23358   1.272   0.2035
## sexmale               0.20850    1.07642   0.194   0.8464
## age                  -0.04482    0.05393  -0.831   0.4060
## sedationyes          -0.29800    1.38235  -0.216   0.8293
## strata≥MCS            0.17668    0.88805   0.199   0.8423
## ---
## Signif. codes:  0 '***' 0.001 '**' 0.01 '*' 0.05 '.' 0.1 ' ' 1
##
## Correlation of Fixed Effects:

## Warning in abbreviate(rn, minlength = 6): abbreviate used with non-ASCII c
hars

##             (Intr) drgMth drgApm sexmal age    sdtnys
## drgMthylphn -0.252
## drugApmrphn -0.272  0.785
## sexmale      0.098 -0.038  0.044
## age         -0.878 -0.073 -0.050 -0.369
## sedationyes -0.048  0.005  0.027 -0.121  0.041
## strata≥MCS  -0.372 -0.149 -0.048 -0.191  0.388  0.092
```

Check convergence using optinfo

```
convergence_status <- model_behavior@optinfo$conv$lme4$messages
```

Interpret the convergence status

```
if (is.null(convergence_status)) {
  print("Model converged successfully.")
} else {
  print("Model did not converge. Check the following convergence messages:")
  print(convergence_status)
}

## [1] "Model converged successfully."
```

Calculate odds ratios and confidence intervals

```
odds_ratios <- exp(fixef(model_behavior))
conf_int <- confint(model_behavior, parm = "beta_", level = 0.95)

## Computing profile confidence intervals ...

odds_ratio_ci <- exp(conf_int)
```

Print the odds ratios and confidence intervals

```
print(odds_ratios)

##         (Intercept) drugMethylphenidate     drugApomorphine
xmale
##           0.1943786           8.8110604           4.8004318
18325
##                 age         sedationyes         strata≥MCS
##           0.9561740           0.7423007           1.1932494

print(odds_ratio_ci)

##                             2.5 %     97.5 %
## (Intercept)         0.0001540271 711.958555
## drugMethylphenidate 1.2031545760 218.884685
## drugApomorphine     0.5516504452 114.520101
## sexmale             0.1512865296  34.655477
## age                 0.8099225228   1.054220
## sedationyes         0.0144242326  11.116244
## strata≥MCS          0.0895451152   6.014121
```

Combine odds ratios and their confidence intervals into a data frame

```
results <- data.frame(
  Odds_Ratios = odds_ratios,
  CI_Lower = odds_ratio_ci[, 1],
  CI_Upper = odds_ratio_ci[, 2]
)
```

Print the results

```
print(results)

##                     Odds_Ratios     CI_Lower   CI_Upper
## (Intercept)           0.1943786 0.0001540271 711.958555
## drugMethylphenidate   8.8110604 1.2031545760 218.884685
## drugApomorphine       4.8004318 0.5516504452 114.520101
## sexmale               1.2318325 0.1512865296  34.655477
## age                   0.9561740 0.8099225228   1.054220
## sedationyes           0.7423007 0.0144242326  11.116244
## strata≥MCS            1.1932494 0.0895451152   6.014121
```

Simpler model

Fit the GLM (without random effects)

```
glm_model <- glm(behavior_change ~ drug,
                 data = data3,
                 family = binomial(link = "logit"))
```

Summary of the model to check output

```
summary_glm <- summary(glm_model)
print(summary_glm)

##
## Call:
## glm(formula = behavior_change ~ drug, family = binomial(link = "logit"),
##     data = data3)
##
## Coefficients:
##                       Estimate Std. Error z value Pr(>|z|)
## (Intercept)             -3.584      1.014  -3.535 0.000408 ***
## drugMethylphenidate      2.064      1.096   1.882 0.059780 .
## drugApomorphine          1.504      1.144   1.315 0.188640
## ---
## Signif. codes:  0 '***' 0.001 '**' 0.01 '*' 0.05 '.' 0.1 ' ' 1
##
## (Dispersion parameter for binomial family taken to be 1)
##
##     Null deviance: 76.272  on 111  degrees of freedom
## Residual deviance: 71.018  on 109  degrees of freedom
## AIC: 77.018
##
## Number of Fisher Scoring iterations: 6
```

Creating a data frame for plotting based on calculated results

```
drug_effects <- data.frame(
  Drug = c("Placebo", "Methylphenidate", "Apomorphine"),
  Odds_Ratios = c(1, 8.8110604,4.8004318),
  CI_Lower = c(1, 1.2031545760, 0.5516504452),
  CI_Upper = c(1, 218.884685, 114.520101)
)
```

Define the colors for each drug

```
drug_colors <- c("Placebo" = "darkgrey", "Methylphenidate" = "forestgreen", "
Apomorphine" = "dodgerblue4")
```

Plot the GLMM results

```r
odds_ratio_plot <- ggplot(drug_effects, aes(x = Drug, y = Odds_Ratios, color
= Drug)) +
  geom_point(size = 5, alpha = 1) +  # Plot points
  geom_errorbar(aes(ymin = CI_Lower, ymax = CI_Upper), width = 0.2, size = 1,
alpha = 0.8) +  # Error bars with horizontal caps
  geom_hline(yintercept = 1, linetype = "dashed", color = "red") +
  labs(title = "Arousal",
       x = NULL,
       y = "Odds Ratio (log scale)") +
  scale_y_log10(breaks = scales::trans_breaks("log10", function(x) 10^x),
                labels = scales::trans_format("log10", scales::math_format(10
^.x))) +
  scale_color_manual(values = drug_colors) +  # Set manual colors for drugs
  theme_minimal() +
  theme(
    plot.title = element_text(hjust = 0.5),
    legend.title = element_blank()
  )
```

```
## Warning: Using `size` aesthetic for lines was deprecated in ggplot2 3.4.0.
## ℹ Please use `linewidth` instead.
## This warning is displayed once every 8 hours.
## Call `lifecycle::last_lifecycle_warnings()` to see where this warning was
## generated.
```

```r
print(odds_ratio_plot)
```

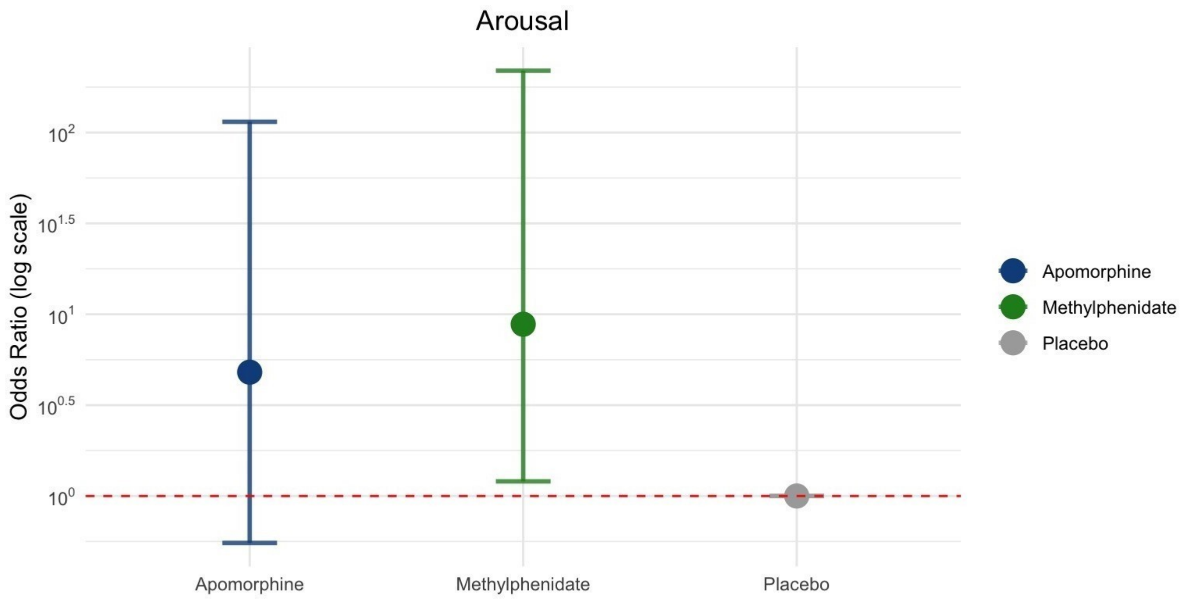

ASSUMPTIONS GLMM

1. Check for Overdispersion:

```
overdispersion_test <- sum(residuals(model_behavior, type = "pearson")^2) / d
f.residual(model_behavior)
print(overdispersion_test)

## [1] 0.5606305
```

Check if overdispersion is significantly greater than 1

```
if (overdispersion_test > 1) {
  print("Overdispersion detected!")
} else {
  print("No overdispersion detected.")
}

## [1] "No overdispersion detected."
```

2. Check Residuals for Patterns:

```
sim_res <- simulateResiduals(fittedModel = model_behavior)
```

Plot residuals

```
plot(sim_res)
```

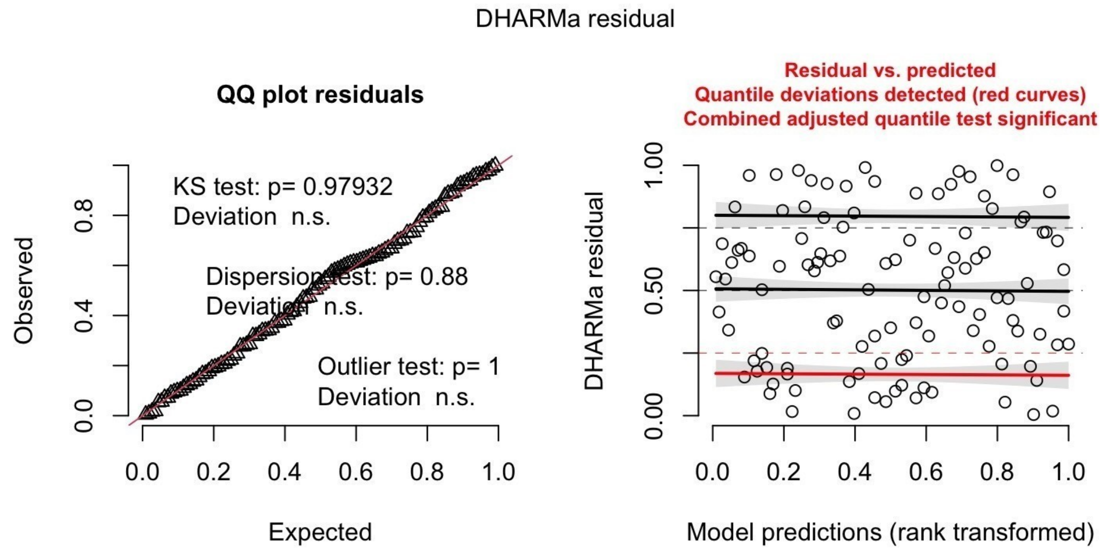

Additional checks for uniformity, dispersion, and outliers

```
testDispersion(sim_res)  # Test for dispersion
```

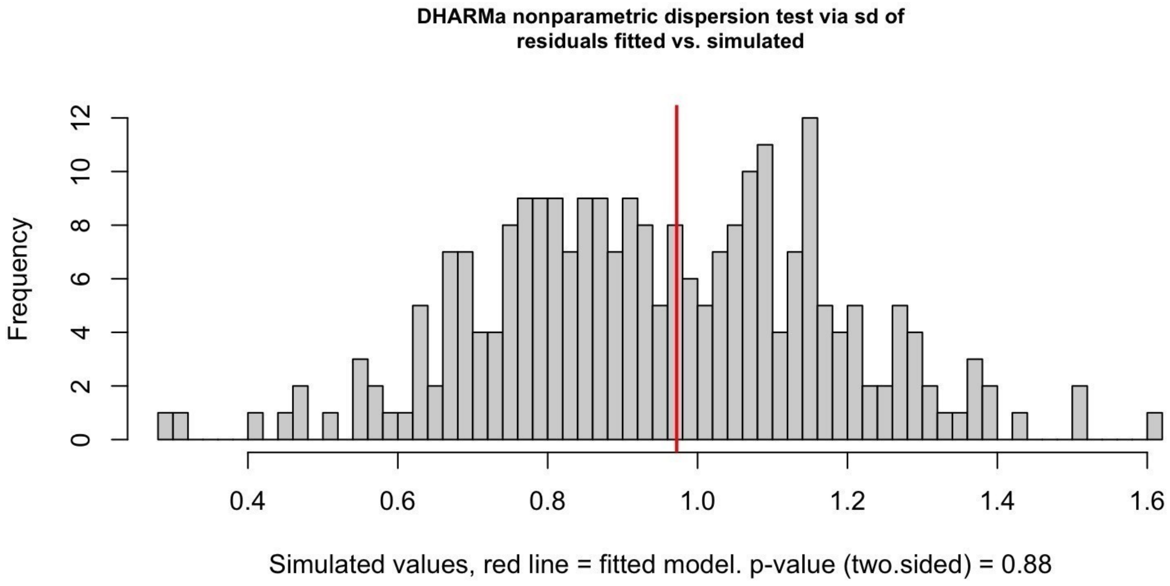


```
##
##  DHARMa nonparametric dispersion test via sd of residuals fitted vs.
##  simulated
##
## data:  simulationOutput
## dispersion = 1.0331, p-value = 0.936
## alternative hypothesis: two.sided

testUniformity(sim_res)  # Test for uniformity of residuals
```

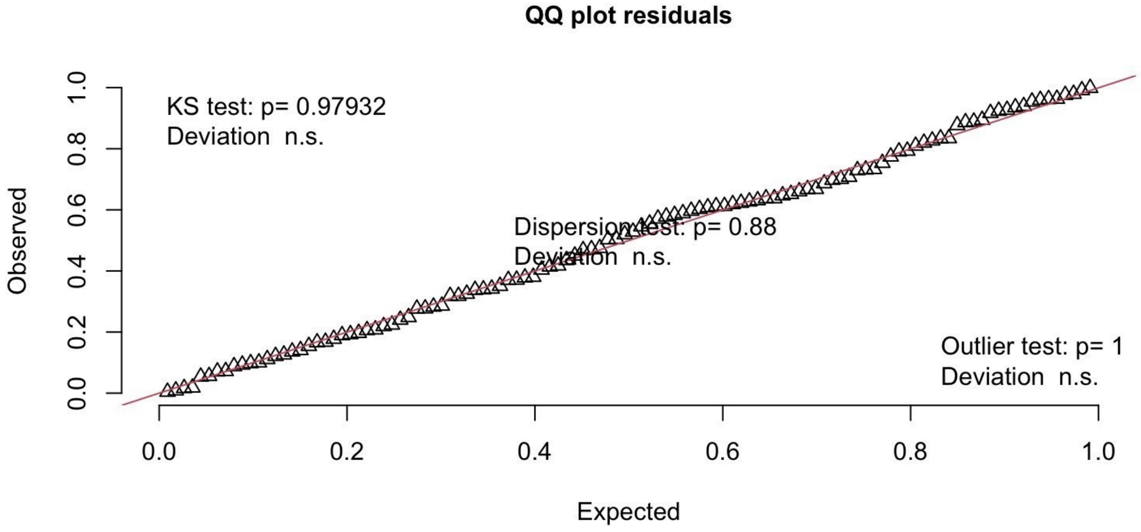

```
##
##  Asymptotic one-sample Kolmogorov-Smirnov test
##
## data:  simulationOutput$scaledResiduals
## D = 0.070092, p-value = 0.641
## alternative hypothesis: two-sided

testOutliers(sim_res)     # Test for outliers
```

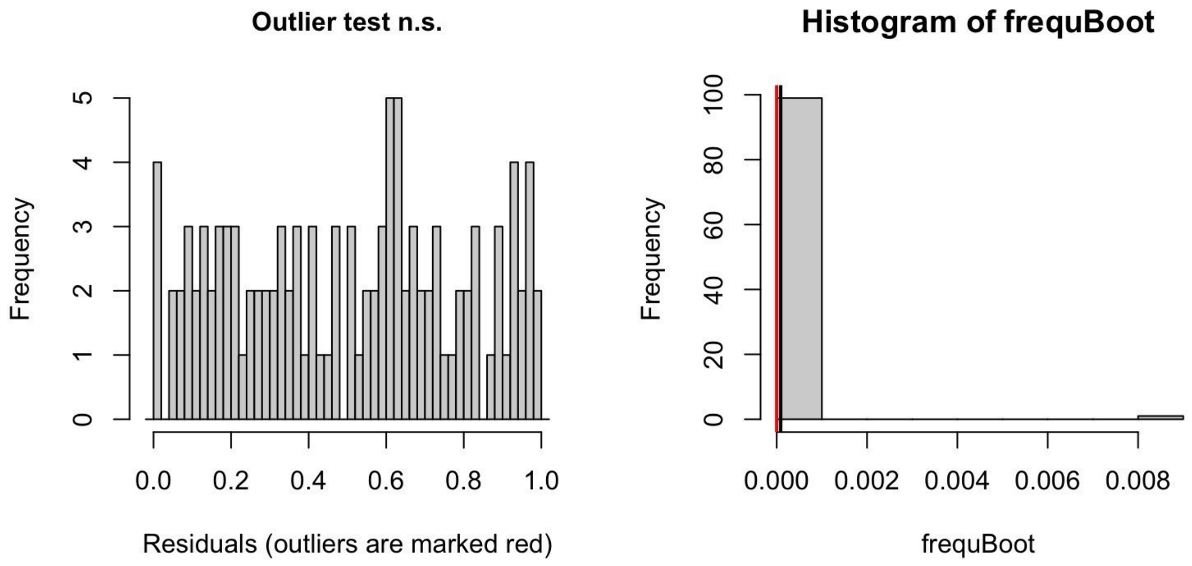


```
##
##  DHARMa bootstrapped outlier test
##
## data:  sim_res
## outliers at both margin(s) = 0, observations = 112, p-value = 1
## alternative hypothesis: two.sided
##  percent confidence interval:
##  0 0
## sample estimates:
## outlier frequency (expected: 0.000178571428571428 )
##                                                     0
```

GLMM for change towards higher level of consciousness (Binary Outcome)

Good with a clear memory before starting

```
rm(list = ls())
```

Load necessary libraries

```
library(lme4)     # For GLMMs

## Loading required package: Matrix

library(forcats) # For factor manipulation
library(ordinal) # For ordinal model
library(dplyr)    # For data manipulation

##
## Attaching package: 'dplyr'

## The following object is masked from 'package:ordinal':
##
##     slice

## The following objects are masked from 'package:stats':
##
##     filter, lag

## The following objects are masked from 'package:base':
##
##     intersect, setdiff, setequal, union

library(ggplot2)  # For plotting
library(readxl)   # For reading Excel files
library(scales)   # For formatting scales in ggplot2
library(DHARMa)   # For residual diagnostics

## This is DHARMa 0.4.6. For overview type '?DHARMa'. For recent changes,
type news(package = 'DHARMa')
```

Load and prepare data

```
data3 <- read_excel("~/Desktop/Clinical Data GLMM.xlsx")
data3$drug <- factor(data3$drug, levels = c("Placebo", "Methylphenidate",
"Apomorphine"))
data3$record_id <- as.factor(data3$record_id)
```

Set the levels for the ordinal variables

```
levels_loc <- c("Coma", "UWS", "MCS minus", "MCS plus", "eMCS")

data3$loc_before <- factor(data3$loc_before, levels = levels_loc, ordered =
TRUE)
data3$loc_after <- factor(data3$loc_after, levels = levels_loc, ordered =
TRUE)
```

Calculate the change in loc as a factor

```
data3$loc_diff = as.numeric(data3$loc_after) - as.numeric(data3$loc_before)
levels_diff = min(data3$loc_diff):max(data3$loc_diff)
data3$loc_diff = factor(data3$loc_diff, levels = levels_diff, ordered = TRUE)
```

Convert loc_diff to a binary outcome for GLMM: “Change in consciousness level - yes or no”

```
data3$consciousness_change <- ifelse(data3$loc_diff > 0, "yes", "no")
data3$consciousness_change <- factor(data3$consciousness_change, levels =
c("no", "yes"))
```

Check the distribution of the new binary outcome

```
table(data3$consciousness_change)

##
##  no yes
## 104   8
```

Fit the GLMM with random effects

```
model_glmm <- glmer(consciousness_change ~ drug + (1 | record_id),
                    data = data3,
                    family = binomial(link = "logit"), nAGQ = 100)
```

Summary of the model to check convergence and output

```
summary_model_glmm <- summary(model_glmm)
print(summary_model_glmm)

## Generalized linear mixed model fit by maximum likelihood (Adaptive
##   Gauss-Hermite Quadrature, nAGQ = 100) [glmerMod]
##  Family: binomial  ( logit )
## Formula: consciousness_change ~ drug + (1 | record_id)
##    Data: data3
##
##      AIC      BIC   logLik deviance df.resid
```

```
##      62.4      73.3     -27.2      54.4       108
##
## Scaled residuals:
##     Min      1Q  Median      3Q     Max
## -0.4979 -0.2284 -0.1770 -0.0988  4.0909
##
## Random effects:
##  Groups    Name        Variance Std.Dev.
##  record_id (Intercept) 2.167    1.472
## Number of obs: 112, groups:  record_id, 50
##
## Fixed effects:
##                      Estimate Std. Error z value Pr(>|z|)
## (Intercept)            -4.496      1.623  -2.769  0.00562 **
## drugMethylphenidate     1.226      1.285   0.954  0.34010
## drugApomorphine         1.736      1.299   1.336  0.18151
## ---
## Signif. codes:  0 '***' 0.001 '**' 0.01 '*' 0.05 '.' 0.1 ' ' 1
##
## Correlation of Fixed Effects:
##             (Intr) drgMth
## drgMthylphn -0.677
## drugApmrphn -0.762  0.746
```

Check convergence using optinfo

```
convergence_status_glmm <- model_glmm@optinfo$conv$lme4$messages
```

Interpret the convergence status

```
if (is.null(convergence_status_glmm)) {
  print("Model converged successfully.")
} else {
  print("Model did not converge. Check the following convergence messages:")
  print(convergence_status_glmm)
}

## [1] "Model converged successfully."
```

Calculate odds ratios and two-tailed 95% confidence intervals for GLMM

```
odds_ratios_glmm <- exp(fixef(model_glmm))
conf_int_glmm <- confint(model_glmm, parm = "beta_", level = 0.95)

## Computing profile confidence intervals ...

odds_ratio_ci_glmm <- exp(conf_int_glmm)
```

Combine odds ratios and their confidence intervals into a data frame

```
results_glmm <- data.frame(
  Odds_Ratios = odds_ratios_glmm,
  CI_Lower = odds_ratio_ci_glmm[, 1],
  CI_Upper = odds_ratio_ci_glmm[, 2]
)
```

Print the results

```
print(results_glmm)

##                       Odds_Ratios     CI_Lower    CI_Upper
## (Intercept)            0.01115699 7.026939e-05   0.1074574
## drugMethylphenidate    3.40664556 3.445657e-01  87.9965885
## drugApomorphine        5.67381806 6.293786e-01 169.4552960
```

Simpler model

Fit the GLM without random effects for sensitivity analysis

```
model_glm <- glm(consciousness_change ~ drug,
                 data = data3,
                 family = binomial(link = "logit"))
```

Summary of the model to check output

```
summary_glm <- summary(model_glm)
print(summary_glm)

##
## Call:
## glm(formula = consciousness_change ~ drug, family = binomial(link =
"logit"),
##     data = data3)
##
## Coefficients:
##                     Estimate Std. Error z value Pr(>|z|)
## (Intercept)           -3.584      1.014  -3.535 0.000408 ***
## drugMethylphenidate    1.099      1.179   0.932 0.351230
## drugApomorphine        1.504      1.144   1.315 0.188640
## ---
## Signif. codes:  0 '***' 0.001 '**' 0.01 '*' 0.05 '.' 0.1 ' ' 1
##
## (Dispersion parameter for binomial family taken to be 1)
##
##     Null deviance: 57.639  on 111  degrees of freedom
## Residual deviance: 55.463  on 109  degrees of freedom
```

```
## AIC: 61.463
##
## Number of Fisher Scoring iterations: 6
```

Create a data frame for plotting GLMM results

```
drug_effects_glmm <- data.frame(
  Drug = c("Placebo", "Methylphenidate", "Apomorphine"),
  Odds_Ratios = c(1, odds_ratios_glmm["drugMethylphenidate"],
odds_ratios_glmm["drugApomorphine"]),
  CI_Lower = c(1, odds_ratio_ci_glmm["drugMethylphenidate", 1],
odds_ratio_ci_glmm["drugApomorphine", 1]),
  CI_Upper = c(1, odds_ratio_ci_glmm["drugMethylphenidate", 2],
odds_ratio_ci_glmm["drugApomorphine", 2])
)
```

Define the colors for each drug

```
drug_colors <- c("Placebo" = "darkgrey", "Methylphenidate" = "forestgreen",
"Apomorphine" = "dodgerblue4")
```

Plot the GLMM results

```
plot_drug_effects_glmm <- ggplot(drug_effects_glmm, aes(x = Drug, y =
Odds_Ratios, color = Drug)) +
  geom_point(size = 5, alpha = 1) +  # Plot points
  geom_errorbar(aes(ymin = CI_Lower, ymax = CI_Upper), width = 0.2, size = 1,
alpha = 0.8) +  # Error bars with horizontal caps
  geom_hline(yintercept = 1, linetype = "dashed", color = "red") +
  labs(title = "Concsiousness level category",
       x = NULL,
       y = "Odds Ratio (log scale)") +
  scale_y_log10(breaks = scales::trans_breaks("log10", function(x) 10^x),
                labels = scales::trans_format("log10",
scales::math_format(10^.x))) +
  scale_color_manual(values = drug_colors) +  # Set manual colors for drugs
  theme_minimal() +
  theme(
    plot.title = element_text(hjust = 0.5),
    legend.title = element_blank()
  )

## Warning: Using `size` aesthetic for lines was deprecated in ggplot2 3.4.0.
## ℹ Please use `linewidth` instead.
## This warning is displayed once every 8 hours.
## Call `lifecycle::last_lifecycle_warnings()` to see where this warning was
## generated.

print(plot_drug_effects_glmm)
```

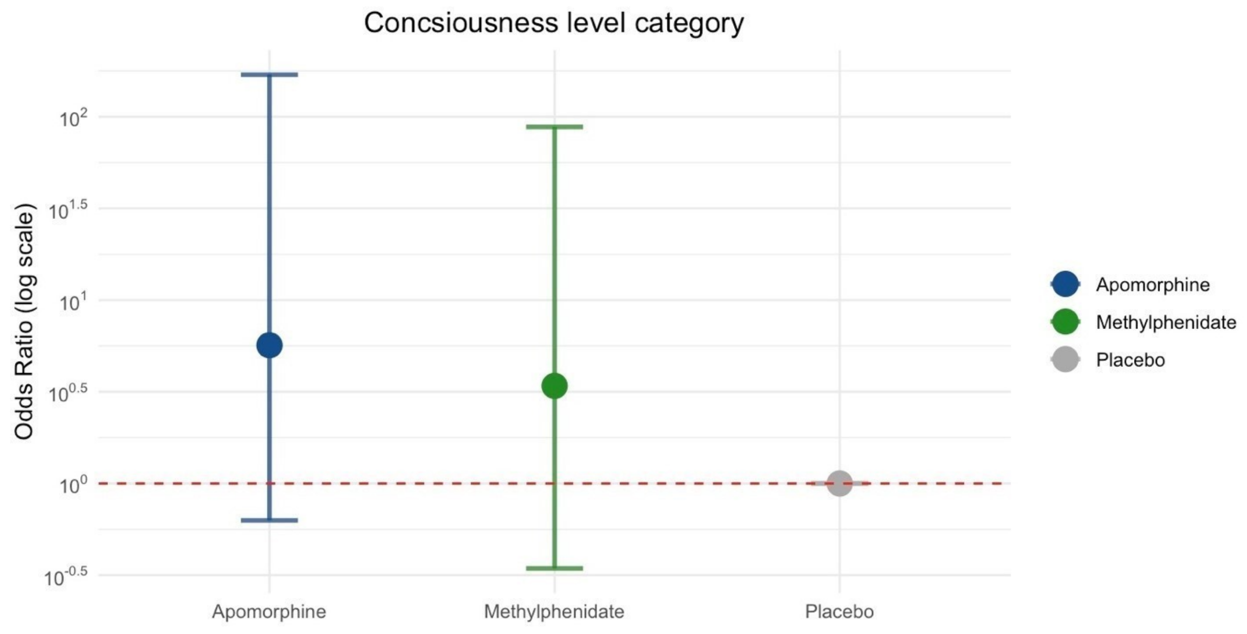

ASSUMPTIONS GLMM

1. Check for Overdispersion:

```
overdispersion_test <- sum(residuals(model_glmm, type = "pearson")^2) /
df.residual(model_glmm)
print(overdispersion_test)

## [1] 0.4423933
```

Check if overdispersion is significantly greater than 1

```
if (overdispersion_test > 1) {
  print("Overdispersion detected!")
} else {
  print("No overdispersion detected.")
}

## [1] "No overdispersion detected."
```

2. Check Residuals for Patterns:

```
sim_res <- simulateResiduals(fittedModel = model_glmm)
```

Plot residuals

```
plot(sim_res)
```

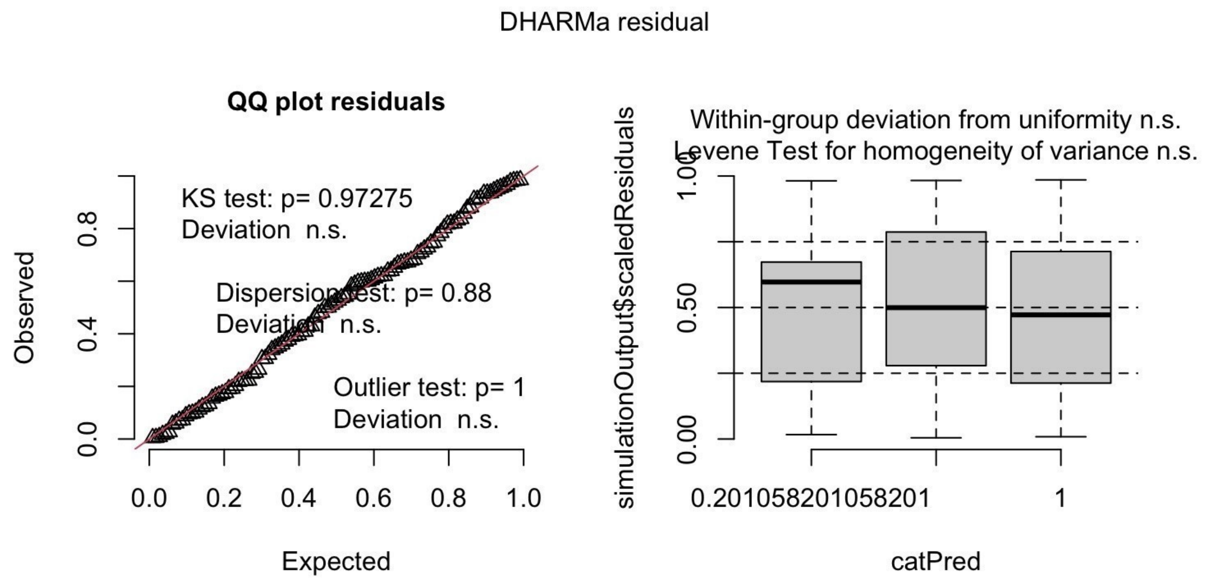

Additional checks for uniformity, dispersion, and outliers

```
testDispersion(sim_res)  # Test for dispersion
```

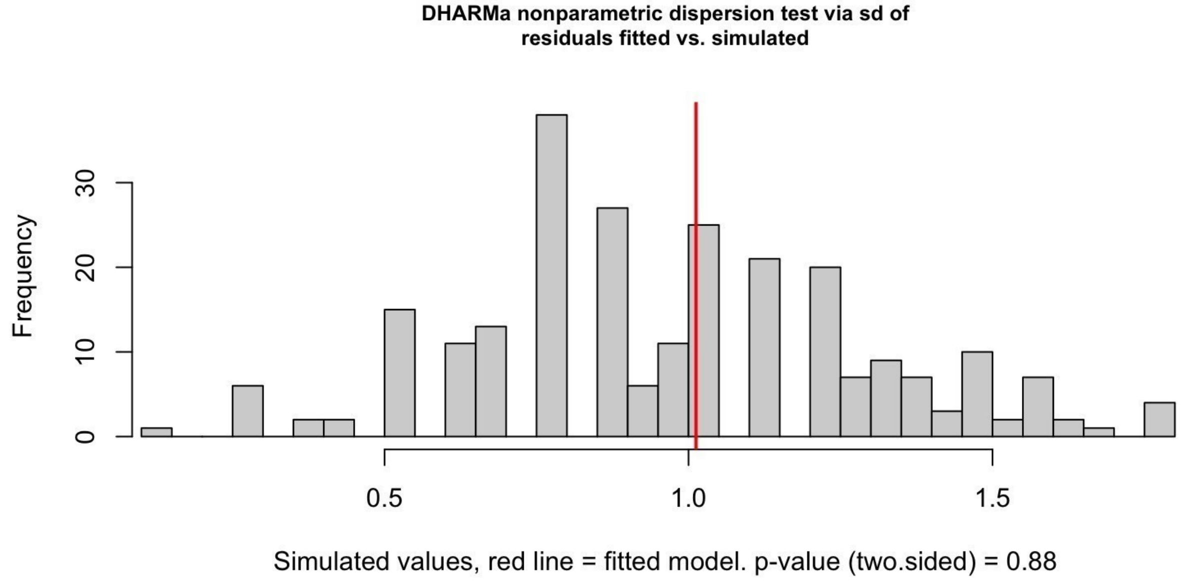


```
##
##  DHARMa nonparametric dispersion test via sd of residuals fitted vs.
##  simulated
##
## data:  simulationOutput
## dispersion = 1.01, p-value = 0.96
## alternative hypothesis: two.sided
```

```r
testUniformity(sim_res)  # Test for uniformity of residuals
```

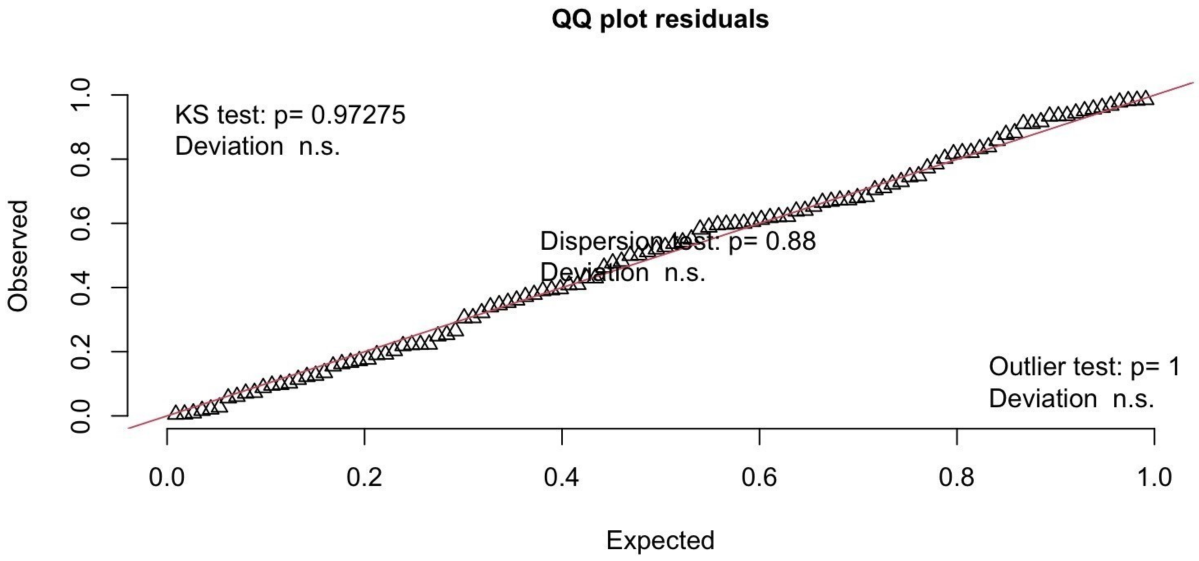


```
##
##  Asymptotic one-sample Kolmogorov-Smirnov test
##
## data:  simulationOutput$scaledResiduals
## D = 0.072309, p-value = 0.6016
## alternative hypothesis: two-sided
```

```r
testOutliers(sim_res)    # Test for outliers
```

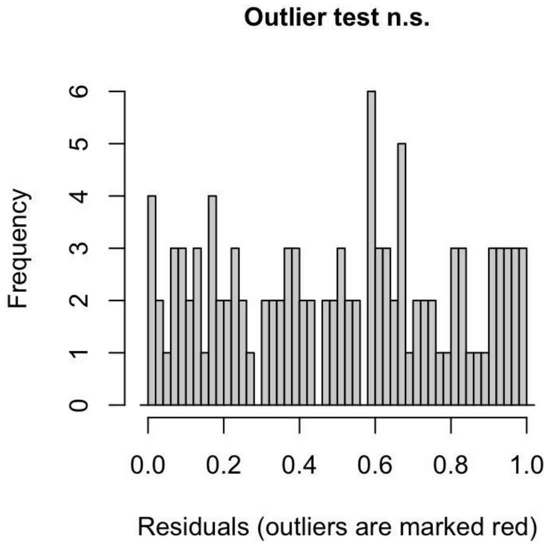

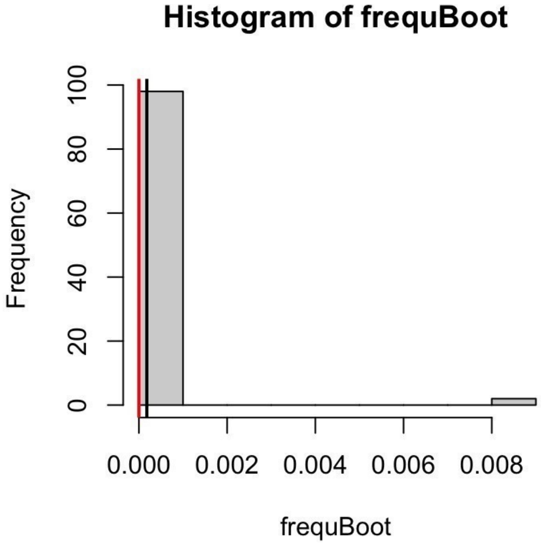

```
## 
##  DHARMa bootstrapped outlier test
## 
## data:  sim_res
## outliers at both margin(s) = 0, observations = 112, p-value = 1
## alternative hypothesis: two.sided
##  percent confidence interval:
##  0.0000000 0.0046875
## sample estimates:
## outlier frequency (expected: 0.000267857142857143 )
##                                                    0
```

Table S1: Disorder of consciousness	Definition
Coma [1]	A state of profound unawareness, unresponsive to arousal, with an absent normal sleep-wake cycle. Typically lasts a few days to three weeks post-acute brain injury.
Vegetative State (VS) / Unresponsive Wakefulness Syndrome (UWS) [2]	A state of wakefulness without awareness, where patients may open their eyes but exhibit only reflex behaviors.
Minimally Conscious State (MCS) [3,4]	A state where the patients may exhibit inconsistent yet reproducible non-reflex behaviors in response to environmental stimuli. Patients are classified as MCS if they show signs such as pain localization, visual fixation/tracking, appropriate emotional expressions (MCS minus), or if they can follow commands (MCS plus).
Emergence from MCS (eMCS) [5]	A state characterized by the recovery of functional communication (e.g., the ability to answer yes/no questions) and/or the use of objects (correctly using at least two different everyday objects).
Cognitive motor dissociation (CMD) [6]	Patients with disorders of consciousness who are behaviorally unresponsive but demonstrate volitional brain responses to motor imagery or motor commands detectable on functional magnetic resonance imaging, electroencephalography or pupillometry.

References

1. Posner, J. B., Saper, C. B., Schiff, N. D. & Claassen, J. *Plum and Posner's Diagnosis of Stupor and Coma*. (Oxford University Press, Inc, New York, 2019).
2. Laureys, S. *et al.* Unresponsive wakefulness syndrome: A new name for the vegetative state or apallic syndrome. *BMC Med* **8**, 1–4 (2010).
3. Giacino, J. T. *et al.* The minimally conscious state: definition and diagnostic criteria. *Neurology* **58**, 349–353 (2002).
4. Bruno, M. A., Vanhaudenhuyse, A., Thibaut, A., Moonen, G. & Laureys, S. From unresponsive wakefulness to minimally conscious PLUS and functional locked-in syndromes: recent

advances in our understanding of disorders of consciousness. *J Neurol* **258**, 1373–1384 (2011).

5. Nakase-Richardson, R., Yablon, S. A., Sherer, M., Evans, C. C. & Nick, T. G. Serial yes/no reliability after traumatic brain injury: implications regarding the operational criteria for emergence from the minimally conscious state. *J Neurol Neurosurg Psychiatry* **79**, 216–218 (2008).

6. Claassen, J. *et al.* Cognitive Motor Dissociation: Gap Analysis and Future Directions. *Neurocrit Care* **40**, 81–98 (2024).

SUPPLEMENTAL RESULTS

RESULTS S1

CLMM for pupillometry effects of stimulants with covariates

After adjusting for age, sex, sedation, and strata, the impact of methylphenidate on pupillary dilation showed a numerically positive effect, but this was not statistically significant (OR 1.75, 95% CI 0.76 to 4.00, $p = 0.19$). Apomorphine similarly showed a numerically positive effect on pupillary dilation, but this too was not statistically significant (OR 1.87, 95% CI 0.81 to 4.33, $p = 0.14$). The adjustments for the covariates did not alter the associations observed with either drug. Specifically, none of the covariates (age, sex, sedation, strata) showed a significant independent association with the likelihood of greater pupillary dilation. The model converged successfully, and diagnostic tests confirmed no issues with model fit, including normality of random effects. Lastly, the likelihood ratio test ($p = 0.51$) indicated that adding the covariates did not significantly improve the model fit compared to the simpler model with just the interaction between drug and session. Therefore, the simpler model without these covariates was preferred.

RESULTS S2

GLMM for improved arousal after stimulants (with covariates)

After adjusting for age, sex, sedation, and strata, the impact of methylphenidate on improved arousal remained significant (OR 8.81, 95% CI 1.20 to 219). Apomorphine continued to show a numerically positive effect, albeit not statistically significant (OR 4.80, 95% CI 0.55 to 115).

The adjustments for the covariates did not significantly alter the associations observed with either drug. Specifically, none of the covariates (age, sex, sedation, strata) showed a significant independent association with the probability of improved arousal. The model converged successfully, and diagnostic tests confirmed no issues with model fit. Lastly, the likelihood ratio test (p = 0.89) indicate that adding the covariates did not significantly improve the model fit compared to the simpler model with just the drug variable. Therefore, the simpler model without covariates was preferred.

RESULTS S3

GLMM for shift in consciousness level category after stimulants (with covariates)

After adjusting for covariates including sex, age, sedation, and strata, methylphenidate remained associated with a numerical increase toward a higher consciousness level category compared to placebo, but this was not statistically significant (OR 5.24, 95% CI 0.37 - 10,319). Similarly, apomorphine continued to show a numerically increased probability of advancing to higher consciousness level categories, but this result also remained statistically non-significant (OR 10.34, 95% CI 0.72 - 81,804). The adjustments for these covariates did not significantly alter the overall results. The model diagnostics revealed no issues with convergence, overdispersion, or residual patterns, supporting the robustness of the model. Additionally, the likelihood ratio test (p = 0.64) indicated that adding these covariates did not significantly improve the model fit compared to the simpler model with just the drug variable. Given the broad confidence intervals, which likely resulted from the small number of observed events and model complexity, the simpler model was preferred.

RESULTS S4

We did the following additional sensitivity analyses to investigate whether subgroups with different brain injuries or residual consciousness levels might have particularly responded to study drug administrations.

1. CMD vs. non-CMD

Of the 7 CMD patients, 2 patients (28.6%) showed improved arousal upon administration of a study drug (apomorphine). Of 43 non-CMD patients, 8 (18.6%) showed improved arousal, a non-significant difference compared to CMD patients (p = 0.61, Fisher's exact test).

2. Clinical baseline arousal I

Sixty-five study drug administrations were administered at baseline arousal FOUR ≥ 9, leading to 10 (15.4%) arousal responses. Forty-seven study drug administrations were administered at baseline arousal FOUR ≤ 8, leading to 2 (4.3%) arousal responses ($p = 0.07$).

3. Clinical baseline arousal II

Fifty drug administrations were administered at baseline consciousness category MCS- or better, leading to 7 (14%) arousal responses. Sixty-two study drug administrations were administered at baseline consciousness category coma or UWS, leading to 5 (8.1%) arousal responses ($p = 0.37$).

4. ICU survival

Of 37 patients who survived to ICU discharge, 9 patients (24.3 %) showed improved arousal upon administration of a study drug. Of 13 patients who did not survive to ICU discharge, 1 (7.7%) showed improved arousal ($p = 0.26$).

5. Brain injury etiologies

Of 12 anoxic post-cardiac arrest patients, 1 patient (8.3%) showed improved arousal upon administration of a study drug (methylphenidate). Of 38 non-anoxic brain injury patients, 11 (28.9%) showed improved arousal ($p = 0.25$).

6. Brain injury locations

Of the 25 patients with an infratentorial lesion, 7 (28%) showed improved arousal upon drug administration, compared to 3 of 25 patients (12%) without an infratentorial lesion ($p = 0.29$).

Table S2. Individual patient characteristics												
Patient ID[1]	Sex	Age	Cause of admission	Sedation	Command following[2]	Oriented behaviors	Visual fixation or pursuit	Noxious stimuli	Arousal response	Brainstem reflexes[3]	Respiration	DoC level
1	M	59	Aorta dissection	No	No	No	Absent	No response	Spontaneous	Present	Above ventilator rate	UWS
1				Remifentanil 500 µg/h	No	No	Absent	No response	To loud voice	Present	Above ventilator rate	UWS
1				Remifentanil 500 µg/h	No	No	Absent	No response	To loud voice	Present	Above ventilator rate	UWS
2	M	67	Brain tumor	No	No	No	Absent	No response	Spontaneous	Present	Regular breathing	UWS
2				No	No	No	Absent	No response	To loud voice	Present	Regular breathing	UWS
2				No	No	No	Absent	No response	Spontaneous	Present	Regular breathing	UWS
3	M	60	Herpes encephalitis	No	No	Smiles contextually	Present	No response	No response	Present	Above ventilator rate	MCS minus
3				No	No	No	Present	Flexion	No response	Present	Above ventilator rate	MCS minus
3				No	Neck and limb movement, and eye blink	Smiles contextually, and resists eye-opening	Present	Not tested	To loud voice	Present	Above ventilator rate	MCS minus
4	M	58	Cardiac arrest	No	No	No	Absent	Flexion	Spontaneous	Present	Above ventilator rate	UWS
4				No	No	No	Absent	No response	Spontaneous	Present	Above ventilator rate	UWS
4				No	No	No	Absent	No response	No response	Present	Above ventilator rate	Coma
5	F	48	Cardiac arrest	No	No	Scratches head and reaches for oral tubes	Present	Localization	Spontaneous	Present	Above ventilator rate	MCS minus
6	M	47	Ischemic stroke	No	No	No	Absent	Localization	Spontaneous	Present	Above ventilator rate	MCS minus
6				No	No	Scratches nose and reaches for urine catheter	Absent	Localization	Spontaneous	Present	Above ventilator rate	MCS minus
7	M	73	Cardiac arrest	No	No	No	Absent	No response	Spontaneous	Present	Above ventilator rate	UWS
8	M	65	Carbon monoxide poisoning	No	No	No	Absent	Extension	To loud voice	Present	Above ventilator rate	UWS
8				No	No	No	Absent	Extension	To loud voice	Present	Above ventilator rate	UWS

8				No	Open/close eyes	No	Absent	Extension	To loud voice	Present	Above ventilator rate	MCS plus
9	M	77	Cardiac arrest	No	No	No	Absent	Flexion	To loud voice	Present	Above ventilator rate	UWS
9				No	No	No	Absent	Flexion	No response	Present	Above ventilator rate	Coma
9				No	Tongue and neck movement	Turns head towards loud sounds	Present	Not tested	Spontaneous	Present	Above ventilator rate	MCS plus
10	M	67	Traumatic brain injury	No	No	No	Present	Flexion	No response	Present	Above ventilator rate	MCS minus
10				No	No	No	Present	Flexion	No response	Present	Above ventilator rate	MCS minus
10				No	No	No	Present	Flexion	No response	Present	Above ventilator rate	MCS minus
11	M	64	Respiratory failure	No	No	No	Absent	No response	No response	Present	Above ventilator rate	Coma
11				No	No	No	Absent	No response	No response	Present	Above ventilator rate	Coma
12	M	64	Herpes encephalitis	No	No	No	Absent	Flexion	To pain	Present	Above ventilator rate	UWS
12				No	Neck and limb movement	No	Absent	Not tested	Spontaneous	Present	Above ventilator rate	MCS plus
13	F	71	Perioperative hypovolemic shock	Remifentanil 250 µg/h	No	No	Absent	No response	Spontaneous	Present	Above ventilator rate	UWS
13				Remifentanil 150 µg/h and Propofol 20 mg/h	No	No	Absent	No response	Spontaneous	Present	Above ventilator rate	UWS
14	F	71	Heartmate surgery	No	Blink and limb movement	No	Present	Not tested	Spontaneous	Present	Above ventilator rate	MCS plus
15	F	76	Cardiac arrest	No	No	No	Absent	Localization	To loud voice	Present	Above ventilator rate	MCS minus
16	M	74	Aorta dissection	No	No	No	Absent	Localization	Spontaneous	Present	Above ventilator rate	MCS minus
17	M	51	Intracerebral hemorrhage	No	No	No	Present	Flexion	Spontaneous	Present	Above ventilator rate	MCS minus
17				No	No	No	Present	Flexion	Spontaneous	Present	Above ventilator rate	MCS minus
17				No	No	No	Present	Flexion	Spontaneous	Present	Above ventilator rate	MCS minus

18	M	71	Intracerebral hemorrhage	No	Limb movement	No	Present	Not tested	Spontaneous	Present	Above ventilator rate	MCS plus
18				No	Limb movement	No	Present	Not tested	Spontaneous	Present	Above ventilator rate	MCS plus
18				No	Limb movement	No	Present	Not tested	Spontaneous	Present	Above ventilator rate	MCS plus
19	F	82	Herpes encephalitis	No	No	No	Absent	Flexion	No response	Present	Above ventilator rate	Coma
19				No	No	No	Absent	Flexion	No response	Present	Above ventilator rate	Coma
19				No	No	No	Absent	Flexion	Spontaneous	Present	Above ventilator rate	UWS
20	F	50	Intracerebral hemorrhage	No	No	No	Present	No response	Spontaneous	Present	Regular breathing	MCS minus
20				No	No	No	Absent	Flexion	No response	Present	Regular breathing	Coma
20				No	No	No	Absent	Flexion	To pain	Present	Regular breathing	UWS
21	M	67	Heart failure	No	No	No	Absent	Flexion	To loud voice	Present	Cheyne-Stokes	UWS
21				No	Tongue and limb movement	No	Absent	Not tested	To loud voice	Present	Above ventilator rate	MCS plus
21				No	Limb movement	No	Absent	Not tested	To loud voice	Present	Cheyne-Stokes	MCS plus
22	M	75	Herpes encephalitis	Remifentanil 400 µg/h	No	Reaches for oral tube and pupillometer	Absent	Localization	Spontaneous	Present	Above ventilator rate	MCS minus
22				No	No	No	Absent	Flexion	Spontaneous	Present	Above ventilator rate	UWS
22				No	No	No	Absent	Flexion	To pain	Present	Above ventilator rate	UWS
23	M	73	Cardiac arrest	No	No	No	Absent	No response	Spontaneous	Present	Above ventilator rate	UWS
23				No	No	No	Absent	No response	Spontaneous	Present	Above ventilator rate	UWS
24	F	55	Ischemic stroke	No	No	No	Present	Flexion	Spontaneous	Present	Regular breathing	MCS minus
24				No	No	No	Present	Flexion	No response	Present	Regular breathing	MCS minus

25	F	75	Pseudoaneurysm in aorta	Fentanyl 100 µg/h	No	No	Absent	No response	To loud voice	Present	At ventilator rate	UWS
26	M	55	Convulsive status epilepticus*	No	No	No	Absent	Localization	Spontaneous	Present	Above ventilator rate	MCS minus
27	M	61	Ischemic stroke	No	No	No	Absent	No response	No response	Present	Above ventilator rate	Coma
27				No	No	No	Absent	No response	No response	Present	Above ventilator rate	Coma
28	M	63	Perioperative hypotension	No	No	No	Absent	Flexion	No response	Present	Above ventilator rate	Coma
28				Remifentanil 300 µg/h	No	No	Absent	Flexion	Spontaneous	Present	Above ventilator rate	UWS
29	M	51	Heart transplant surgery	No	No	No	Present	Localization	Spontaneous	Present	Above ventilator rate	MCS minus
29				No	No	No	Present	Localization	Spontaneous	Present	Regular breathing	MCS minus
30	F	66	Cardiac arrest	No	No	No	Absent	No response	No response	Present	Above ventilator rate	Coma
30				No	No	No	Absent	No response	No response	Present	Above ventilator rate	Coma
30				No	No	No	Absent	No response	No response	Present	Above ventilator rate	Coma
31	M	76	Metabolic disorder	Remifentanil 300 µg/h	No	No	Absent	No response	Spontaneous	Present	Above ventilator rate	UWS
31				Remifentanil 150 µg/h	No	No	Absent	No response	Spontaneous	Present	Above ventilator rate	UWS
31				Remifentanil 150 µg/h	No	No	Absent	No response	Spontaneous	Present	Above ventilator rate	UWS
32	M	67	Covid-19	No	No	No	Absent	No response	Spontaneous	Present	Above ventilator rate	UWS
32				No	No	No	Absent	No response	Spontaneous	Present	Above ventilator rate	UWS
33	F	49	Subarachnoid hemorrhage	No	No	No	Present	No response	Spontaneous	Present	Regular breathing	MCS minus
33				No	No	No	Present	No response	Spontaneous	Present	Regular breathing	MCS minus
33				No	No	No	Present	No response	Spontaneous	Present	Regular breathing	MCS minus

34	M	51	Ischemic stroke	Remifentanil 250 µg/h	No	No	Absent	No response	Spontaneous	Present	At ventilator rate	UWS
34				Remifentanil 250 µg/h	Eye blink	No	Present	No response	To loud voice	Present	At ventilator rate	MCS plus
35	M	52	Cardiac arrest	No	No	No	Absent	Flexion	Spontaneous	Present	Above ventilator rate	UWS
35				No	No	No	Absent	Flexion	Spontaneous	Present	Above ventilator rate	UWS
35				No	No	No	Absent	Flexion	Spontaneous	Present	Above ventilator rate	UWS
36	M	79	Cardiac arrest	Remifentanil 500 µg/h	No	Turns head towards loud sounds	Absent	No response	Spontaneous	Present	Above ventilator rate	MCS minus
37	M	56	Traumatic brain injury	Remifentanil 200 µg/h	No	No	Present	Flexion	Spontaneous	Present	Above ventilator rate	MCS minus
37				No	No	No	Present	Flexion	Spontaneous	Present	Above ventilator rate	MCS minus
38	M	53	Aorta dissection	No	Limb movement and eye blink	No	Absent	Not tested	To loud voice	Present	Above ventilator rate	MCS plus
38				No	Limb movement and eye blink	No	Present	Not tested	Spontaneous	Present	Above ventilator rate	MCS plus
39	F	59	Lung transplant surgery	No	No	No	Absent	Flexion	Spontaneous	Present	Above ventilator rate	UWS
39				No	No	No	Absent	Flexion	Spontaneous	Present	Above ventilator rate	UWS
39				No	No	No	Absent	Flexion	Spontaneous	Present	Above ventilator rate	UWS
40	M	66	Cardiac arrest	No	No	No	Absent	Flexion	No response	Present	Above ventilator rate	Coma
40				No	No	No	Absent	Localization	To pain	Present	Above ventilator rate	MCS minus
40				No	No	No	Present	Localization	Spontaneous	Present	Above ventilator rate	MCS minus
41	F	68	Septic shock	No	No	No	Absent	No response	No response	Present	Above ventilator rate	Coma
42	M	59	Ischemic stroke	No	No	No	Absent	No response	Spontaneous	Present	Above ventilator rate	UWS
42				No	No	No	Absent	No response	No response	Present	Above ventilator rate	Coma

42				No	No	No	Absent	Flexion	Spontaneous	Present	Above ventilator rate	UWS
43	F	51	Brain tumor	No	Limb movement	No	Absent	Not tested	Spontaneous	Present	Regular breathing	MCS plus
43				No	Limb movement, eye blink, and open/close eyes	No	Absent	Not tested	Spontaneous	Present	Regular breathing	MCS plus
43				No	Tongue and limb movement	Crosses fingers and removes pulse oximetry	Absent	Not tested	Spontaneous	Present	Regular breathing	MCS plus
44	M	73	Aorta dissection	No	Limb movement	No	Absent	Not tested	Spontaneous	Present	Regular breathing	MCS plus
45	M	73	Aorta aneurysm rupture	No	No	No	Absent	No response	To loud voice	Present	Above ventilator rate	UWS
45				No	No	No	Absent	Flexion	Spontaneous	Present	Above ventilator rate	UWS
45				No	No	No	Present	Localization	Spontaneous	Present	Above ventilator rate	MCS minus
46	M	64	Subarachnoid hemorrhage	No	No	No	Present	No response	To loud voice	Present	Above ventilator rate	MCS minus
46				No	Open/close eyes	No	Present	Not tested	Spontaneous	Present	Above ventilator rate	MCS plus
47	M	57	Intracerebral hemorrhage	No	No	Grabs sheets and reaches for headphones	Absent	Flexion	Spontaneous	Present	Above ventilator rate	MCS minus
48	M	55	Cardiac arrest	No	No	No	Absent	Flexion	To pain	Present	Above ventilator rate	UWS
48				No	No	No	Absent	Flexion	To pain	Present	Above ventilator rate	UWS
49	M	66	Cardiac arrest	No	No	No	Absent	Flexion	Spontaneous	Present	Above ventilator rate	UWS
49				No	No	No	Absent	Flexion	Spontaneous	Present	Above ventilator rate	UWS
49				No	No	Laughing and smiling contextually	Absent	Flexion	Spontaneous	Present	Above ventilator rate	MCS minus
50	F	49	Intracerebral hemorrhage	No	No	No	Absent	No response	Spontaneous	Absent	Regular breathing	UWS
50				No	No	No	Absent	No response	Spontaneous	Absent	Regular breathing	UWS

[1] Each patient ID corresponds to multiple sessions, with repeated IDs indicating separate, chronologically ordered sessions.

[2] Limb movement encompasses all hand gestures.

[3] **The following brainstem reflexes were assessed: pupillary light reflex, corneal- and ciliary reflex, gag- and cough reflex.**

***The patient was no longer in status epilepticus when DoC evaluation was performed (as evidenced by EEG). Cause of consciousness impairment was either due to Wernicke's encephalopathy from alcohol abuse or prolonged postictal phase.**

DoC - disorders of consciousness, F – female, M – male, MCS - minimally conscious state, UWS - unresponsive responsiveness syndrome

Table S3. Plasma levels of stimulant drugs				
**ID**	**Session**	**Administered drug**<br>**Baseline**	**Concentration (μg/kg)**<br>**T_{15}**	**Concentration (μg/kg)**<br>**T_{60}**
**Patient 1**				
	**1**	Apomorphine	4.92	2.66
	**2**	Placebo	-	-
	**3**	Methylphenidate	6.55	14.55
**Patient 2**				
	**1**	Methylphenidate	9.79	10.53
	**2**	Apomorphine	5.02	2.50
	**3**	Placebo	-	-
**Patient 3**				
	**1**	Methylphenidate	5.38	3.44
	**2**	Apomorphine	5.93	0.98
	**3**	Placebo	-	-
**Patient 4**				
	**1**	Apomorphine	8.00	4.97
	**2**	Methylphenidate	1.87	43.87
	**3**	Placebo	-	-
**Patient 5**				

	**1**	Apomorphine	7.08	N/A
**Patient 6**				
	**1**	Placebo	-	-
	**2**	Methylphenidate	11.43	10.04
**Patient 7**				
	**1**	Methylphenidate	10.85	6.25
**Patient 8**				
	**1**	Placebo	-	-
	**2**	Apomorphine	16.51	8.73
	**3**	Methylphenidate	4.62	4.31
**Patient 9**				
	**1**	Apomorphine	4.20	2.94
	**2**	Methylphenidate	4.31	-
	**3**	Placebo	-	-
**Patient 10**				
	**1**	Methylphenidate	-	2.42
	**2**	Apomorphine	8.62	2.10
	**3**	Placebo	-	-
**Patient 11**				
	**1**	Placebo	-	-

	**2**	Methylphenidate	2.30	10.99
**Patient 12**				
	**1**	Methylphenidate	2.13	1.18
	**2**	Placebo	-	-
**Patient 13**				
	**1**	Placebo	-	-
	**2**	Apomorphine	4.01	3.17
**Patient 14**				
	**1**	Placebo	-	-
**Patient 15**				
	**1**	Placebo	-	-
**Patient 16**				
	**1**	Apomorphine	4.27	2.53
**Patient 17**				
	**1**	Apomorphine	2.96	3.02
	**2**	Placebo	-	-
	**3**	Methylphenidate	2.08	3.03
**Patient 18***				
	**1**	Methylphenidate	4.73	2.62
	**2**	Placebo	-	-

	**3**	Methylphenidate	4.78	3.50
**Patient 19**				
	**1**	Methylphenidate	2.12	1.18
	**2**	Apomorphine	6.06	3.38
	**3**	Placebo	-	-
**Patient 20**				
	**1**	Methylphenidate	22.83	9.73
	**2**	Placebo	-	-
	**3**	Apomorphine	14.99	3.01
**Patient 21***				
	**1**	Apomorphine	7.03	3.86
	**2**	Apomorphine	9.51	5.59
	**3**	Methylphenidate	13.94	7.35
**Patient 22**				
	**1**	Apomorphine	7.43	4.46
	**2**	Methylphenidate	1.70	5.04
	**3**	Placebo	-	-
**Patient 23**				
	**1**	Apomorphine	2.82	2.14
	**2**	Methylphenidate	17.54	8.84

**Patient 24**				
	**1**	Apomorphine	7.27	3.49
	**2**	Placebo	-	-
**Patient 25**				
	**1**	Placebo	-	-
**Patient 26**				
	**1**	Methylphenidate	27.11	20.40
**Patient 27**				
	**1**	Apomorphine	1.64	3.02
	**2**	Placebo	-	-
**Patient 28**				
	**1**	Methylphenidate	10.62	12.07
	**2**	Placebo	-	-
**Patient 29**				
	**1**	Methylphenidate	29.68	28.22
	**2**	Apomorphine	8.62	4.59
**Patient 30**				
	**1**	Methylphenidate	3.40	4.78
	**2**	Apomorphine	7.91	1.88
	**3**	Placebo	-	-

**Patient 31**				
	**1**	Apomorphine	6.01	3.93
	**2**	Placebo	-	-
	**3**	Methylphenidate	14.48	17.89
**Patient 32**				
	**1**	Apomorphine	8.77	3.86
	**2**	Methylphenidate	7.26	12.15
**Patient 33**				
	**1**	Placebo	N/A	N/A
	**2**	Apomorphine	N/A	N/A
	**3**	Methylphenidate	N/A	N/A
**Patient 34**				
	**1**	Placebo	-	-
	**2**	Methylphenidate	0.51	3.21
**Patient 35**				
	**1**	Placebo	-	-
	**2**	Apomorphine	5.51	2.34
	**3**	Methylphenidate	7.28	5.46
**Patient 36**				
	**1**	Placebo	-	-

**Patient 37**				
	**1**	Apomorphine	8.26	N/A
	**2**	Placebo	-	-
**Patient 38***				
	**1**	Methylphenidate	-	1.18
	**2**	Methylphenidate	4.50	3.72
**Patient 39***				
	**1**	Apomorphine	10.53	5.91
	**2**	Apomorphine	15.26	11.27
	**3**	Methylphenidate	14.81	37.26
**Patient 40**				
	**1**	Placebo	-	-
	**2**	Apomorphine	5.28	3.55
	**3**	Methylphenidate	1.98	N/A
**Patient 41**				
	**1**	Apomorphine	5.19	4.94
**Patient 42**				
	**1**	Methylphenidate	5.25	9.69
	**2**	Apomorphine	18.08	6.05
	**3**	Placebo	-	-

**Patient 43**				
	**1**	Methylphenidate	N/A	N/A
	**2**	Placebo	N/A	N/A
	**3**	Apomorphine	N/A	N/A
**Patient 44**				
	**1**	Placebo	-	-
**Patient 45**				
	**1**	Apomorphine	3.24	3.10
	**2**	Methylphenidate	21.37	32.88
	**3**	Placebo	-	-
**Patient 46**				
	**1**	Placebo	-	-
	**2**	Methylphenidate	5.42	2.88
**Patient 47**				
	**1**	Methylphenidate	0.89	4.00
**Patient 48**				
	**1**	Apomorphine	10.54	4.33
	**2**	Methylphenidate	5.36	6.27
**Patient 49**				
	**1**	Placebo	-	-

**2**	Methylphenidate	98.81	40.31
**3**	Apomorphine	3.33	4.26
**Patient 50**			
**1**	Apomorphine	7.62	1.60
**2**	Placebo	-	-

*** Patient with a protocol deviation, i.e., error in the randomization or drug preparation process**

N/A - missing data

Table S4. Patients with drug plasma levels indicating incomplete washout of previous drug*			
**ID**	**Session**	**Methylphenidate (μg/kg)**	**Apomorphine (μg/kg)**
**Patient 1**	**1 – T_{15}**	-	4.92
	**1 – T_{60}**	-	2.66
	**2 – T_{15}**	-	0.60
	**2 – T_{60}**	-	0.60
	**3 – T_{15}**	6.55	0.40
	**3 – T_{60}**	14.55	0.41
**Patient 8**	**1 – T_{15}**	-	-
	**1 – T_{60}**	-	-
	**2 – T_{15}**	-	16.51
	**2 – T_{60}**	-	8.73
	**3 – T_{15}**	4.62	0.47
	**3 – T_{60}**	4.31	0.45
**Patient 9**	**1 – T_{15}**	-	4.20
	**1 – T_{60}**	-	2.94
	**2 – T_{15}**	-	0.12
	**2 – T_{60}**	4.31	0.12
	**3 – T_{15}**	-	-
	**3 – T_{60}**	-	-
**Patient 19**	**1 – T_{15}**	2.12	-
	**1 – T_{60}**	1.18	-
	**2 – T_{15}**	2.18	6.06
	**2 – T_{60}**	2.11	3.38
	**3 – T_{15}**	-	-
	**3 – T_{60}**	-	-
**Patient 21**	**1 – T_{15}**	-	7.03
	**1 – T_{60}**	-	3.86

	**2 – T_{15}**	-	9.51
	**2 – T_{60}**	-	5.59
	**3 – T_{15}**	13.94	0.25
	**3 – T_{60}**	7.35	0.22
**Patient 23**	**1 – T_{15}**	-	2.82
	**1 – T_{60}**	-	2.14
	**2 – T_{15}**	17.54	0.06
	**2 – T_{60}**	8.84	0.06
**Patient 29**	**1 – T_{15}**	29.68	-
	**1 – T_{60}**	28.22	-
	**2 – T_{15}**	3.59	8.62
	**2 – T_{60}**	3.08	4.59
**Patient 39**	**1 – T_{15}**	-	10.53
	**1 – T_{60}**	-	5.91
	**2 – T_{15}**	-	15.26
	**2 – T_{60}**	-	11.27
	**3 – T_{15}**	14.81	0.69
	**3 – T_{60}**	37.26	0.73
**Patient 45**	**1 – T_{15}**	-	3.24
	**1 – T_{60}**	-	3.10
	**2 – T_{15}**	21.37	0.41
	**2 – T_{60}**	32.88	0.39
	**3 – T_{15}**	0.77	-
	**3 – T_{60}**	0.85	-
**Patient 48**	**1 – T_{15}**	-	10.54
	**1 – T_{60}**	-	4.33
	**2 – T_{15}**	5.36	0.06
	**2 – T_{60}**	6.27	0.07

**Patient 49**	**1 – T_{15}**	-	-
	**1 – T_{60}**	-	-
	**2 – T_{15}**	98.81	-
	**2 – T_{60}**	40.31	-
	**3 – T_{15}**	2.73	3.33
	**3 – T_{60}**	2.17	4.26

*Residual plasma levels indicating incomplete washout are highlighted in blue

Table S5. Pupillary dilations during mental arithmetic

Patient ID	Consciousness	Drug	Moderate-Level*			Hard-Level**		
			Baseline	T15	T60	Baseline	T15	T60
1	UWS	Apormorphine	1	2	1	2	3	
2	UWS	Apormorphine	3	0	1	2	0	1
3	MCS minus	Apormorphine	1	1	1	1	1	0
4	UWS	Apormorphine	1	2	3	1	3	2
5	MCS minus	Apormorphine	0	1		3	1	
8	UWS	Apormorphine	1	2	1	1	3	1
9	UWS	Apormorphine	1	0	0	1	0	0
10	MCS minus	Apormorphine	1	1	1	3	0	2
13	UWS	Apormorphine	1	3	3	3	1	2
16	MCS minus	Apormorphine	2	4	3	2	2	0
17	MCS minus	Apormorphine	1	3	1	1	1	1
19	Coma	Apormorphine	2	1	0	0	1	2
20	UWS	Apormorphine	0	2	2	0	1	2
21	UWS	Apormorphine	1	0	2	0	0	3
21	MCS plus	Apormorphine	3	4	4	1	2	3
22	MCS minus	Apormorphine	0	0	1	2	1	1
23	UWS	Apormorphine	1	1	4	4	1	1
24	MCS minus	Apormorphine	0	1	0	0		1
27	Coma	Apormorphine	2	2	1	0	1	0
29	MCS minus	Apormorphine	2	0	1	4	0	1
30	Coma	Apormorphine	1	1	0	0	1	1
31	UWS	Apormorphine	1	2	1	3	2	1
32	UWS	Apormorphine	1	3	0	0	3	3
33	MCS minus	Apormorphine	1	1		1	3	
37	MCS minus	Apormorphine	1	2		0	2	
39	UWS	Apormorphine	0	0	3	2	2	2
39	UWS	Apormorphine	3	2	0	1	1	0
41	Coma	Apormorphine	1	2	1	2	2	1
42	Coma	Apormorphine	1	3	2	1	2	2
45	UWS	Apormorphine	1	2	3	1	2	2
48	UWS	Apormorphine	0	1	2	0	3	1
49	MCS minus	Apormorphine	1	1	3	2	1	0
50	UWS	Apormorphine	0	1	1	1	0	0

1	UWS	Methylphenidate	0	0	1	1	1	2
2	UWS	Methylphenidate	2	0	1	2	2	0
3	MCS minus	Methylphenidate	1	2	1	1	0	0
4	UWS	Methylphenidate	2	4	1	0	1	1
6	MCS minus	Methylphenidate	0	0	0	1	0	
7	UWS	Methylphenidate	2	2	1	0	1	2
8	MCS plus	Methylphenidate	0	1	1	2	0	0
9	Coma	Methylphenidate	0	2	1	1	1	2
10	MCS minus	Methylphenidate	2	1	2	1	2	2
11	Coma	Methylphenidate	2	3	3	1	1	1
12	UWS	Methylphenidate	0	1	1	1	0	1
17	MCS minus	Methylphenidate	1	1	2	0	1	2
18	MCS plus	Methylphenidate	1	1	0	2	0	2
18	MCS plus	Methylphenidate	0	2	2	1	2	4
19	Coma	Methylphenidate	3	1	1	0	0	0
20	MCS minus	Methylphenidate	1	1	1	1	0	2
21	MCS plus	Methylphenidate	2	1	2	1	1	0
22	UWS	Methylphenidate	1	0	2	3	1	0
23	UWS	Methylphenidate	0	0	3	1	0	1
26	MCS minus	Methylphenidate	1	1	1	0	1	2
28	Coma	Methylphenidate	2	2	0		2	1
29	MCS minus	Methylphenidate	1	2	2	3	0	0
30	Coma	Methylphenidate	2	1	0	1	0	0
31	UWS	Methylphenidate	0	1	0	0	0	3
32	UWS	Methylphenidate	4	0	4	2	2	2
34	MCS minus	Methylphenidate	0	1	2	3	0	2
38	MCS plus	Methylphenidate	0	4	0	5	2	
39	UWS	Methylphenidate	0	0	1	0	1	1
42	UWS	Methylphenidate	3	0	1	2	2	2
43	MCS plus	Methylphenidate	1	3	0	4	1	0
45	UWS	Methylphenidate	1	0	3	2	1	2
46	MCS plus	Methylphenidate	2	0	0	0	0	3
47	MCS minus	Methylphenidate	2	0	2	0	1	0
48	UWS	Methylphenidate	1	1	2	2	2	0
49	UWS	Methylphenidate	0	2	2	0	1	0
1	UWS	Placebo	1	1	1	1	2	1
2	UWS	Placebo	3	2	3	1	0	1

3	MCS minus	Placebo	0	0	0	1	0	3
4	UWS	Placebo	3	1	2	1	0	2
6	MCS minus	Placebo	3	1	2	1	2	0
8	UWS	Placebo	1	1	2	2	0	0
9	MCS plus	Placebo	0	2	2	1	0	2
10	MCS minus	Placebo	0	0	1	0	1	1
11	Coma	Placebo	0	0	2	0	0	0
12	MCS plus	Placebo	1	4	4	3	1	1
13	UWS	Placebo	0	2	0	1	0	0
14	MCS plus	Placebo	2	0	2	3	2	2
15	MCS minus	Placebo	1	0	2	0	1	1
17	MCS minus	Placebo	0	1	2	0	2	1
18	MCS plus	Placebo	1	1	0	1	0	0
19	UWS	Placebo	1	2	1	1	1	2
20	Coma	Placebo	2	1	3	0	1	1
22	UWS	Placebo	2	0	3	2	0	1
24	MCS minus	Placebo	0	1	2	1	1	1
25	UWS	Placebo	1	2	1	0	0	2
27	Coma	Placebo	1	0	2	1	0	1
28	UWS	Placebo	1	2	1	1	2	0
30	Coma	Placebo	1	1	0	1	1	2
31	UWS	Placebo	1			3		
33	MCS minus	Placebo	1	1	2	1	2	2
34	UWS	Placebo	2	3	0	0	3	0
35	UWS	Placebo	4			0		
36	MCS minus	Placebo	0	1	2	1	4	0
37	MCS minus	Placebo	0	2	2	1	0	2
42	UWS	Placebo	0	1	1	1	2	3
44	MCS plus	Placebo	0					
45	MCS minus	Placebo	0	2	0	0	0	0
46	MCS minus	Placebo	1	2	3	0	0	2
49	UWS	Placebo	1	3	2	2	1	0
50	UWS	Placebo	0	1	1			

***The numbers represent significant pupillary dilation during a series of five moderate mental arithmetic tasks at baseline, 15 minutes (T15), and 60 minutes (T60) after drug administration.**

****The numbers represent significant pupillary dilation during a series of five hard mental arithmetic tasks at baseline, 15 minutes (T15), and 60 minutes (T60) after drug administration.**

MCS – minimally conscious state, UWS – unresponsive wakefulness syndrome

Table S6. Individual clinical and behavioral patient characteristics								
Patient ID[1]	Trial drug	FOUR before	FOUR after	SECONDs before	SECONDs after	DoC before	DoC after	Improved arousal
1	Apomorphine	8	-	1	-	UWS	UWS	No
1	Placebo	7	-	1	-	UWS	UWS	No
1	Methylphenidate	7	-	1	-	UWS	UWS	No
2	Methylphenidate	11	-	1	-	UWS	UWS	No
2	Apomorphine	10	-	1	-	UWS	UWS	No
2	Placebo	11	-	1	-	UWS	UWS	No
3	Methylphenidate	5	-	5	-	MCS minus	MCS minus	No
3	Apomorphine	7	-	4	-	MCS minus	MCS minus	No
3	Placebo	13	-	6	-	MCS minus	MCS minus	No
4	Apomorphine	10	-	1	-	UWS	UWS	No
4	Methylphenidate	8	-	1	-	UWS	UWS	No
4	Placebo	5	-	0	-	Coma	Coma	No
5	Apomorphine	12	-	5	-	MCS minus	MCS minus	No
6	Placebo	11	-	2	-	MCS minus	MCS minus	No
6	Methylphenidate	11	-	5	-	MCS minus	MCS minus	No
7	Methylphenidate	8	-	1	-	UWS	UWS	No
8	Placebo	8	-	1	-	UWS	UWS	No
8	Apomorphine	8	-	1	-	UWS	UWS	No
8	Methylphenidate	10	-	6	-	MCS plus	MCS plus	No

9	Apomorphine	9	-	1	-	UWS	UWS	No
9	Methylphenidate	7	-	0	-	Coma	Coma	No
9	Placebo	13	-	6	-	MCS plus	MCS plus	No
10	Methylphenidate	11	-	4	-	MCS minus	MCS minus	No
10	Apomorphine	11	-	4	-	MCS minus	MCS minus	No
10	Placebo	11	-	4	-	MCS minus	MCS minus	No
11	Placebo	3	-	0	-	Coma	Coma	No
11	Methylphenidate	3	-	0	-	Coma	Coma	No
**12***	**Methylphenidate**	**8**	**13**	**1**	**6**	**UWS**	**eMCS**	**Yes**
12	Placebo	12	-	6	-	MCS plus	MCS plus	No
13	Placebo	8	-	1	-	UWS	UWS	No
13	Apomorphine	8	-	1	-	UWS	UWS	No
14	Placebo	13	-	6	-	MCS plus	MCS plus	No
15	Placebo	10	-	2	-	MCS minus	MCS minus	No
**16***	**Apomorphine**	**11**	**12**	**2**	**6**	**MCS minus**	**MCS plus**	**Yes**
17	Apomorphine	11	-	4	-	MCS minus	MCS minus	No
17	Placebo	11	-	4	-	MCS minus	MCS minus	No
17	Methylphenidate	11	-	4	-	MCS minus	MCS minus	No
**18***	**Methylphenidate**	**13**	**14**	**6**	**6**	**MCS plus**	**MCS plus**	**Yes**
18	Placebo	13	-	6	-	MCS plus	MCS plus	No

18	Methylphenidate	13	-	6	-	MCS plus	MCS plus	No
19	Methylphenidate	7	-	0	-	Coma	Coma	No
19	Apomorphine	7	-	0	-	Coma	Coma	No
19	Placebo	10	-	1	-	UWS	UWS	No
**20***	**Methylphenidate**	**10**	**14**	**4**	**6**	**MCS minus**	**MCS plus**	**Yes**
20	Placebo	8	-	0	-	Coma	Coma	No
**20***	**Apomorphine**	**9**	**14**	**1**	**6**	**UWS**	**MCS plus**	**Yes**
**21***	**Apomorphine**	**11**	**13**	**1**	**6**	**UWS**	**MCS plus**	**Yes**
21	Apomorphine	11	-	6	-	MCS plus	MCS plus	No
**21***	**Methylphenidate**	**13**	**14**	**6**	**6**	**MCS plus**	**MCS plus**	**Yes**
22	Apomorphine	11	-	5	-	MCS minus	MCS minus	No
22	Methylphenidate	10	-	1	-	UWS	UWS	No
22	Placebo	8	-	1	-	UWS	UWS	No
23	Apomorphine	8	-	1	-	UWS	UWS	No
23	Methylphenidate	8	-	1	-	UWS	UWS	No
24	Apomorphine	14	-	4	-	MCS minus	MCS minus	No
24	Placebo	14	-	4	-	MCS minus	MCS minus	No
25	Placebo	6	-	1	-	UWS	UWS	No
**26***	**Methylphenidate**	**11**	**12**	**5**	**5**	**MCS minus**	**MCS minus**	**Yes**
27	Apomorphine	5		0	-	Coma	Coma	No
27	Placebo	3	-	0	-	Coma	Coma	No

28	Methylphenidate	7	-	0	-	Coma	Coma	No
28	Placebo	10	-	1	-	UWS	UWS	No
29	Methylphenidate	12	-	4	-	MCS minus	MCS minus	No
**29***	**Apomorphine**	**15**	**16**	**4**	**6**	**MCS minus**	**MCS plus**	**Yes**
30	Methylphenidate	5	-	0	-	Coma	Coma	No
30	Apomorphine	5	-	0	-	Coma	Coma	No
30	Placebo	5	-	0	-	Coma	Coma	No
31	Apomorphine	8	-	0	-	UWS	UWS	No
31	Placebo	8	-	1	-	UWS	UWS	No
31	Methylphenidate	8	-	1	-	UWS	UWS	No
32	Apomorphine	8	-	1	-	UWS	UWS	No
32	Methylphenidate	8	-	1	-	UWS	UWS	No
33	Placebo	12	-	4	-	MCS minus	MCS minus	No
33	Apomorphine	12	-	4	-	MCS minus	MCS minus	No
33	Methylphenidate	12	-	4	-	MCS minus	MCS minus	No
**34***	**Placebo**	**7**	**8**	**1**	**7**	**UWS**	**MCS plus**	**Yes**
34	Methylphenidate	8	-	7	-	MCS plus	MCS plus	No
35	Placebo	10	-	1	-	UWS	UWS	No
35	Apomorphine	10	-	1	-	UWS	UWS	No
35	Methylphenidate	10	-	1	-	UWS	UWS	No
36	Placebo	8	-	5	-	MCS minus	MCS minus	No

37	Apomorphine	11	-	4	-	MCS minus	MCS minus	No
37	Placebo	11	-	4	-	MCS minus	MCS minus	No
38	Methylphenidate	13	-	6	-	MCS plus	MCS plus	No
38	Methylphenidate	13	-	6	-	MCS plus	MCS plus	No
39	Apomorphine	10	-	1	-	UWS	UWS	No
39	Apomorphine	10	-	1	-	UWS	UWS	No
39	Methylphenidate	10	-	1	-	UWS	UWS	No
40	Placebo	7	-	0	-	Coma	Coma	No
40	Apomorphine	9	-	2	-	MCS minus	MCS minus	No
40	Methylphenidate	12	-	4	-	MCS minus	MCS minus	No
41	Apomorphine	5	-	0	-	Coma	Coma	No
42	Methylphenidate	8	-	1	-	UWS	UWS	No
42	Apomorphine	5	-	0	-	Coma	Coma	No
42	Placebo	10	-	1	-	UWS	UWS	No
**43***	**Methylphenidate**	**15**	**16**	**6**	**6**	**MCS plus**	**MCS plus**	**Yes**
43	Placebo	15	-	6	-	MCS plus	MCS plus	No
43	Apomorphine	15	-	6	-	MCS plus	MCS plus	No
44	Placebo	15	-	6	-	MCS plus	MCS plus	No
45	Apomorphine	7	-	1	-	UWS	UWS	No
45	Methylphenidate	10	-	1	-	UWS	UWS	No

45	Placebo	12	-	4	-	MCS minus	MCS minus	No
46	Placebo	7	-	4	-	MCS minus	MCS minus	No
46	Methylphenidate	13	-	6	-	MCS plus	MCS plus	No
47	Methylphenidate	10	-	5	-	MCS minus	MCS minus	No
48	Apomorphine	8	-	1	-	UWS	UWS	No
48	Methylphenidate	8	-	1	-	UWS	UWS	No
49	Placebo	10	-	1	-	UWS	UWS	No
**49***	**Methylphenidate**	**10**	**10**	**1**	**5**	**UWS**	**MCS minus**	**Yes**
49	Apomorphine	10	-	5	-	MCS minus	MCS minus	No
50	Apomorphine	8	-	1	-	UWS	UWS	No
50	Placebo	8	-	1	-	UWS	UWS	No

[1] Each patient ID corresponds to multiple sessions, with repeated IDs indicating separate, chronologically ordered sessions.
*** Patients with improved clinical arousal, with or without a shift toward a higher consciousness level category.**

DoC – disorders of consciousness, FOUR – full outline of unresponsiveness scale, MCS – minimally conscious state, SECONDs – simplified evaluation of consciousness disorders scale, UWS – unresponsive wakefulness syndrome

Table S7. Neuroimaging of brain injury from 50 DoC patients

ID 1	
**Scan Type**	**CT cerebrum**
Etiology	Ischemic-embolic brain infarction post-surgery for aortic dissection
Supratentorial	- Watershed lesions: bilateral hypodensities, largest in frontal lobes, with hemorrhagic transformation<br>- Corpus callosum: right splenium hypodensity with hemorrhagic transformation<br>- Mass effect: obliterated surface sulci<br>- Ventricular system: compressed left lateral ventricle and anterior horn
Infratentorial	- Mesencephalon: small right hemorrhage<br>- Cerebellar: bi-hemispheric infarct-suspicious hypodensities

ID 2	
**Scan Type**	**MRI cerebrum with and without contrast**
Etiology	WHO grade 1 tumor in 3rd ventricle, surgically treated
Supratentorial	- Residual tumor: small contrast enhancement at upper resection edge<br>- Fornix: bilateral posterior lesions<br>- Corpus callosum: edematous, FLAIR hyperintense splenium<br>- Ischemia: three small punctiform lesions in left frontal lobe<br>- Perifocal edema: bifrontal, more on the left<br>- Ventricular system: slightly enlarged
Infratentorial	No findings

ID 3	
**Scan Type**	**MRI cerebrum with and without contrast**
Etiology	HSV 1 encephalitis
Supratentorial	- Temporal lobe: bilateral mesial hyperintensity, more on the right<br>- Insular: bilaterally hyperintensity, more on the right<br>- Hemorrhagic transformation: right mesial temporal lobe, insula, and Sylvian fissure<br>- Thalamus: discrete central/lower right signal changes
Infratentorial	No findings
Contrast enhancement	- Temporal lobe: bilateral mesial enhancement, more on right<br>- Insula: bilateral gyriform enhancement, more on right<br>- Periventricular: bilateral frontal enhancement

ID 4	

Scan Type	MRI cerebrum
Etiology	Hypoxic-ischemic injury after cardiac arrest
Supratentorial	- Parietal lobe: mesial cortical edema bilaterally (more on the left), and diffusion restriction<br>- Temporal lobe: scattered cortical edema<br>- White matter: symmetrical biparietal involvement<br>- Corpus callosum: posterior diffusion restriction<br>- Thalamus: bilateral signal increase, more on the left
Infratentorial	No findings

ID 5	
**Scan Type**	**CT cerebrum without and with contrast, CT angiography**
Etiology	Hypoxic-ischemic brain injury after cardiac arrest
Supratentorial	- Brain parenchyma: no focal changes<br>- Ventricles system: slim, midline<br>- Surface sulci: slim<br>- Gray-white matter differentiation: preserved
Infratentorial	No findings
Vascular	- Cerebral vessels: patent, normal caliber, no thrombosis or stenosis<br>- Neck vessels: patent, no dissection, stenosis, or thrombosis

ID 6	
**Scan Type**	**CT cerebrum without and with contrast, CT angiography**
Etiology	Ischemic stroke
Supratentorial	- Occipital and temporal lobes: extensive infarct<br>- Thalamus: bilateral infarcts<br>- Caudate nucleus: old lacunar infarct, left<br>- Insula: possibly enlarged perivascular spaces, left
Infratentorial	- Cerebellum: extensive infarct<br>- Mesencephalon: significant infarct, mainly left<br>- Pons-mesencephalon junction: left infarct<br>- Medulla oblongata: infarcts<br>- Middle cerebellar peduncles: infarcts, mainly left
Vascular	- Left vertebral artery: no contrast filling in V4 segment<br>- Prepontine and left quadrigeminal cisterns: slightly decreased space

ID 7	
**Scan Type**	**CT cerebrum**
Etiology	Hypoxic-ischemic brain injury after cardiac arrest
Supratentorial	- Gray-white matter differentiation: obliterated<br>- Surface sulci: effacement<br>- Ventricular system: midline, decreasing size
Infratentorial	- Gray-white matter differentiation: blurred<br>- Cerebellar surface sulci: increasing effacement

ID 8	
**Scan Type**	**CT cerebrum**
Etiology	Carbon monoxide poisoning
Supratentorial	- Corona radiata: lacunar infarct, left<br>- Thalamus: old lacunar infarct, left<br>- Caudate nucleus head: old lacunar infarct, left<br>- Anterior cingulate: old lacunar infarct, right<br>- Ventricular system: midline, widened<br>- Third ventricle: 16 mm, consistent with central atrophy
Infratentorial	- Pons: old lacunar infarct, left<br>- Cerebellar hemisphere: old infarct (posteromedial, caudal), left

ID 9	
**Scan Type**	**CT cerebrum**
Etiology	Hypoxic-ischemic brain injury after cardiac arrest
Supratentorial	Periventricular white matter: discrete non-confluent leukoaraiosis
Infratentorial	Cerebellar hemisphere: old infarct sequelae, right

ID 10	
**Scan Type**	**CT cerebrum**
Etiology	Traumatic brain injury
Supratentorial	- Right frontoparietal: craniotomy site with evacuated SDH<br>- Subdural space: resolving acute-on-chronic subdural hematoma (~15 mm)<br>- Surface sulci: effacement of right hemisphere<br>- Frontal lobe: mesial/rostral infarct sequelae, left
Infratentorial	No findings

ID 11	
**Scan Type**	**MRI cerebrum**
Etiology	Encephalopathy, respiratory failure (COVID-19)
Supratentorial	- No diffusion-restricted areas or current ischemia<br>- No mass effect<br>- No signs of bleeding, sequelae, or microbleeds<br>- White matter: discrete punctate subcortical gliosis<br>- Ventricular system: midline, age-appropriate
Infratentorial	No findings

ID 12	
**Scan Type**	**MRI cerebrum with and without contrast**
Etiology	HSV-2 encephalitis
Supratentorial	- Temporal lobe, right: mesial (incl. hippocampus) hyperintensity, edema<br>- Insula, right: hyperintensity, edema<br>- Frontal lobe: slightly increased signal in basal region
Infratentorial	- Mesencephalon: bilateral lower hyperintensity<br>- Pons: central and posterior hyperintensity
Contrast enhancement	- Temporal lobe: mesial enhancement, right

ID 13	
**Scan Type**	**MRI cerebrum**
Etiology	Perioperative hypovolemic shock leading to watershed infarcts (aortic surgery)
Supratentorial	- Corona radiata: bilateral diffusion restriction, subacute watershed infarcts<br>- Parietal lobe: infarct extending to cortex, left<br>- Occipital lobes: bilateral cortical diffusion restriction, more on the left<br>- Caudate nuclei: bilateral punctuate diffusion restrictions<br>- Thalamus: possible diffusion restriction at the top, left<br>- White matter: frontal punctate/confluent gliosis near ventricles (Fazekas 1-2, likely leukoaraiosis)
Infratentorial	Cerebellum: small diffusion restriction, left

ID 14	
**Scan Type**	**CT cerebrum**
Etiology	Perioperative hypovolemic shock with ischemic anoxic encephalopathy (heartmate)
Supratentorial	- Frontal lobe: infarct, reduced gray-white matter discrimination, left<br>- Precentral gyrus: reduced gray-white matter discrimination, left<br>- Superior frontal gyrus: reduced gray-white matter discrimination, left<br>- Lacunar: old infarcts
Infratentorial	No findings

ID 15	
**Scan Type**	**MRI cerebrum**
Etiology	Hypoxic-ischemic brain injury after cardiac arrest, traumatic brain injury
Supratentorial	- Diffusion-weighted imaging: no restriction, no acute ischemia<br>- Ventricles: midline<br>- Hemorrhage: no large acute bleeding
Infratentorial	No findings

ID 16	
**Scan Type**	**MRI cerebrum**
Etiology	Cerebral embolic strokes following aortic dissection repair
Supratentorial	- Cerebrum: subacute infarcts in all lobes (cortical, subcortical, and deep white matter)<br>- Occipital lobe: largest infarct, located mesial lobe on the right
Infratentorial	- Cerebellum: multiple subacute infarcts<br>- Pons: small left-sided subacute infarct

ID 17	
**Scan Type**	**MRI cerebrum**
Etiology	Left MCA infarct with hemorrhagic transformation
Supratentorial	- Left cerebral hemisphere: infarct in MCA territory, central and posterior<br>- Mass effect: 9 mm midline shift, left lateral ventricle compression<br>- Ventricular system: right lateral ventricle slightly full, blood in posterior horn<br>- Subarachnoid hemorrhage: residual blood from previous hemorrhage<br>- Corona radiata & central parieto-occipital region: two small punctate ischemic lesions, right<br>- Cerebrum: small hypointense lesions in both hemispheres (cavernomas)
Infratentorial	- Mesencephalon: subtle left-sided hyperintense signal changes<br>- Pons: cavernoma in left anterior pons and in central pons

ID 18	
**Scan Type**	**CT cerebrum**
Etiology	Intracerebral hemorrhage
Supratentorial	- Thalamus: left-sided hemorrhage<br>- Ventricular system: blood in both posterior horns and third ventricle, slightly enlarged<br>- Bithalamic region: hypodensities<br>- Basal ganglia: bilateral lacunar infarct sequelae
Infratentorial	- Mesencephalon: hemorrhage and edema in ventral part

ID 19	
**Scan Type**	**MRI cerebrum with and without contrast**
Etiology	HSV-1 encephalitis
Supratentorial	- Right hemisphere: DWI hyperintensity (insula, perisylvian frontotemporal region, temporal pole, uncus, hippocampus). Corresponding FLAIR changes with slight subcortical involvement.<br>- Parahippocampal gyrus: small right-sided DWI hyperintensity<br>- Periventricular region: signal changes (possible past ventriculitis)<br>- White matter: small non-specific bilateral changes<br>- Microbleeds: right corona radiata and posterior parietal lobe<br>- Ventricles: Midline, slightly wide (3rd ventricle: 10 mm)
Infratentorial	- Pons: suspected small right-sided telangiectasia<br>- Cerebellum: bilateral lacunar infarct sequelae
Contrast enhancement	- Right insula and adjacent cortex: subtle leptomeningeal enhancement<br>- Right temporal pole, uncus, and occipital lobe: punctate cortical enhancement

ID 20	
**Scan Type**	**MRI cerebrum with and without contrast**
Etiology	Intracerebral hemorrhage, suspected metastasis from breast cancer
Supratentorial	- Sylvian fissure: right microhemorrhage<br>- Ventricles: slim without transependymal edema, 3rd ventricle lesion (3.7 x 2 x 1 cm)
Infratentorial	Mesencephalon/pons: left-sided large lesion (4 x 2.3 x 2.2 cm), compressing 4th ventricle

ID 21	
**Scan Type**	**MRI cerebrum**
Etiology	Post-cardiac arrest ischemic anoxic encephalopathy, bilateral infarcts
Supratentorial	- Cerebrum: bilateral subacute ischemia, mainly in watershed areas<br>- Parieto-occipital area: right-sided FLAIR-positive area, and left-sided microbleeds<br>- Frontal lobes: bilateral small FLAIR-positive areas with diffusion restriction<br>- Centrum semiovale: bilateral small FLAIR-positive areas with diffusion restriction<br>- Thalamus: left-sided FLAIR-positive area with diffusion restriction<br>- Occipital lobe: left-sided FLAIR-positive area with diffusion restriction<br>- Cortical sulci: accentuated
Infratentorial	- Pons: right-sided punctate subacute ischemia<br>- Cerebellum: punctate subacute ischemia (right), possible punctate ischemia (left), infarct sequelae in right hemisphere

ID 22	
**Scan Type**	**MRI neuroaxis with and without contrast**
Etiology	HSV-1 encephalitis
Supratentorial	- Cerebral hemispheres: bilateral subdural hygromas<br>- Thalamus: small lacunar old infarct, left
Infratentorial	Pons: small lacunar old infarct, anterior midline
Contrast Enhancement	Dura: mild intracranial thickening and enhancement

ID 23	
**Scan Type**	**CT cerebrum**
Etiology	Hypoxic-ischemic brain injury after cardiac arrest, traumatic brain injury
Supratentorial	- Parieto-occipital lobes: large infarcts in watershed areas bilaterally<br>- Frontal lobes: cortical infarcts bilaterally, involving precentral gyrus<br>- Temporal lobe: right-sided infarct in anterior watershed area<br>- Basal ganglia: lacunar infarcts bilaterally<br>- Thalami: lacunar infarcts bilaterally
Infratentorial	Cerebellar hemispheres: infarct demarcation with increasing edema

ID 24	
**Scan Type**	**MRI cerebrum**
Etiology	Ischemic stroke
Supratentorial	- Susceptibility-weighted imaging: diffuse microbleeds in a "walnut kernel pattern"<br>- Cerebrum: FLAIR-positive signal in white matter, subcortical regions, right splenium
Infratentorial	Cerebellum: FLAIR-positive signal in middle peduncle

ID 25	
**Scan Type**	**CT cerebrum**
Etiology	Post-operative complications following aortic pseudoaneurysm repair
Supratentorial	White matter: Periventricular leukoariosis
Infratentorial	No findings

ID 26	
**Scan Type**	**MRI cerebrum**
Etiology	Post-status epilepticus, suspected intracerebral pathology/anoxic damage
Supratentorial	- Parietal lobes: mild diffusion restriction and FLAIR hyperintensity bilaterally, left > right<br>- Fronto- temporal lobes: left-sided mild substance loss, gliosis, and basal hemosiderin<br>- White matter: mild periventricular and subcortical gliosis<br>- Cerebrum: cortical atrophy
Infratentorial	Cerebellum: atrophy

ID 27	
**Scan Type**	**MRI cerebrum**
Etiology	Ischemic stroke
Supratentorial	- Right cerebral hemisphere: large MCA infarct with laminar necrosis<br>- Basal ganglia: hemorrhage in right central area, and minor left-sided infarct sequelae<br>- Putamen: small infarct, left<br>- Frontal lobe: small right-sided infarct, and minor left-sided infarct sequelae<br>- Ventricular system: mild leftward shift, slight right-sided compression
Infratentorial	- Cerebral peduncle: small right infarct<br>- Cerebellar hemisphere: small left-sided punctate infarct near fourth ventricle

ID 28	
**Scan Type**	**MRI cerebrum**
Etiology	Peri-operative hypotension, multiple infarcts
Supratentorial	- External watershed zones: multiple infarcts bilaterally (ACA-MCA, MCA-PCA)<br>- Internal watershed zones: deep white matter infarcts bilaterally<br>- Basal ganglia: infarcts bilaterally<br>- Thalamus: infarcts bilaterally<br>- Temporal lobes: cortical and subcortical infarcts bilaterally<br>- Mass effect: slight narrowing of surface sulci
Infratentorial	- Cerebellar hemispheres: small infarcts, larger on the right

ID 29	
**Scan Type**	**CT cerebrum**
Etiology	Heart transplant surgery, post-operative
Supratentorial	- Intracranial hemorrhage: None<br>- Ischemic infarcts: none (fresh or old)<br>- Gray-white matter: preserved discrepancy<br>- Surface sulci: preserved<br>- Ventricular system: midline, slim
Infratentorial	No findings

ID 30	
**Scan Type**	**MRI cerebrum**
Etiology	Hypoxic-ischemic injury after cardiac arrest
Supratentorial	- Sylvian fissures: slightly widened<br>- Hippocampi: slightly increased signal bilaterally, no diffusion restriction<br>- Cortex and basal ganglia: no signal abnormalities
Infratentorial	No findings

ID 31	
**Scan Types**	**CT cerebrum without contrast**<br>**CT angiography**
Etiology	Metabolic disorder
Supratentorial	- Cerebral hemispheres: no fresh hemorrhage, infarcts or space-occupying pathology<br>- Surface sulci: preserved<br>- Ventricular system: midline, slim<br>- Basal cisterns: open
Infratentorial	No findings
Vascular	- Neck vessels: normal, no occlusion or dissection<br>- Carotid arteries: mild atherosclerotic calcifications (bulb and bifurcation), no stenosis<br>- Intracranial vessels: normal, patent, no aneurysms, or malformations

ID 32	
**Scan Types**	**No neuroimaging available**
Etiology	COVID-19

ID 33	
**Scan Type**	**CT cerebrum**
Etiology	Hydrocephalus, post-aneurysm coiling
Supratentorial	- Hydrocephalus: increased mamillopontine distance, transependymal edema<br>- Lateral ventricles: widened<br>- Frontal lobes: bilateral hypodensity/gliosis, right > left<br>- Frontoparietal: demarcation of infarct sequelae, left<br>- Surface sulci: universally poorly defined bilaterally
Infratentorial	- Cerebellar hemisphere: small old lacunar infarct sequelae, left<br>- 4th ventricle: 16 mm side-to-side

ID 34	
**Scan Type**	**MRI cerebrum**
Etiology	Ischemic stroke
Supratentorial	- Parietal lobe: medial infarct, left<br>- Occipital lobe: left-sided anterior infarct, and small hemosiderin deposit medially<br>- Thalamus: posterior infarct, left<br>-Basal ganglia: old infarct with anterior hemosiderin deposit, right<br>- Old infarct in right frontal and parietal lobe<br>- Frontoparietal lobes: old right-sided infarct<br>- White matter: scattered, slightly confluent bilateral FLAIR hyperintensities (Fazekas 2)<br>- Ventricles: 3rd ventricle (9 mm), mild central atrophy
Infratentorial	- Cerebellar hemispheres: bilateral infarcts, left > right<br>- Mesencephalon: left infarct<br>- Pons: left infarct<br>- Medulla oblongata: infarct with FLAIR-positive signal

ID 35	
**Scan Type**	**MRI cerebrum with and without contrast**
Etiology	Hypoxic-ischemic injury after cardiac arrest
Supratentorial	- Occipital lobe: small gyral and cortical enhancements (subacute infarct), right
Infratentorial	- Cerebellar hemispheres: small contrast-enhancing streaks (subacute infarct), mostly caudal

ID 36	
**Scan Type**	**CT cerebrum**
Etiology	Hypoxic-ischemic injury after cardiac arrest
Supratentorial	- Ventricular system: age-appropriate<br>- Surface sulci: preserved<br>- Gray-white matter: preserved discrepancy<br>- Corpus callosum: thinned
Infratentorial	No findings
Vascular	- Internal carotid and vertebral arteries: arteriosclerosis in bilaterally<br>- No dense artery sign

ID 37	
**Scan Type**	**MRI cerebrum with and without contrast**
Etiology	Traumatic brain injury
Supratentorial	- Cerebrum: interhemispheric hemorrhage and subarachnoid bleeding, scattered microhemorrhages<br>- Parietal lobe: right-sided sequelae post-craniotomy with substance loss and hemosiderin deposition<br>- Ventricular system: small blood sediment in posterior horns of lateral ventricles
Infratentorial	Scattered microhemorrhages

ID 38	
**Scan Type**	**CT cerebrum**
Etiology	Suspected infarct/bleeding following aortic dissection repair
Supratentorial	- Gray-white matter differentiation: preserved<br>- Ventricular system: midline, slim<br>- Surface sulci: preserved
Infratentorial	No findings

ID 39	
**Scan Type**	**MRI cerebrum**
Etiology	Lung transplantation with complicated peri- and postoperative course
Supratentorial	- Cerebral hemispheres: microhemorrhages in corticomedullary junction, deep white matter, cortex, cortically, basal ganglia, thalami, and corpus callosum<br>- Caudate nucleus: substance loss<br>- Periventricular leukoencephalopathy extending into centrum semiovale and subcortically
Infratentorial	- Cerebellar hemispheres: microhemorrhages, substance loss<br>- Mesencephalon: microhemorrhages<br>- Pons: microhemorrhages<br>- Cerebellar peduncles: microhemorrhages in the middle

ID 40	
**Scan Type**	**CT cerebrum**
Etiology	Hypoxic-ischemic injury after cardiac arrest
Supratentorial	- Gray-white matter differentiation:<br>- No acute or old infarcts, no hemorrhages<br>- Surface sulci: preserved<br>- Ventricular system: midline, 3rd ventricle (9 mm)
Infratentorial	No findings

ID 41	
**Scan Type**	**CT cerebrum**
Etiology	Septic shock, hypotensive ischemic encephalopathy
Supratentorial	- Cerebrum: right MCA infarct involving insular cortex<br>- Corona radiata: sequelae of lacunar infarct, left<br>- Sylvian fissure: calcification, right<br>- Ventricular system: midline, slim<br>- Surface sulci: accentuated, most pronounced frontoparietal
Infratentorial	No findings

ID 42	
**Scan Type**	**MRI cerebrum**
Etiology	Ischemic stroke
Supratentorial	- Cerebrum: Left MCA infarct, persistent reduced diffusion (mainly in white matter)<br>- Microhemorrhages: punctuate in the infarct area, no large hemorrhage<br>- Mass effect: mild, compressing left occipital horn<br>- Surface sulci: locally reduced
Infratentorial	- Brainstem: left mesencephalon and pons signal alteration (early Wallerian degeneration)

ID 43	
**Scan Type**	**CT cerebrum with and without contrast**
Etiology	Shunt dysfunction, obstructive hydrocephalus
Supratentorial	- Ventricular system: midline, enlarged lateral ventricles – especially temporal horns<br>- Occipital lobe: right-sided hypodense area<br>- Frontal lobe: area with underlying substance loss, right
Infratentorial	- 4th ventricle ballooned<br>- Posterior fossa: craniotomy sequelae, right<br>- Caudal aqueduct: very narrow

ID 44	
**Scan Type**	**CT cerebrum**
Etiology	Post-cardiac arrest anoxic-ischemic encephalopathy
Supratentorial	- Ventricular system: midline, slim<br>- Thalamus: lacunar infarct, right<br>- Leukoaraiosis
Infratentorial	No findings

ID 45	
**Scan Type**	**CT cerebrum**
Etiology	Post-surgery of abdominal aortic aneurysm
Supratentorial	- Cerebrum: no hemorrhages, infarcts, or space-occupying lesions<br>- Gray-white matter differentiation: preserved<br>- Surface sulci: age-appropriate<br>- Ventricular system: midline, slim, symmetrical
Infratentorial	No findings

ID 46	
**Scan Type**	**CT cerebrum**
Etiology	Aneurysmal SAH with subdural extension
Supratentorial	- Falx cerebri: left-sided parafalcine SDH (5mm)<br>- Temporal lobe: right contusion bleeding (32 x 24 x 24 mm)<br>- Mass effect: midline shift to the left (9 mm)<br>- Frontal lobe: two small hypodense areas medially, right
Infratentorial	- Mesencephalon/pons: contusion bleeding in right posterior part<br>- Ambient cistern: right-sided SAH<br>- Cerebellar hemispheres: SAH extending to upper spinal canal

ID 47	
**Scan Type**	**CT cerebrum**
Etiology	Multiple intracerebral hemorrhages
Supratentorial	- Basal ganglia: hemorrhage (40 x 33 x 21 mm) with perifocal edema<br>- Occipital lobe: hemorrhage mesial part, left (13 x 9 x 20 mm) with perifocal edema<br>- Ventricular system: midline, slightcompression of left lateral horn<br>- Surface sulci: preserved<br>- Frontal lobe: lacunar infarcts, right
Infratentorial	No findings

ID 48	
**Scan Type**	**CT cerebrum**
Etiology	Hypoxic-ischemic injury after cardiac arrest
Supratentorial	- Cerebrum: no hemorrhage, infarcts, or space-occupying lesions<br>- Globus pallidus: no obvious hypodensities bilaterally<br>- Gray-white matter differentiation: preserved<br>- Surface sulci: widened<br>- Ventricular system: midline, slightly widened
Infratentorial	No findings

ID 49	
**Scan Type**	**MRI cerebrum**
Etiology	Hypoxic-ischemic injury after cardiac arrest
Supratentorial	- Parietooccipital cortex: watershed infarct, right<br>- Basal ganglia: right- sided lacunar infarct, punctate bilateral microhemorrhages<br>- Motor cortex: bilateral diffusion restriction in motor cortex areas, right > left
Infratentorial	No findings

ID 50	
**Scan Type**	**MRI cerebrum**
Etiology	Intracerebral hemorrhage
Supratentorial	- Thalamus: severe edema bilaterally<br>- Cerebral crura: severe edema bilaterally, petechial hemorrhages (more on right)<br>- Ventricular system: midline, slim<br>- Surface sulci: preserved
Infratentorial	- Posterior midline: expected bleeding corresponding to surgical access<br>- Cerebellar hemispheres: bilateral edema mesially<br>- Pons: slight FLAIR-positive signal
